# Supplementary material for: Protocol for an intervention development and pilot implementation evaluation study of an e-health solution to improve newborn care quality and survival in two low-resource settings, Malawi and Zimbabwe: Neotree
Source: BMJ Open. 2022 Jul 5;12(7):e056605. doi: 10.1136/bmjopen-2021-056605 (PMC9258512; doi:10.1136/bmjopen-2021-056605)
Supplement: Supplementary data [file bmjopen-2021-056605supp001.pdf]

Supplementary File 1

Draft Focus Group Discussion (FGD) and Semi-Structured Interview Topic Guides with Healthcare Professionals (nurses and doctors)

Topic Guide 1: Baseline SSI

Interview guide for NeoTree semi-structured interview

Aim: to explore practice pre-implementation

Timing: pre-implementation (months 5-6)

Participants: Healthcare Professionals

Version: 1.0

Introduction

Hello, thank you very much for taking the time to speak to me today. My name is ______ and I work for _____________. This interview will probably last around 1 hour. As a reminder, I am talking to you today, as I work for a study that aims to improve the care given to sick and vulnerable babies in hospitals.

Before we start, can I check whether you have:

- been told about the study
- had an information sheet
- signed a consent form
- agreed to audio recording of the discussion

At this point, do you have any questions about the purpose of the study, or the documents you've been given [e.g. PIS/ consent form]? Is there anything that isn't clear?

I want to reassure you that I work for a research organisation [name] and not [name of health facility]. Anything you tell me today is confidential and will not be shared with your colleagues. All personal and identifying information (such as your name/names of others) mentioned will be removed and replaced with a code.

I just want to remind you that your participation in this interview is entirely voluntary [i.e. it is your choice]. If you do not want to answer a question you can just say 'pass' and we will move on to the next question.

I am interested to understand your role at [health facility] in the provision of neonatal care and how care is delivered. There are no right or wrong answers to these questions; I am just interested in your views so please answer honestly.

If you want to take a break or stop at any point, please tell me. And if you wish to withdraw from the study you are completely free to do so at any point.

1) Warm up questions

    a) What is your role on the unit? How long have you worked here? Where did you complete your training?

2) Staffing

    a) How many nurses, midwives, student nurses are on the unit, what qualifications, what are their roles?
    b) Is there a paediatrician in the unit? If not can you elaborate on the doctors' support you receive in the unit?
    c) What professional training is offered to nurse/ doctors?

3) Services and Procedures

    a) What health care services are provided in the neonatal ward? How linked with maternity ward?
    b) What are existing roles/ responsibilities/ practices for neonatal care? (from admission to discharge, i.e. who does what? when? where?)

4) Pathways and communication

    a) Can you describe the pathways for referrals into and out of the neonatal unit? How well do referral pathways work in practice? What could be improved?
    b) How would you describe your relationship with your co-workers, doctors, management? Any challenges in team working/ communication?
    c) How would you describe morale among HCWs on the unit? What could be improved?

5) MIS

    a) How are patient data captured currently (admission/ discharge/ lab results)? Any challenges with data capture?
    b) How are data used for quality improvement?

6) Equipment and supplies

    a) What machinery is available in the unit?
    b) What lab facilities are there? Drug supplies and consumables?
    c) What equipment/ supplies do you feel are lacking? Any problems with procurement?

7) Challenges in provision of neonatal care

    a) What do you feel are the main challenges to providing neonatal care in this hospital?
    b) How do you feel neonatal services could be improved?

8) Closing remarks

    a) Is there anything you would like to ask me?

BMJ Publishing Group Limited (BMJ) disclaims all liability and responsibility arising from any reliance placed on this supplemental material which has been supplied by the author(s)

Thank you so much, we really appreciate the time you've taken to participate in this study.

Topic Guide 2: Baseline FGD

Topic guide for NeoTree focus groups based on Theoretical Framework of Acceptability (TFA) and the Theoretical Domains Framework (TDF)

Timing of focus group: Pre-implementation (months 5-6)

Participants: Healthcare Professionals

Version: 1.0

Introduction

Hello, thank you very much for taking the time to speak to me today. My name is ______ and I work for _____________. This discussion will probably between 1-2 hours. As a reminder, I am talking to you today, as I work for a study that aims to improve the care given to sick and vulnerable babies in hospitals.

Before we start, can I check whether you have:

- been told about the study
- had an information sheet
- signed a consent form
- agreed to audio recording of the discussion

At this point, do you have any questions about the purpose of the study, or the documents you've been given [e.g. PIS/ consent form]? Is there anything that isn't clear?

I just want to remind you that your participation in this discussion is entirely voluntary [i.e. it is your choice].

I am interested in your views about the implementation of the NeoTree at [health facility]. Your opinions as health care workers at the frontline of newborn care are very important to us. There are no right or wrong answers to these questions, just differing points of views. Please feel free to share your point of view even if it differs from what others have said. Keep in mind that we are just as interested in negative comments as positive comments.

Please give everyone a chance to speak, and please do not share what we discuss today outside this group. Please say your ID number before you speak and do not say names during the discussion as we want to keep them anonymised.

If you want to take a break or stop at any point, please tell me. And if you wish to withdraw from the study you are completely free to do so at any point.

Before we start, do you have any questions for me?

1) Warm up questions

What is your role at this hospital? How long have you worked here?

2) Introduction to NeoTree [demonstration of the NeoTree]

Today I'd like us to discuss the NeoTree, which is a digital health intervention that we are planning to introduce at [health facility] to improve the quality of newborn care. The NeoTree is an app. It allows you to enter in all the clinical information about the baby on admission and discharge. It gives you guidance on what is likely to be wrong with the baby and how to best treat and manage the baby, based on international guidelines. Whilst the baby is on the newborn care unit it will also provide an electronic linkage to any tests that are done to check for infection. The NeoTree also stores the clinical information for all of the babies and then feeds back to healthcare workers and the hospital on things like how many babies were admitted each month and what was wrong with them so that they can best plan the services needed.

Do you have any questions?

3) Knowledge & Skills (TDF)

a) How easy or difficult do you think it would be to use the NeoTree at [health facility]?

Prompt: What in particular would be easy? What in particular would be difficult?

b) Do you think you would need any additional skills or training to use NeoTree?

4) Beliefs about consequences (TDF)

a) What do you perceive to be the benefits/positives of using NeoTree?

Prompt: for: you as nurses/ doctors, other colleagues/roles and for patients & families?

b) Would there be any drawbacks?

Prompt: for you as nurses/ doctors, other colleagues/roles and for patients and families?

c) Overall, do you think the pros would outweigh the cons?

5) Burden (TFA) + Social Professional Role/Identity (TDF)

a) Do you think using the NeoTree would create extra work for you? In what way?

b) Do you feel it is would be your responsibility to use a tool like this? Who else might be responsible for completing the information in NeoTree or using it to guide decisions around care?

6) Social influences (TDF)

a) To what extent would the views of others influence whether you use the NeoTree?

For example the opinions of other nurses in the unit, other doctors, senior hospital managers, the NeoTree project management team, parents/guardians?

7) Opportunity costs (TFA)

Is there anything that you feel you would have to give up in order to use the NeoTree?

8) Ethicality (TFA)

a) Do you feel the NeoTree would be safe? For you and for babies?

b) Do you feel introducing the NeoTree would be fair?

9) Self-efficacy (TFA) & Beliefs about capabilities (TDF)

a) How confident would you be using the NeoTree? Is there anything in particular you would not be confident about?

b) Is there anything that could help you become more confident?

10) Environmental context and resources (TDF)

a) Do you think you would have sufficient resources to implement the NeoTree?

11) Goals (TDF)

a) Compared to other things you have to do, where does introducing the NeoTree fit in in terms of a priority? What would be the competing priorities?

b) Do you have any targets/goals for practice? How would the NeoTree fit in with that if at all?

12) Perceived effectiveness (TFA) + optimism (TDF)

In your view, how likely is the NeoTree to improve quality of newborn care?

13) Intentions (TDF)

a) Would you like to use the NeoTree? Can you explain your reasons why/why not?

b) To what extent do you intend to use it?

14) Barriers/ facilitators (general)

a) What do you think might be some of the barriers to introducing the NeoTree at [health facility]?

b) Is there anything that might make it easier to implement the NeoTree at [health facility]?

To summarise, the main points from our discussion are (facilitator to summarise key points). Is there anything else you'd like to add? Is there anything you'd like to ask me?

Thank you so much. We really appreciate the time you've taken to participate in this study.

Topic Guide 3: FGD - basic functionality of the NeoTree

Topic guide for NeoTree focus groups based on Theoretical Framework of Acceptability (TFA) and the Theoretical Domains framework (TDF)

Target behaviours – digital documentation on NeoTree, following emergency diagnostic guidelines, receiving educational support

Timing of focus group: implementation phase (months 6/7)

Participants: Healthcare Professionals

Version 1.0

Introduction

Hello, thank you very much for taking the time to speak to me today. My name is ______ and I work for ______________. This discussion will probably between 1-2 hours. As a reminder, I am talking to you today, as I work for a study that aims to improve the care given to sick and vulnerable babies in hospitals.

Before we start, can I check whether you have:

- been told about the study
- had an information sheet
- signed a consent form
- agreed to audio recording of the discussion

At this point, do you have any questions about the purpose of the study, or the documents you've been given [e.g. PIS/ consent form]? Is there anything that isn't clear?

I just want to remind you that your participation in this discussion is entirely voluntary [i.e. it is your choice].

I am interested in your views about the implementation of the NeoTree at [health facility]. Your opinions as health care workers at the frontline of newborn care are very important to us. There are no right or wrong answers to these questions, just differing points of views. Please feel free to share your point of view even if it differs from what others have said. Keep in mind that we're just as interested in negative comments as positive comments

Please give everyone a chance to speak, and please do not share what we discuss today outside this group. Please say your ID number before you speak and do not say names during the discussion as we want to keep them anonymised.

If you want to take a break or stop at any point, please tell me. And if you wish to withdraw from the study you are completely free to do so at any point.

Before we start, do you have any questions for me?

1) Warm up questions

What is your position at this hospital? How long have you worked here?

2) Intervention coherence (TFA)

We are going to ask you a few questions about NeoTree which as you know is a new digital platform we have started using on the ward.

a) Can you talk me through your understanding of what NeoTree is? What it is for?

Optional Prompt: What are the functions / objectives of the NeoTree?

3) Barriers (general)

a) What in general do you think are the barriers to using NeoTree in day to day practice?
b) Is there anything that made it easier or encouraged you to use NeoTree in day to day practice?

4) Knowledge & Skills (TDF)

a) Overall, 'how easy or difficult is it to use NeoTree?'

Prompt: What in particular is easy? What in particular is difficult?

b) Do you think you need any additional skills or training to use NeoTree?

5) Beliefs about consequences (TDF)

a) How does using NeoTree differ to the previous paper-based system for documentation?
b) Are there any benefits/positives of using NeoTree?

Prompt: for you as nurses, other colleagues/roles and for patients & families?

c) Are there any draw-backs/negatives?

Prompt: for you as nurses, other colleagues/roles and for patients and families?

d) Overall, do you think the pros outweigh the cons?

6) Burden (TFA)/ Beliefs about consequences

a) How does it affect your capacity to do your job? Does it help or hinder you to do your job?
b) How much effort does it require to use NeoTree compared to the paper form you were using before?
    - does it require more time? more support from colleagues?

7) Affective attitude (TFA) + Emotion (TDF)

a) Do you like using NeoTree? What in particular do you like or dislike?

Prompt: Any concerns or worries about NeoTree?

8) Social influences (TDF)

a) To what extent do the views of others influence if and how you use the NeoTree? For example the opinions of other nurses in the unit, the NeoTree ambassador, doctors, the NeoTree project management team, parents/guardians?

b) Has the NeoTree changed team working and communication in the unit in any way?

Prompt: communication, roles, responsibilities?

9) Opportunity costs (TFA)

Is there anything that you feel you must give up in order to use the NeoTree?

10) Ethicality (TFA) Social/Professional role & identity (TDF)

a) Do you feel it is safe to use NeoTree? For you and for babies?

b) How does NeoTree fit in with your ways of working? Do you feel it is your responsibility to use a tool like this? Who else might be responsible for completing the information in NeoTree or using it to guide decisions around care?

11) Self-efficacy (TFA) Beliefs about capabilities (TDF)

a) How confident are you in using the NeoTree? Is there anything in particular you are not confident about?

b) Is there anything that could help you become more confident?

12) Environmental context and resources (TDF)

a) Do you have enough resources to use the NeoTree?

13) Memory, attention, decision making (TDF)

a) Do you use NeoTree to guide your decision-making? How so?

Does it make things easier/more difficult?

b) Have you ever forgotten to use the NeoTree?

If yes - in what kind of situations? and how can this be avoided in future?

c) Are there ever any instances when you decided to deviate from recommendations in NeoTree? Talk me through this….

d) Are there ever any instances where you decided not to use NeoTree? If so, can you talk me through this…

14) Goals (TDF)

a) How important is using NeoTree for you? And why?

b) Compared to other things you have to do, where does completing the NeoTree forms and using NeoTree fit in in terms of a priority? What are the competing priorities?

c) Do you have any targets/goals for practice? How does NeoTree fit in with that if at all etc

15) Perceived effectiveness (TFA) + TDF optimism

a) In your view, how likely is the NeoTree to improve quality of newborn care?

16) Reinforcement (TDF)

a) What positive experiences have you had with the NeoTree that would encourage you to use it again? Are there any negative experiences that would discourage you from using NeoTree again?

b) Are there any incentives / rewards / pressures to use NeoTree?

17) Intentions (TDF)

a) Do you intend to use the NeoTree in your day-to-day practice?

18) Behavioural regulation (TDF)

a) To what extent has using NeoTree become habitual or something you do without thinking, day to day? Why/how so?

b) Do you ever review the data you enter into NeoTree? Use it to monitor practice? Discuss it with colleagues?

c) Have you ever encountered any problems using NeoTree? How did you overcome these?

d) Is there anything you think we can do to improve the implementation of the NeoTree on the ward?

To summarise, the main points from our discussion are (facilitator to summarise key points). Is there anything else you'd like to add? Is there anything you'd like to ask me?

Thank you so much. We really appreciate the time you've taken to participate in this study.

Topic Guide 4: FGD -data dashboard

Topic guide for NeoTree focus groups based on Theoretical Framework of Acceptability (TFA) and the Theoretical Domains Framework (TDF)

Target behaviours – Interpreting data from data dashboard; changing clinical practise/behaviours to achieve agreed quality improvement goals.

Timing of focus group: implementation phase (months 10/11)

Participants: Healthcare Professionals

Version 1.0

Introduction

Hello, thank you very much for taking the time to speak to me today. My name is ______ and I work for _____________. This discussion will probably between 1-2 hours. As a reminder, I am talking to you today, as I work for a study that aims to improve the care given to sick and vulnerable babies in hospitals.

Before we start, can I check whether you have:

- been told about the study
- had an information sheet
- signed a consent form
- agreed to audio recording of the discussion

At this point, do you have any questions about the purpose of the study, or the documents you've been given [e.g. PIS/ consent form]? Is there anything that isn't clear?

I just want to remind you that your participation in this discussion is entirely voluntary [i.e. it is your choice].

I am interested in your views about the implementation of the NeoTree at [health facility]. Your opinions as health care workers at the frontline of newborn care are very important to us. There are no right or wrong answers to these questions, just differing points of views. Please feel free to share your point of view even if it differs from what others have said. Keep in mind that we're just as interested in negative comments as positive comments

Please give everyone a chance to speak, and please do not share what we discuss today outside this group. Please say your ID number before you speak and do not say names during the discussion as we want to keep them anonymised.

If you want to take a break or stop at any point, please tell me. And if you wish to withdraw from the study you are completely free to do so at any point.

Before we start, do you have any questions for me?

1) Intervention coherence (TFA) / Knowledge (TDF)

   a) Have you noticed any recent changes to the NeoTree? Any recent additions?

      [show data dashboard]

   b) Can you talk me through the dashboard and what you think it hopes to achieve?

2) Knowledge & Skills (TDF)

   a) Overall, how easy or difficult is it to understand the information presented in the dashboards? What in particular is easy? What in particular is difficult?

   b) What would make the dashboards easier to understand?

3) Beliefs about consequences (TDF)

   a) How might having these dashboards change practice, if at all?

      Prompt- how do they influence your decision making/actions?

   b) What are the benefits of having these dashboards? Any downsides?

4) Burden (TFA)

   a) To what extent do you use the dashboard in your day-to-day job? Talk me through this

   b) Has using it had an impact on your workload in any way? i.e. generated more tasks, needed time away from other responsibilities etc

5) Affective attitude (TFA) & Emotion (TDF)

   a) Do you like using the data dashboards ? What in particular do you like or dislike?

   b) How helpful is the dashboard? Any concerns or worries?

6) Social influences (TDF)

   a) To what extent do the views of others influence if and how you use the dashboard? For example the opinions of other nurses in the unit, the NeoTree ambassador, doctors, the NeoTree project management team, parents/guardians?

   b) Has the NeoTree changed team working in the unit in any way?

      Prompt: communication, roles, responsibilities?

7) Opportunity costs (TFA)

   a) Is there anything that you feel you must give up in order to use the data dashboard?

   b) Given the choice between the basic NeoTree and NeoTree with data dashboard which would you choose to use, and why?

8) Ethicality (TFA) & Social/Professional role & identity (TDF)

   a) Do you feel displaying data and quality improvement targets in the ward is fair? for you as a HCW?
   b) Do you feel it is your responsibility to use a tool like this? Who else might be responsible to use the data dashboards?

9) Self-efficacy (TFA) & Beliefs about capabilities (TDF)

   a) How confident are you using the data dashboard? What in particular are you more/less confident about?
   b) What could help improve your confidence?

10) Environmental context and resources (TDF)

   a) Do you have enough time to use the data on the data dashboard in your day-to-day practice?
   b) Do you have the necessary resources available to work towards achieving the dashboard recommendations and targets?

11) Memory, attention, decision making (TDF)

   a) Is there anything on the dashboards that grab your attention? What in particular?
   b) How easy/difficult is it to extract key information from the dashboard?
   c) Are there ever any instances when you decided to deviate from suggested behaviour and quality improvement target? Talk me through this….

12) Goals (TDF)

   a) How important is using the data dashboard for you? And why?
   b) Compared to other things you have to do, where does the data dashboard fit in terms of priority? What are the competing priorities?
   c) Do you have any targets/goals for practice? How does the data dashboard fit in with that if at all?

13) Perceived effectiveness (TFA) + Optimism (TDF)

   a) How likely is the data dashboard to improve quality of newborn care?

14) Reinforcement (TDF)

   a) What positive experiences have you had with the data dashboard that would encourage you to keep using it?
   b) What incentives / rewards / pressures are there to use the data dashboard and achieve behaviour change targets?

15) Intentions (TDF)

a) To what extent do you intend to use the dashboards in your everyday work? Can you explain your reasons?

16) Behavioural regulation (TDF)

a) To what extent has using the data dashboard become habitual (i.e. something that you do routinely) in your day to day practice? Why/how so?

b) Have you ever encountered any problems using the dashboards? What were these and how did you overcome these?

c) What can we do to improve the implementation of the dashboards on the ward??

To summarise, the main points from our discussion are (facilitator to summarise key points). Is there anything else you'd like to add? Is there anything you'd like to ask me?

Thank you so much. We really appreciate the time you've taken to participate in this study.

Topic Guide 5: FGD - activated non-emergency clinical algorithm

Topic guide for NeoTree focus groups based on Theoretical Framework of Acceptability (TFA) and the Theoretical Domains Framework (TDF)

Target behaviours – use of the non-emergency algorithm to diagnose and manage newborns with sepsis and HIE.

Timing of focus group: implementation phase (month 16)

Participants: Healthcare Professionals

Version: 1.0

Introduction

Hello, thank you very much for taking the time to speak to me today. My name is ______ and I work for _____________. This discussion will probably between 1-2 hours. As a reminder, I am talking to you today, as I work for a study that aims to improve the care given to sick and vulnerable babies in hospitals.

Before we start, can I check whether you have:

- been told about the study
- had an information sheet
- signed a consent form
- agreed to audio recording of the discussion

At this point, do you have any questions about the purpose of the study, or the documents you've been given [e.g. PIS/ consent form]? Is there anything that isn't clear?

I just want to remind you that your participation in this discussion is entirely voluntary [i.e. it is your choice].

I am interested in your views about the implementation of the NeoTree at [health facility]. Your opinions as health care workers at the frontline of newborn care are very important to us. There are no right or wrong answers to these questions, just differing points of views. Please feel free to share your point of view even if it differs from what others have said. Keep in mind that we're just as interested in negative comments as positive comments

Please give everyone a chance to speak, and please do not share what we discuss today outside this group. Please say your ID number before you speak and do not say names during the discussion as we want to keep them anonymised.

If you want to take a break or stop at any point, please tell me. And if you wish to withdraw from the study you are completely free to do so at any point.

Before we start, do you have any questions for me?

1) Intervention coherence (TFA) / Knowledge (TDF)

   a) Have you noticed any recent changes to the NeoTree? Any recent additions?

      [show diagnostic tool]

   b) Can you talk me through the diagnostic tool and what you think it hopes to achieve?

2) Knowledge & Skills (TDF)

   a) Overall, how easy or difficult to use the non-emergency diagnostic tool? What in particular is easy? What in particular is difficult?

   b) What would make the diagnostic tool easier to use?

3) Beliefs about consequences (TDF)

   a) How might having the diagnostic tool change practice, if at all?

      Prompt- how might it influence your decision making/actions?

   b) What are the benefits of having the diagnostic tool? Any downsides?

4) Burden (TFA)

   a) How does the new diagnostic tool affect your workload?

   Prompt: Do you have any extra tasks with the addition of the diagnostic tool? Does it require more effort/ time compared to when the NeoTree did not have this function?

5) Affective attitude (TFA) and Emotion (TDF)

   a) Do you like using the diagnostic tool? What in particular do you like or dislike?

      Optional Prompt: Any concerns or worries?

6) Social influences (TDF)

   a) To what extent do the views of others influence if and how you use the diagnostic tool? For example the opinions of other nurses in the unit, the NeoTree ambassador, doctors, the NeoTree project management team, parents/guardians?

   b) Has the diagnostic tool changed team working in the unit in any way?

   Prompt: communication, roles, responsibilities?

7) Opportunity costs (TFA)

   a) Is there anything that you feel you must give up in order to use the new diagnostic tool?

8) Ethicality (TFA) & Social/Professional role & identity (TDF)

   a) Do you feel the use of the NeoTree to diagnose and manage sepsis and HIE is safe? For you and for babies?

   b) How does NeoTree fit in with your ways of working? Do you feel it is your responsibility to use a tool like this? Who else might be responsible for using the diagnostic tool?

9) Self-efficacy (TFA) Beliefs about capabilities (TDF)

   a) How confident do you feel using the diagnostic tool?
   b) Is there anything that could help you become more confident?

10) Environmental context and resources (TDF)

   a) Do you have enough time to use the diagnostic tool?
   b) Do you have the necessary resources available to use the diagnostic tool?

11) Memory, attention, decision making (TDF)

   a) Do you use the diagnostic tool to guide your decision-making? Does it make things easier / more difficult? How so?
   b) Have you ever forgotten to use the diagnostic tool?

      If yes - in what kind of situations?
   c) Are there ever any instances when you decided to deviate from the 1) the recommended diagnosis 2) the recommended management plan. Talk me through this….
   d) Are there ever any instances where you decided not to use the diagnostic tool? If so, can you talk me through this…

12) Goals (TDF)

   a) How important is using the diagnostic tool for you? And why?
   b) Compared to other things you have to do, where does the diagnostic tool fit in terms of priority? What are the competing priorities?
   c) Do you have any targets/goals for practice? How does the diagnostic tool fit in with that if at all?

13) Perceived effectiveness (TFA) and optimism (TDF)

   a) How likely is the diagnostic tool to improve quality of newborn care?

14) Reinforcement (TDF)

   a) What positive experiences have you had with the diagnostic tool that would encourage you to keep using it? Are there any negative experiences that would discourage you?
   b) What incentives / rewards / pressures are there to use the diagnostic tool?

15) Intentions (TDF)

   a) Do you intend to use the diagnostic tool in your day-to-day work? Can you explain your reasons?

16) Behavioural regulation (TDF)

a) To what extent has using the diagnostic tool become habitual (i.e. something that you do routinely) in your day to day practice? Why/how so?
b) Have you ever encountered any problems using the diagnostic tool? What were these and how did you overcome these?
c) What can we do to improve the use of the diagnostic tool on the ward?

To summarise, the main points from our discussion are (facilitator to summarise key points). Is there anything else you'd like to add? Is there anything you'd like to ask me?

Thank you so much. We really appreciate the time you've taken to participate in this study.

Topic Guide 6: FGD - sustainability

Topic guide for NeoTree focus groups based on Theoretical Framework of Acceptability (TFA) and the Theoretical Domains Framework (TDF)

Target behaviours – Use of all functions of the NeoTree as part of routine care (without support of NeoTree team)

Timing of focus group: sustainability phase (months 16-21)

Participants: Healthcare Professionals

Version: 1.0

Introduction

Hello, thank you very much for taking the time to speak to me today. My name is ______ and I work for ______________. This discussion will probably between 1-2 hours. As a reminder, I am talking to you today, as I work for a study that aims to improve the care given to sick and vulnerable babies in hospitals.

Before we start, can I check whether you have:

- been told about the study
- had an information sheet
- signed a consent form
- agreed to audio recording of the discussion

At this point, do you have any questions about the purpose of the study, or the documents you've been given [e.g. PIS/ consent form]? Is there anything that isn't clear?

I just want to remind you that your participation in this discussion is entirely voluntary [i.e. it is your choice].

I am interested in your views about the implementation of the NeoTree at [health facility]. Your opinions as health care workers at the frontline of newborn care are very important to us. There are no right or wrong answers to these questions, just differing points of views. Please feel free to share your point of view even if it differs from what others have said. Keep in mind that we're just as interested in negative comments as positive comments

Please give everyone a chance to speak, and please do not share what we discuss today outside this group. Please say your ID number before you speak and do not say names during the discussion as we want to keep them anonymised.

If you want to take a break or stop at any point, please tell me. And if you wish to withdraw from the study you are completely free to do so at any point.

Before we start, do you have any questions for me?

1) Introductory questions

   NeoTree has been implemented on this unit/ward for X months now.

   a) How much, if at all, do you think NeoTree is still used in day-to-day practice at the moment?
   b) Has this changed over time (i.e. increased/ decreased)?

2) Knowledge & Skills (TDF)

   a) How easy or difficult is it to use the NeoTree? Is it more or less easy to use the NeoTree since the NeoTree team have left?
   b) Do you think any additional training, information or support is needed to continue to use NeoTree now that the research team are no longer present/ have left?

      If so, what would be needed?

3) Beliefs about consequences (TDF)

   a) Do you think there are any benefits to continuing to use the NeoTree in future?
   b) Do you think there are any drawbacks to continuing to use the NeoTree in future?
   c) Do the pros outweigh the cons?

4) Burden (TFA)

   a) Has your role changed at all since the team has left? How?
   b) How does the NeoTree affect your capacity to do your job? Does it help or hinder you to do your job?

5) Affective attitude (TFA) and Emotion (TDF)

   a) Do you like using NeoTree? What in particular do you like or dislike now that the team has left?
   b) Have your feelings about using the NeoTree changed since the team has left?

6) Social influences (TDF)

   a) Do you have any concerns or fears about using the NeoTree now that the team has left?
   b) Do the views of others influence if and how you use the NeoTree? For example the opinions of other nurses in the unit, the NeoTree ambassador, doctors, parents/guardians
   c) Since the team left, has the NeoTree changed team working in the unit? In what way? (e.g. communication, roles. Responsibilities?)

   Optional prompt: Any disagreements/ conflict?

7) Opportunity costs (TFA)

a) Is there anything that you feel you must give up in order to use the NeoTree? Do you feel that you have given up anything new since the team has left?
b) Given the choice, would you prefer the team to return? Can you explain your reasons?

8) Ethicality (TFA) Social/Professional role & identity (TDF)

a) Do you feel it is safe to continue to use NeoTree? For you and for babies? Do you feel that the NeoTree is more/ less safe since the team has left?
b) Do you feel using the NeoTree is fair now that the team has left?
c) Now that the team has left, how does NeoTree fit in with your ways of working?
d) Going forward, do you feel it is your responsibility to continue to use the NeoTree? Who else might be responsible to ensure the sustained use of the NeoTree?

9) Self-efficacy (TFA) Beliefs about capabilities (TDF)

a) Since the team has left, are you more or less confident to use the NeoTree?
b) Which aspects of the NeoTree are you confident about?
c) Which aspects are you less confident about? What could help to improve your confidence going forward?

10) Environmental context and resources (TDF)

a) Now that the team has left, do you have enough time to use the NeoTree?
b) Do you have the necessary resources to continue to use the NeoTree in future?
What else would be needed?

11) Memory, attention, decision making (TDF)

a) Have you ever forgotten to use the NeoTree since the team has left? Talk me through this
b) Since the team left, have there been instances when you decided not to use the NeoTree? Talk me through this, what did you do instead?
c) Since the team left, have there been instances when you decided not to follow the guidance of the NeoTree? Talk me through this, what did you do instead?

12) Goals (TDF)

a) How important is using NeoTree for you? And why? Has this changed since the team left?
b) Compared to other things you have to do, where using NeoTree fit in in terms of a priority? What are the competing priorities?
c) Do you have any targets/goals for future practice? How does NeoTree fit in with that if at all?

13) Perceived effectiveness (TFA) + Optimism (TDF)

a) Going forward, how likely is the NeoTree to improve quality of newborn care?

14) Reinforcement (TDF)

a) What positive experiences have you had with the NeoTree that would encourage you to use it in the long term? Are there any negative experiences that would discourage you from using NeoTree in the long term?

b) Are there any incentives / rewards / pressures to continue to use NeoTree in future?

15) Intentions (TDF)

a) To what extent do you intend to use NeoTree in the future? Why/ why not?

16) Behavioural regulation (TDF)

a) Have you encountered any problems using NeoTree since the team has left? How did you overcome these?

b) What needs to be done to support use of NeoTree longer term? (what, by whom, where, when?)

To summarise, the main points from our discussion are (facilitator to summarise key points). Is there anything else you'd like to add? Is there anything you'd like to ask me?

Thank you so much. We really appreciate the time you've taken to participate in this study.
