# Supplementary material for: Protocol for an intervention development and pilot implementation evaluation study of an e-health solution to improve newborn care quality and survival in two low-resource settings, Malawi and Zimbabwe: Neotree
Source: BMJ Open. 2022 Jul 5;12(7):e056605. doi: 10.1136/bmjopen-2021-056605 (PMC9258512; doi:10.1136/bmjopen-2021-056605)
Supplement: Supplementary data [file bmjopen-2021-056605supp002.pdf]

Supplementary File 2

Draft topic guides for semi-structured interviews (SSIs) with senior managers and clinical staff

SSI guide 1 – Baseline

Interview guide based on Theoretical Framework of Acceptability/ Theoretical Domains Framework (TDF).

Target behaviour: intention to introduce and implement the NeoTree

Timing of interviews: pre- implementation phase (months 5-6)

Participants: senior managers/ administrative staff/ clinicians

Version 1.0

Introduction

Hello, thank you very much for taking the time to speak to me today. My name is ______ and I work for _____________. This interview will probably last around 1 hour. As a reminder, I am talking to you today, as I work for a study that aims to improve the care given to sick and vulnerable babies in hospitals.

Before we start, can I check whether you have:

- been told about the study
- had an information sheet
- signed a consent form
- agreed to audio recording of the discussion

At this point, do you have any questions about the purpose of the study, or the documents you've been given [e.g. PIS/ consent form]? Is there anything that isn't clear?

I want to reassure you that I work for a research organisation [name] and not [name of health facility]. Anything you tell me today is confidential and will not be shared with your colleagues. All personal and identifying information (such as your name/names of others) mentioned will be removed and replaced with a code.

I just want to remind you that your participation in this interview is entirely voluntary [i.e. it is your choice]. If you do not want to answer a question you can just say 'pass' and we will move on to the next question.

I am interested in your views about the introduction of the NeoTree at [health facility]. There are no right or wrong answers to these questions; I am just interested in your views so please answer honestly.

If you want to take a break or stop at any point, please tell me. And if you wish to withdraw from the study you are completely free to do so at any point.

Before we start, do you have any questions for me?

1) Warm up questions

What is your position at this hospital? How long have you worked here?

2) Introduction/ demonstration of the NeoTree

The NeoTree is a tablet-based clinical app, NeoTree, which combines evidence-based clinical guidelines with real-time data collection and education about newborn clinical needs. The NeoTree aims to improve quality of care and newborn survival through:

A) data-capture,

B) emergency decision-support

C) non-emergency clinical and management decision-support

D) feedback of data to dashboards and national aggregate data systems.

Health care workers (HCWs) use the app at the bedside to admit and discharge each newborn patient. As they complete the admission, they receive prompts to respond appropriately to the data they have entered and manage patients according to evidence-based guidelines. We also plan to link to national data systems (Electronic Medical Records).

Do you have any questions?

1) Knowledge & Skills (TDF)

a) How easy or difficult do you think it would be to introduce the NeoTree at [HEALTH FACILITY]?

Prompt: What in particular would be easy? What in particular would be difficult?

b) Do you think the staff would require any additional skills or training to use/ manage NeoTree?

2) Beliefs about consequences (TDF)

a) What do you perceive to be the benefits/positives of introducing the NeoTree at [HEALTH FACILITY]?

Prompt: for: you, other colleagues/roles and for patients & families?

b) Would there be any drawbacks?

Prompt: for you, other colleagues/roles and for patients and families?

c) Overall, do you think the pros would outweigh the cons?

3) Burden (TFA + Social Professional Role/Identity (TDF)

a) Do you think using the NeoTree would create extra work for you, for other staff? In what way?

b) Do you feel it is your responsibility oversee the introduction of the NeoTree? Who else might be responsible?

4) Affective attitude (TFA) and Emotion (TDF)

   a) In general, how would you feel about the introduction and use of the NeoTree at [health facility]?

      Positive emotions: pride, confidence, satisfaction, reassurance
      Negative emotions; guilt, worry, concern, pressure

   b) Do you have any worries or concerns about introducing digital aids such as the NeoTree into clinical care at this hospital?

5) Social influences (TDF)

   a) Do you know of other hospitals using NeoTree or digital innovations? To what extent has this impacted on your decision to introduce the NeoTree in this facility?

6) Perceived Opportunity costs (TFA)

   a) Do you think introducing the NeoTree would be a good use of resources?

7) Perceived Self-efficacy (TFA) Beliefs about capabilities (TDF)

   a) To what extent do you feel confident staff in this facility could implement Neotree? What else would be needed?

8) Ethicality (TFA)

   a) Do you feel the NeoTree would be safe? For you and for babies?
   b) Do you feel introducing the NeoTree would be fair for you? For other staff?

9) Environmental context and resources (TDF)

   a) Do you think you would have sufficient resources to implement the NeoTree?

10) Goals (TDF)

   a) Compared to other things you have to do, where does introducing the NeoTree fit in in terms of a priority? What would be the competing priorities?
   b) Do you have any targets/goals for practice? How would the NeoTree fit in with that if at all?

11) Perceived effectiveness (TFA) + optimism (TDF)

In your view, how likely is the NeoTree to improve quality of newborn care at [HEALTH FACILITY]?

12) Intentions (TDF)

   a) Would you like this hospital to use the NeoTree? Can you explain your reasons why/why not?

13) Barriers/ facilitators (general)

   a) What do you think might be some of the barriers to introducing the NeoTree at [HEALTH FACILITY]?

b) Is there anything that might make it easier to implement the NeoTree at [HEALTH FACILITY]?

14) Closing questions/ remarks

a) Now that we've completed the interview, is there anything you'd like to ask me or do you have anything further to add?

Thank you so much, we really appreciate the time you've taken to participate in this study.

SSI guide 2 - Implementation

Interview guide based on Theoretical Framework of Acceptability/ Theoretical Domains Framework (TDF).

Interview guide to explore (A) perceptions and understanding of quality newborn care and (B) acceptability of Neotree/ barriers and facilitators to implementation

Timing of interviews: implementation phase (months 6-16)

Participants: senior managers/ administrative staff/ clinicians

Version 1.0

Introduction

Hello, thank you very much for taking the time to speak to me today. My name is ______ and I work for _____________. This interview will probably last around 1 hour. As a reminder, I am talking to you today, as I work for a study that aims to improve the care given to sick and vulnerable babies in hospitals.

Before we start, can I check whether you have:

- been told about the study
- had an information sheet
- signed a consent form
- agreed to audio recording of the discussion

At this point, do you have any questions about the purpose of the study, or the documents you've been given [e.g. PIS/ consent form]? Is there anything that isn't clear?

I want to reassure you that I work for a research organisation [name] and not [name of health facility]. Anything you tell me today is confidential and will not be shared with your colleagues. All personal and identifying information (such as your name/names of others) mentioned will be removed and replaced with a code.

I just want to remind you that your participation in this interview is entirely voluntary [i.e. it is your choice]. If you do not want to answer a question you can just say 'pass' and we will move on to the next question.

I am interested in your views about the implementation of the NeoTree at [health facility]. There are no right or wrong answers to these questions; I am just interested in your views so please answer honestly.

If you want to take a break or stop at any point, please tell me. And if you wish to withdraw from the study you are completely free to do so at any point.

Before we start, do you have any questions for me?

1) Warm up questions

What is your position at this hospital? How long have you worked here?

2) Quality of newborn care

a) What does high quality newborn care mean to you?

Prompt- What do you understand by high quality care?

b) Who do you see as the providers of care in the neonatal unit?

Prompt - nurses, students, doctors, mother, other family members

Now I'd like to ask you some questions about the NeoTree. I'm really interested to understand your views and feelings with regard to the NeoTree.

3) Intervention coherence (TFA)

a) Can you talk me through what your understanding of NeoTree is? What it involves, its purpose, who it is intended to be used by, when, where etc?

4) Barriers/ enablers (general)

a) What have been the barriers to implementing NeoTree in this hospital?

b) What would help make it easier to implement the NeoTree here?

5) Experienced affective attitude (TFA) and Emotion (TDF)

a) In general, how do you feel about the introduction and use of the NeoTree at [health facility]?

Positive emotions: pride, confidence, satisfaction, reassurance
Negative emotions; guilt, worry, concern, pressure

b) Specifically, how useful do you find the different functionalities of the NeoTree:

- Data capture (i.e. admission, discharge and lab data);
- Diagnostic support (emergency and non-emergency);
- Data dashboards and quality improvement goals
- Data linkage to local and national databases

c) Do you have any worries or concerns about introducing digital aids such as the NeoTree into clinical care at this hospital?

6) Experienced burden (TFA)

a) Has the NeoTree required any extra time/ work/ resources? For you personally, from your staff?

7) Ethicality (TFA)

a) Do you think that the NeoTree is safe? For staff, for patients? Any concerns/ worries?

b) Has the NeoTree led to any changes in team working and communication? Have these been positive or negative? Any divisions between staff or conflicts?

8) Experienced opportunity costs (TFA)

   a) Do you think the NeoTree is a good use of resources? Why/why not?

9) Experienced effectiveness (TFA)

   a) At this stage of implementation to what extent do you feel the NeoTree has improved quality of newborn care? What in particular has helped improve quality of care?

10) Experienced self-efficacy (TFA)

   a) What is your role in the implementation of the NeoTree?
   b) How confident do you feel in carrying out this role? Which aspect of your role do you like/ and which do you like less?
   c) To what extent do you feel confident staff in this facility can implement Neotree? What else would be needed?

11) Social influences (TDF)

   a) Do you know of other hospitals using NeoTree or digital innovations? Did that impact on your decision to implement NeoTree in this facility at all?

12) Intentions (TDF)

   a) To what extent do you plan to use the NeoTree in the future? Can you explain your reasons?

13) Behavioural regulation (TDF)

   a) Have you ever encountered any problems in implementing/introducing NeoTree to this facility? If so, can you talk me through these? How did you overcome these?
   b) Is there anything you think we can do to improve the implementation of the NeoTree in this hospital?

14) Closing questions/ remarks

   a) In your view, what 5 things are needed to improve quality of neonatal care in this hospital?
   b) Now that we've completed the interview, is there anything you'd like to ask me or do you have anything further to add?

Thank you so much, we really appreciate the time you've taken to participate in this study.

SSI Guide 3 - Sustainability

Interview guide to explore acceptability of Neotree/ barriers and facilitators to sustainability. Based on Theoretical Framework of Acceptability (TFA)/ Theoretical Domains Framework (TDF)

Timing: Sustainability phase (months 16-21)

Participants: senior managers/ administrative staff/ clinicians

Version: 1.0

Introduction

Hello, thank you very much for taking the time to speak to me today. My name is ______ and I work for _____________. This interview will probably last around 1 hour. As a reminder, I am talking to you today, as I work for a study that aims to improve the care given to sick and vulnerable babies in hospitals.

Before we start, can I check whether you have:

- been told about the study
- had an information sheet
- signed a consent form
- agreed to audio recording of the discussion

At this point, do you have any questions about the purpose of the study, or the documents you've been given [e.g. PIS/ consent form]? Is there anything that isn't clear?

I want to reassure you that I work for a research organisation [name] and not [name of health facility]. Anything you tell me today is confidential and will not be shared with your colleagues. All personal and identifying information (such as your name/names of others) mentioned will be removed and replaced with a code.

I just want to remind you that your participation in this interview is entirely voluntary [i.e. it is your choice]. If you do not want to answer a question you can just say 'pass' and we will move on to the next question.

I am interested in your views about the implementation of the NeoTree at [health facility]. There are no right or wrong answers to these questions; I am just interested in your views so please answer honestly.

If you want to take a break or stop at any point, please tell me. And if you wish to withdraw from the study you are completely free to do so at any point.

Before we start, do you have any questions for me?

1) Warm up questions

How are you? How have you been since we last spoke? Any change in terms of your role and responsibilities at the hospital?

The last time we spoke, I asked you a series of questions about your views and experiences of using the NeoTree. Since then, the NeoTree project managers and developers have left and no longer provide support on site.

I'd like to ask you some questions about how things have been since they left.

2) Intervention coherence (TFA)

a) To what extent do you think NeoTree is currently used/ has this changed over time/ why / how so
b) Has there been anything that has made it harder to implement NeoTree and sustain its use?
c) Anything that has made it easier?

3) Experienced affective attitude (TFA) & Emotions (TDF)

a) Have your feelings about the NeoTree changed since we last spoke [prompt previous answer].
b) Do you have any concerns or fears about implementing the NeoTree in this hospital now that the team has left

4) Experienced Burden (TFA)

Since we last spoke [prompt previous answers] do you think the NeoTree:

a) Has generated any extra work in terms of supervision or staffing?
b) Has required any extra time. Extra resources? from you personally, from your staff?
c) Since we last spoke, has the NeoTree diverted staff/ equipment/ funds away from other aspects of patient care? If so, what impact has this had on you personally, on other staff and on patients?

5) Ethicality (TFA)

a) Now that the team has left, do you think that the NeoTree is safe? For staff, for patients? Any changes/ concerns since we last spoke?
b) Since the last interview, do you think the NeoTree has led to any changes in team working and communication? Have these been positive or negative?

6) Experienced opportunity costs (TFA)

a) Do you think the NeoTree is a good use of resources? Why/why not? Has your opinion changed since we last spoke?

7) Experienced effectiveness (TFA)

   a) Since we last spoke, do you feel the NeoTree has led to any further improvements in quality of care? What in particular has helped/ hindered quality of care?

8) Experienced self-efficacy (TFA)

   a) Last time you told me that your role in the NeoTree is [insert from previous interview]. Has this changed since our last interview?
   b) How confident do you feel in carrying out this role? Do you feel more or less confident since our last interview? Why?
   c) To what extent do you feel confident staff in this facility can sustain NeoTree in the future? What else would be needed?

9) Intentions (TDF)

   a) To what extent do you intend to sustain the NeoTree at this facility? Can you explain your reasons to me?

10) Barriers and enablers to sustainability (general)

   a) What would it take to continue to use NeoTree going forward? What might get in the way of sustainability?
   b) Is sustaining NeoTree a priority given other demands at this hospital?
   c) What could help support sustainability longer term?

11) Closing questions/ remarks

   a) Now that we've completed the interview, is there anything you'd like to ask me or do you have anything further to add?

Thank you so much, we really appreciate the time you've taken to participate in this study.
