# Supplementary material for: Protocol for an intervention development and pilot implementation evaluation study of an e-health solution to improve newborn care quality and survival in two low-resource settings, Malawi and Zimbabwe: Neotree
Source: BMJ Open. 2022 Jul 5;12(7):e056605. doi: 10.1136/bmjopen-2021-056605 (PMC9258512; doi:10.1136/bmjopen-2021-056605)
Supplement: Supplementary data [file bmjopen-2021-056605supp003.pdf]

Supplementary File 3:

Parent/ Carer Interview Guide to explore (A) perceptions and understanding of quality newborn care and (B) acceptability of Neotree

Timing of interviews: implementation phase (months 6-16)

Version 1.0

Introduction

Hello, thank you very much for taking the time to speak to me today. My name is ______ [name of researcher] and I work for _____________. This interview will probably last around 1 hour. As a reminder, I am talking to you today, as I work for a study that aims to improve the care given to sick and vulnerable babies in hospitals.

Before we start, can I check whether you have:

- been told about the study
- had an information sheet
- signed a consent form
- agreed to audio recording of the discussion

At this point, do you have any questions about the purpose of the study, or the documents you've been given [e.g. PIS/ consent form]? Is there anything that isn't clear?

I want to reassure you that I work for a research organisation [name] and not [name of health facility]. Anything you tell me today will not affect the care of your baby or your family. All personal and identifying information (such as your name/names of others) mentioned will be removed and replaced with a code.

I just want to remind you that your participation in this interview is entirely voluntary [i.e. it is your choice]. If you do not want to answer a question you can just say 'pass' and we will move on to the next question.

I am interested in your views about the care of sick newborns, and your experiences of receiving care in this hospital. There are no right or wrong answers to these questions; I am just interested in your views so please answer honestly.

If you want to take a break or stop at any point, please tell me. And if you wish to withdraw from the study you are completely free to do so at any point.

As a reminder, your answers to these questions will not affect the care you or your family will receive at all. Do you have any questions for me?

1) Warm up questions

How are you feeling today? How long have you/ your baby/ family member been in the hospital? Where have you travelled from?

2) Quality of newborn care

a) What does high quality newborn care mean to you?

Prompt: What do you understand by high quality newborn care?

b) In your view, who provides most of the care in the neonatal unit?

Prompt: nurses, students, doctors, mothers, family members etc..

Acceptability of current care and use of digital aids

Now I'd like to ask you some questions about the NeoTree digital health aid. You probably noticed that when the HCW asked you questions about you/ your baby/ family member she/ he was using a tablet with the NeoTree app.

The NeoTree app uses the information that you give to the healthcare worker to work out what is likely to be wrong with the baby and how to best treat and manage the baby. Whilst the baby is on the newborn care unit it will also provide an electronic linkage to any tests that are done to check for infection. As well as providing information on what might be wrong with the baby, the NeoTree app also gives the healthcare workers education and tips around how to care for the newborn. The NeoTree also stores the clinical information for all of the babies and then feeds back to the healthcare workers and the hospital on things like how many babies were admitted each month and what was wrong with them so that they can best plan the services needed.

3) Experienced affective attitude (TFA) and Emotions (TDF)

Gathering and storing health information

a) How did you feel about answering questions about you and about your baby's health?

   - Prompts to illicit feelings/ emotions:
     Positive emotions: pride, confidence, satisfaction, reassurance
     Negative emotions; guilt, worry, concern, pressure

b) How did you feel about the HCW entering your answers into the tablet [show visual aid]? Did you have any concerns about this at all?

c) Once your answers were entered into the tablet, your answers were stored electronically. How did you feel about the HCW storing (keeping) your answers on the tablet [show visual aid]?

Diagnosis and management of newborns

- How do you feel about the use of the NeoTree to support HCWs to diagnose and treat sick babies? [show example] Any concerns or worries?

4) Experienced burden (TFA)

   a) How easy or difficult was it to answer the HCW's questions, when your baby was admitted using the NeoTree?
   b) Were there any questions that were difficult or complicated to answer?
   c) Do you think the NeoTree helped the HCWs provide care more efficiently to your baby? Did it get in the way at all? How so?

5) Experienced effectiveness (TFA)

   a) Do you think the NeoTree was helpful for HCWs to provide care to babies? How so?
   b) Do you feel the NeoTree has helped in the care of your baby? In what way?
   c) Have you experienced any challenges while your baby has been in the unit?

6) Ethicality (TFA)

   a) Did you feel fairly treated while they were using the NeoTree? Would you change any aspect of the way you were treated while they were using the NeoTree?
   b) Do you think the NeoTree distracted HCWs from caring for your baby?
   c) Do you have any safety concerns about the use of the NeoTree in the care of your baby?

7) Intervention coherence (TFA)

   a) How clear was the information you were given about your baby's care [treatment/procedures]? Was anything not clear/ difficult to understand?
   b) How do you think the data/information about you and your baby's health is used? What do you think happens to this information?
   c) Do you think the NeoTree can help improve quality of newborn care? In what way?

8) Experienced opportunity costs (TFA)

   a) Do you think there are any risks to you or your baby when the HCW uses NeoTree?
   b) If you had the choice, would you prefer HCWs to record information about you and your baby on paper (i.e. not use the NeoTree)? If so why?
   c) If you had the choice, would you prefer HCWs diagnose and manage your baby without the help of the NeoTree? If so, why?

9) Experienced self-efficacy (TFA)

   a) How confident did you feel speaking to healthcare workers about the care of your baby?
   b) How involved did you feel involved in the care of you baby? Clinical decisions etc?

10) Closing questions/ remarks

a) If you could change one thing about the care and treatment you've received at the unit, what would you change?

- Now that we've completed the interview, is there anything you'd like to ask me?

Thank you so much, we really appreciate the time you've taken to participate in this study.
