# Supplementary material for: Protocol for an intervention development and pilot implementation evaluation study of an e-health solution to improve newborn care quality and survival in two low-resource settings, Malawi and Zimbabwe: Neotree
Source: BMJ Open. 2022 Jul 5;12(7):e056605. doi: 10.1136/bmjopen-2021-056605 (PMC9258512; doi:10.1136/bmjopen-2021-056605)
Supplement: Supplementary data [file bmjopen-2021-056605supp006.pdf]

Supplementary File 6

Data Management Procedures

When a baby is admitted, an admission form is completed on the NeoTree tablet app and an ID for the neonate is generated. The NeoTree ID generated is stored in the nurses' routine admission book along with the mother's name, acting as the data spine. Personal identifiable information (PII) is collected as part of the electronic admission form (incl. mother's name, baby's name, baby's date of birth). All data (including PII) are stored temporarily in an encrypted format on the tablet and hidden from view: only members of the research/clinical team can retrieve these data from the tablet by supplying a valid password. The identifiable NeoTree forms will be printed and inserted into clinical paper records to replace usual hand-written forms. All PII fields (e.g. mother's name, baby's name are not exported from the tablet into the NeoTree backend. PII fields are only stored in the tablet in an encrypted format.

Any other forms (e.g. discharge forms, laboratory results forms) submitted on the app are recorded against the neonates "NeoTree ID". In addition, some PII may also be stored (e.g. the mother's name) on the tablet, although this will be password protected as above, and records will be routinely wiped from the tablet as an extra safeguard.

In Zimbabwe, non-PII data from all forms are saved to a secure database at a Zimbabwean data centre: the "Clinical database". This database will be locally owned, stored and managed. The NeoTree team will have access to the database in order to ensure that the process for moving data from tablets to database is successful, and to enable a copy of the anonymized data for research purposes. (See "Anonymised research database" below.) Data moved from the tablets to the secure database will be encrypted in transit. Data are encrypted at rest in the Clinical database, where they are password protected. The only way to tie individual records in the database back to the neonate they describe is by matching the NeoTreeID with the PII, based on the paper print out stored in the patient's notes and the nurses' routine admission book. A regular process is in place to wipe data from the tablets, so encrypted identifiable data are stored for only a brief period of time (e.g. if a second copy of the print out is needed for clinical reasons). Aggregate statistics are computed on the data in the secure database, and served back to staff in the hospital via dashboards. A regular process will be put in place to copy anonymized data from the Clinical database into the "Anonymised research database", curated by the NeoTree research team and held on a UCL server. Data will be encrypted in transit and at rest. The entire approach to data security is twofold: (i) minimize the chance of data leakage, by ensuring that data is encrypted in transit and at rest, and by

minimizing the number of places the data is stored and (ii) pseudonomizing the data at the point of collection, so that even in the event of a breach, PII would not be leaked.

In Malawi, the process is identical except that the “clinical database” is stored on a secure virtual machine in an encrypted format using Amazon Webserver (AWS).
