# Supplementary material for: Protocol for an intervention development and pilot implementation evaluation study of an e-health solution to improve newborn care quality and survival in two low-resource settings, Malawi and Zimbabwe: Neotree
Source: BMJ Open. 2022 Jul 5;12(7):e056605. doi: 10.1136/bmjopen-2021-056605 (PMC9258512; doi:10.1136/bmjopen-2021-056605)
Supplement: Supplementary data [file bmjopen-2021-056605supp007.pdf]

Supplementary File 7

Summary of implementation and research plan adaptation in response to COVID-19

1. Data collection.

In 2020 we submitted ethics amendments to allow for the option of remote data collection via secure platforms such as Microsoft Teams, if face-to-face data generation was deemed is not feasible due to COVID-19 restrictions. In August- December 2020, researchers conducted 17 semi-structured interviews with healthcare professionals at Chinhoyi Provincial Hospital, Zimbabwe. Most were conducted via remote methods (Microsoft Teams) due to travel restrictions which prevented on-site visits. The remainder of data collection is likely to be generated in face-to-face settings, with enhanced safety measures in line with hospital protocols, such as physical distancing, increased ventilation and mask wearing.

Routine admission and discharge data collection via the NeoTree platform has continued uninterrupted.

2. Implementation

Roll out of some functionalities was delayed for example the roll-out of the data dashboards in Zimbabwe. Clinical processes have inevitably adapted, for example reduced rotas of staff and suspension of face-to-face morbidity and mortality meetings during periods of heightened case numbers.

Nevertheless the NeoTree has proved robust in the face of these challenges. We have been highly responsive and adaptive to the pandemic, ensuring the safety of staff (for example remote support to clinical teams) while ensuring the NeoTree system has maintained function, data capture, analysis and emergency decision support for HCWs.

After media reports of increased stillbirths at Sally Mugabe Central Hosspital (SMCH) associated with COVID-19 pandemic reductions in hospital access, the NeoTree was adapted in one week at SMH to capture maternal and stillbirth outcomes. A similar process has been approved at Kamuzu Central Hospital, Malawi.
