# Supplementary material for: Protocol for an intervention development and pilot implementation evaluation study of an e-health solution to improve newborn care quality and survival in two low-resource settings, Malawi and Zimbabwe: Neotree
Source: BMJ Open. 2022 Jul 5;12(7):e056605. doi: 10.1136/bmjopen-2021-056605 (PMC9258512; doi:10.1136/bmjopen-2021-056605)
Supplement: Author's manuscript [file bmjopen-2021-056605.draft_revisions.pdf]

BMJ Open

BMJ Open is committed to open peer review. As part of this commitment we make the peer review history of every article we publish publicly available.

When an article is published we post the peer reviewers' comments and the authors' responses online. We also post the versions of the paper that were used during peer review. These are the versions that the peer review comments apply to.

The versions of the paper that follow are the versions that were submitted during the peer review process. They are not the versions of record or the final published versions. They should not be cited or distributed as the published version of this manuscript.

BMJ Open is an open access journal and the full, final, typeset and author-corrected version of record of the manuscript is available on our site with no access controls, subscription charges or pay-per-view fees (http://bmjopen.bmj.com).

If you have any questions on BMJ Open's open peer review process please email info.bmjopen@bmj.com

BMJ Open

Protocol for an intervention development and pilot implementation evaluation study of an e-health solution to improve newborn care quality and survival in two low resource settings, Malawi and Zimbabwe: the NeoTree.

Journal:	*BMJ Open*
Manuscript ID	bmjopen-2021-056605
Article Type:	Protocol
Date Submitted by the Author:	19-Aug-2021

Complete List of Authors:	Wilson, Emma; UCL Great Ormond Street Institute of Child Health Population Policy and Practice, Population, Policy and Practice Research and Teaching Department<br>Gannon, Hannah; UCL Great Ormond Street Institute of Child Health Population Policy and Practice, Population, Policy and Practice Research and Teaching Department<br>Chimhini, Gwen; University of Zimbabwe College of Health Sciences, Unit of Child and Adolescent Health<br>Fitzgerald, Felicity; University College London, Infection Immunity and Inflammation, Great Ormond Street Institute of Child Health<br>Khan, Nushrat; UCL Great Ormond Street Institute of Child Health Population Policy and Practice<br>Lorencatto, Fabiana; University College London Centre for Behaviour Change<br>Kesler, Erin; The Children's Hospital of Philadelphia<br>Nkhoma, Deliwe; Parent and Child Health Initiative<br>Chiyaka, Tarisai; Biomedical Research and Training Institute<br>Haghparast-Bidgoli, Hassan; University College London Institute for Global Health<br>Lakhanpaul, Monica; UCL Great Ormond Street Institute of Child Health Population Policy and Practice<br>Cortina Borja, Mario; UCL Great Ormond Street Institute of Child Health Population Policy and Practice<br>Stevenson, Alexander; Mbuya Nehanda Maternity Hospital<br>Crehan, Caroline; University College London, Institute of Child Health UCL-GOSH<br>Sassoon, Yali; Snowplow Analytics<br>Hull-Bailey, Tim; University College London, Population<br>Curtis, Kristina; University College London Centre for Behaviour Change<br>Chiume, Msandeni; Kamuzu Central Hospital, Department of Paediatrics<br>Chimhuya, Simbarashe; University of Zimbabwe, Unit of Child and Adolescent Health, Faculty of Medicine and Health Sciences,<br>Heys, Michelle; UCL Great Ormond Street Institute of Child Health Population Policy and Practice

Keywords:	NEONATOLOGY, HEALTH SERVICES ADMINISTRATION & MANAGEMENT, PUBLIC HEALTH, EPIDEMIOLOGY

SCHOLARONE™
Manuscripts

Note from the Editors: Instructions for reviewers of study protocols

Since launching in 2011, BMJ Open has published study protocols for planned or ongoing research studies. If data collection is complete, we will not consider the manuscript.

Publishing study protocols enables researchers and funding bodies to stay up to date in their fields by providing exposure to research activity that may not otherwise be widely publicised. This can help prevent unnecessary duplication of work and will hopefully enable collaboration. Publishing protocols in full also makes available more information than is currently required by trial registries and increases transparency, making it easier for others (editors, reviewers and readers) to see and understand any deviations from the protocol that occur during the conduct of the study.

The scientific integrity and the credibility of the study data depend substantially on the study design and methodology, which is why the study protocol requires a thorough peer-review.

BMJ Open will consider for publication protocols for any study design, including observational studies and systematic reviews.

Some things to keep in mind when reviewing the study protocol:

- Protocol papers should report planned or ongoing studies. The dates of the study should be included in the manuscript.
- Unfortunately we are unable to customize the reviewer report form for study protocols. As such, some of the items (i.e., those pertaining to results) on the form should be scores as Not Applicable (N/A).
- While some baseline data can be presented, there should be no results or conclusions present in the study protocol.
- For studies that are ongoing, it is generally the case that very few changes can be made to the methodology. As such, requests for revisions are generally clarifications for the rationale or details relating to the methods. If there is a major flaw in the study that would prevent a sound interpretation of the data, we would expect the study protocol to be rejected.

For peer review only

TITLE: Protocol for an intervention development and pilot implementation evaluation study of an e-health solution to improve newborn care quality and survival in two low resource settings, Malawi and Zimbabwe: the NeoTree.

Emma Wilson
Population, Policy and Practice Research and Teaching Department, University College London Great Ormond Street Institute of Child Health, 30 Guilford Street, London WC1N 1EH, UK, e.k.wilson@ucl.ac.uk

Hannah Gannon
Population, Policy and Practice Research and Teaching Department, University College London Great Ormond Street Institute of Child Health, 30 Guilford Street, London WC1N 1EH, UK, h.gannon@ucl.ac.uk

Gwendoline Chimhini
Unit of Child and Adolescent Health, Faculty of Medicine and Health Science, University of Zimbabwe, P.O. Box A178, Avondale, Harare, Zimbabwe, gwenchimhini@gmail.com.

Felicity Fitzgerald
Infection, Immunity and Inflammation, University College London Great Ormond Street Institute of Child Health, 30 Guilford Street, London WC1N 1EH, UK, felicity.fitzgerald@ucl.ac.uk

Nushrat Khan
Population, Policy and Practice Research and Teaching Department, University College London Great Ormond Street Institute of Child Health, 30 Guilford Street, London WC1N 1EH, UK, nushrat.khan@ucl.ac.uk

Fabiana Lorencatto
Centre for Behaviour Change, University College London, London, UK, f.lorencatto@ucl.ac.uk

Erin Kesler
Children's Hospital of Philadelphia, 3401 Civic Center Blvd, Philadelphia, USA, keslerem@gmail.com

Deliwe Nkhoma
Parent and Child Health Initiative (PACHI), Lilongwe, Malawi, dbnkhoma@gmail.com

Tarisai Chiyaka
Biomedical Research and Training Institute (BRTI), 10 Seagrave Road Avondale, Harare, Zimbabwe, tariemc@gmail.com

Hassan Haghparast-Bidgoli
Institute for Global Health, University College London, 30 Guilford Street, London WC1N 1EH, UK, h.haghparast-bidgoli@ucl.ac.uk

Monica Lakhanpaul
Population, Policy and Practice Research and Teaching Department, University College London Great Ormond Street Institute of Child Health, 30 Guilford Street, London WC1N 1EH, UK, m.lakanpaul@ucl.ac.uk

Mario Cortina-Borja
Population, Policy and Practice Research and Teaching Department, University College London Great

For peer review only

Ormond Street Institute of Child Health, 30 Guilford Street, London WC1N 1EH, UK, m.cortina@ucl.ac.uk

Alexander G. Stevenson,
Mbuya Nehanda Maternity Hospital, Harare, Zimbabwe. Department of Paediatrics, University of Zimbabwe, Harare, Zimbabwe doctoralexstevenson@gmail.com

Caroline Crehan
Population, Policy and Practice, University College London Great Ormond Street Institute of Child Health, 30 Guilford Street, London WC1N 1EH, UK, c.crehan@ucl.ac.uk

Yali Sassoon
Snowplow Analytics, Floor 6, 17 Bevis Marks, London, EC3A 7LN, yali@snowplowanalytics.com

Tim Hull-Bailey
Population, Policy and Practice Research and Teaching Department, University College London Great Ormond Street Institute of Child Health, 30 Guilford Street, London WC1N 1EH, UK, t.hull-bailey@ucl.ac.uk

Kristina Curtis
Centre for Behaviour Change, University College London, UK, k.curtis@ucl.ac.uk

Msandeni Chiume
Department of Paediatrics, Kamuzu Central Hospital, Lilongwe, Malawi, msandeni@gmail.com

Simbarashe Chimhuya
Child and Adolescent Health Unit, Faculty of Medicine and Health Sciences, University of Zimbabwe, Harare, Zimbabwe, chimhuya.simbarashe@gmail.com

Michelle Heys*
Population, Policy and Practice Research and Teaching Department, University College London Great Ormond Street Institute of Child Health, 30 Guilford Street, London WC1N 1EH, UK, m.heys@ucl.ac.uk

*Corresponding author

For peer review only - http://bmjopen.bmj.com/site/about/guidelines.xhtml

ABSTRACT

Introduction

Every year 2.4 million deaths occur worldwide in babies younger than 28 days. Approximately 70% of these deaths occur in low resource settings because of failure to implement evidence-based interventions. Digital health technologies may offer an implementation solution. Since 2014 we have worked in Bangladesh, Malawi, Zimbabwe, and the UK to develop and pilot the NeoTree: an android app with accompanying data visualisation, linkage and export. Its low-cost hardware and state-of-the-art software are used to improve bedside postnatal care and to provide insights into population health trends, to impact wider policy and practice.

Methods and analysis

This is a mixed methods (i) intervention co-development and optimisation and (ii) pilot implementation evaluation (including economic evaluation) study. The NeoTree will be implemented in two hospitals in Zimbabwe, and one in Malawi. Over the two-year study period clinical and demographic newborn data will be collected via the NeoTree, in addition to behavioural science informed qualitative and quantitative implementation evaluation and measures of cost, newborn care quality and usability. Six months of comparative newborn outcome data and cost data will be collected from two hospitals receiving usual care. NeoTree clinical decision support algorithms will be optimised according to best available evidence and clinical validation studies.

Ethics and dissemination

This is a Wellcome Trust funded project (215742_Z_19_Z). Research ethics approvals have been obtained: Malawi College of Medicine Research and Ethics Committee (P.01/20/2909; P.02/19/2613); UCL (17123/001, 6681/001, 5019/004); Medical Research Council Zimbabwe (MRCZ/A/2570), BRTI and JREC institutional review boards (AP155/2020; JREC/327/19), Sally Mugabe Hospital Ethics Committee (071119/64; 250418/48). Results will be disseminated via academic publications and public and policy engagement activities. In this study, the care for an estimated 15,000 babies across three sites will be impacted.

For peer review only

Strengths and limitations of this study

- Mixed methods intervention co-development and pilot implementation underpinned by behavioural science frameworks will optimise acceptability, feasibility and usability of the NeoTree, and the implementation strategy for larger scale roll out.
- Piloting of quantitative and qualitative clinical, quality of care, process, and economic measures will allow for a robust protocol for larger scale evaluation
- Collecting case fatality rate data from five hospitals in low resource settings will ensure informed sample size calculations for larger scale evaluation.
- Clinical, quality of care and demographic data from an anticipated 15,000 sick and vulnerable babies will enable studies of patterns, causes and risk factors for mortality and key diagnoses (e.g., sepsis/ neonatal encephalopathy).
- Clinical and cost effectiveness data will not be possible through this study design

Word count: 3946

For peer review only

For peer review only - http://bmjopen.bmj.com/site/about/guidelines.xhtml

INTRODUCTION

Worldwide, 2.4 million children younger than 28 days die yearly, accounting for 44% of deaths in children under five.(1) Approximately 70% of newborn deaths are avoidable through the implementation of simple, evidence-based interventions.(2) Health systems strengthening and training in newborn care are key to saving newborn lives.(3-5) Implementation of evidence-based guidelines can be supported through provision of reliable data systems, clinical decision support tools and education.(6, 7) We are developing and robustly evaluating an integrated quality improvement system for hospital-based sick and vulnerable newborns: the NeoTree. The NeoTree combines evidence-based clinical guidelines with real-time newborn data collection, data visualisation and export and newborn education on one platform.(8) This tablet-based digital system is for use at the hospital bedside by healthcare professionals (HCP) with a range of skills and competencies (primarily nurses) supporting the care and treatment of newborns. The NeoTree development has followed standard software development and Medical Research Council (MRC) complex intervention development frameworks.(9)

Background work to the NeoTree

A literature review of HCP-led newborn interventions in low resource settings (LRS) identified gaps in successful implementation of proven interventions.(10) To address these gaps, we explored the concept and acceptability of digital data capture and clinical decision support via workshops with HCP in Bangladesh (n~15; 2014, unpublished) and developed a prototype app (2015). An editor platform was designed to allow a clinician to configure the data capture forms and NeoTree-alpha was configured (2016). Next, a qualitative study was conducted with HCP in Zomba Central Hospital (ZCH), Malawi to understand barriers to delivering quality newborn care and to explore the potential for digital health interventions to mitigate these.(8) We selected ZCH as a NeoTree co-investigator had previously piloted a clinical algorithm to support babies with respiratory distress at this site.(11) NeoTree-beta minimal viable product 1 (MVP-1) was subsequently co-developed with Malawian HCP (*n*=46, 2016-17) in the same clinical setting.(8) NeoTree-beta MVP-1 included data capture on admission, resuscitation clinical diagnostic and management support and associated educational material. Clinical management advice pages were designed and linked to HCP chosen diagnoses. The NeoTree was found to be acceptable, feasible and highly usable and the potential for electronic clinical audit data demonstrated.(8, 12) HCP reported improved perceived ability to deliver quality care.(8) The NeoTree implementation did not continue at Zomba Central Hospital due to lack of ongoing funding. Identified strategies to optimise implementation included the introduction of a technical support role (suggested title: NeoTree Ambassador).

For peer review only - http://bmjopen.bmj.com/site/about/guidelines.xhtml

In November 2018, the NeoTree was introduced at Sally Mugabe Central Hospital Neonatal Unit, Harare, Zimbabwe, at the request of local clinical teams,(13) presenting an opportunity to co-develop and test the NeoTree in a doctor-led unit within a new LRS with unique health system challenges. NeoTree Ambassador roles were included in deployment, discharge data capture was developed, and linkage between clinical and laboratory blood culture data was undertaken to improve management of neonatal sepsis. A second site was identified in Malawi - Kamuzu Central Hospital - for further development and piloting. Since May 2019 we have been further developing NeoTree-beta MVP-2 data capture on admission and discharge at this site, co-developing a data dashboard prototype and assessing usability, acceptability, barriers to implementation, usage and feasibility of both of these functions, using behavioural science frameworks. The data dashboard prototype included a summary statistics page, an admission hypothermia page and monthly morbidity mortality statistics developed using PowerBi.

Following this work, our priority has been the ongoing development and pilot implementation evaluation of remaining functions of the NeoTree, to create NeoTree-gamma. NeoTree-gamma will include data capture (admission, discharge and laboratory); clinical decision support (resuscitation and non-resuscitation diagnosis and management); education; and data transfer to local teams, dashboards and national databases. As HCP complete the admission, they will receive prompts to respond appropriately to the data they have entered and manage patients according to evidence-based guidelines. The NeoTree works offline to synchronise with a network when available. It is a not-for-profit venture with open-source code and can be preconfigured to adapt to available resources (medication and technology) within the index facility.

Aims and Objectives

Our primary research question is:

- *Can an integrated digital quality improvement system be implemented and sustained in two hospitals in Zimbabwe and one hospital in Malawi to improve newborn care?*

Secondary research questions include:

- *What is the case-fatality rate of intervention hospital neonatal units before and after implementation of the NeoTree?*
- *What is the case-fatality rate of two matched control hospital neonatal units not implementing the NeoTree during this time period?*
- *Can a predictive algorithm be developed to improve identification of neonatal sepsis?*
- *What is the sensitivity/specificity of the NeoTree sepsis algorithm versus gold standard?*

For peer review only - http://bmjopen.bmj.com/site/about/guidelines.xhtml

Study aims and objectives are to:

1. Further develop, implement and evaluate the NeoTree at three intervention sites;
2. Collect outcome data for newborns admitted to two hospitals where the NeoTree is not being implemented;
3. Test the clinical validity of the NeoTree neonatal sepsis diagnostic algorithm against gold standard diagnosis;
4. Add data visualisation and linkage to the NeoTree functionality;
5. Develop and test proof of concept for communicating daily electronic health records (EHR) using NeoTree.

METHODS AND ANALYSIS

Study design

This is a mixed methods intervention co-development, pilot implementation and economic evaluation study (which will run from 7th Oct 2019 to 6th April 2022). We will continue to follow the MRC complex intervention development framework.(14) Table 1 shows anticipated timeline and study overview.

Table 1: Intervention roll-out and study timelines

Months 1-4	Study set up	Protocol development, recruitment and securing necessary permissions/approvals.
Months 1-21	Ongoing embedding of NeoTree into standard care and continuation of data collection in SMCH and KCH as part of studies (REF: P.02/19/2613)	
Months 5-24	Implementation of NeoTree into standard clinical care at CPH	
Months 1-9 (or until sample size reached)	Clinical validation sub-study	Clinical validity of the diagnostic algorithms
Months 5-9	Ongoing data dashboard development and data linkage	Co-development and optimisation the dashboard via design/usability workshops
Months 6-21	Pilot implementation science evaluation	Qualitative studies with HCP (nurses, nursing students and doctors), hospital administration staff (senior doctors/nurses, managers), and parents/carers
Months 6-21	Collection of quality of newborn care measures	
Months 10-15	Testing concept of electronic record system	Configure and test linkage of NeoTree data to the MoH electronic record system
Months 1-21	Economic evaluation/costings data	During all phases, cost and resource implications data will be collected and analysed.
Months 21-24	Data analysis and write up	

For peer review only - http://bmjopen.bmj.com/site/about/guidelines.xhtml

Setting

This is a two-country study in Malawi and Zimbabwe where, in 2019, the neonatal mortality rates were 19.8 and 25.9 per 1000 births respectively.(15) In Zimbabwe, the NeoTree will continue to be implemented at Sally Mugabe Central Hospital, the largest of three newborn tertiary care facilities in Zimbabwe, delivering 12,000 newborns annually, where the 100-cot nursery often runs at 120-130% capacity. Audit data show case fatality rates of 210 deaths per 1000 admissions.(13) The NeoTree will also be introduced to clinical processes at Chinhoyi Provincial Hospital, Zimbabwe - a provincial level hospital, delivering 4500 newborns annually with audit data showing case fatality rates of 180 per 1000 admitted babies. Six-months of newborn clinical and outcome data will be retrospectively collected from two hospitals in Zimbabwe providing usual care: Mbuya Nehanda Maternity Hospital (part of the Parirenyatwa hospital group) and Bindura Provincial Hospital. Selection of comparator hospitals is based on geographic proximity and similarities in catchment area and service level provision. Mbuya Nehanda Maternity Hospital and Sally Mugabe Central Hospital are two of six central hospitals in Zimbabwe, and both deliver primarily doctor-led care. Chinhoyi and Bindura Provincial Hospitals are two of eight provincial hospitals in Zimbabwe and deliver predominantly nurse-led newborn care. In Malawi, NeoTree will continue to be delivered at Kamuzu Central Hospital, one of four central hospitals in Malawi, delivering 4500 newborns annually. The neonatal unit admits around 2600 babies per year (from both within and outside of the hospital) with a case fatality rate of 210 per 1000.

Study participants:

Study participants include frontline and managerial staff involved in the delivery of newborn care (e.g., nurses, doctors, and nursing students) at the three implementation sites, and other key personnel and stakeholders such as hospital managers and parents/caregivers of newborns admitted to newborn care units. We estimate a sample size of ~170 HCP and 30 parents/caregivers across three sites in a phased pilot evaluation of each new co-developed functionality of the NeoTree system.

Routine clinical admission, discharge and microbiological data will be prospectively recorded on the NeoTree system for all newborns admitted to newborn care units at the three intervention sites (~12,000 babies). A limited data set of admission and discharge data will be retrospectively collected from all newborns admitted to the newborn care units at two control hospitals in Zimbabwe over a 6-month period (~1200 babies).

Consent procedures

For peer review only

For peer review only - http://bmjopen.bmj.com/site/about/guidelines.xhtml

Informed written consent will be sought from participants who take part in semi-structured interviews and focus group discussions. Participants with low literacy levels may give verbal consent which will be audio recorded and witnessed. We will follow international and local precedent for collection of neonatal pseudonymised data for the purposes of epidemiological surveillance and service evaluation such as the neonatal UK/ Australia/ New Zealand Badger net system,(16) and the WHO-led District Health Information Software (DHISv2).(17) Hence, we will not obtain informed consent from guardians to enter patient level data. No novel data will be recorded beyond that usually documented for clinical management, and no new procedures performed.

(i) **Co-development and optimisation of the NeoTree**

Data capture functions will be optimised and validated; data dashboards and clinical decision support functions will be fully developed; data linkage to Zimbabwean national EHR and aggregate data DHISv2 systems demonstrated; and daily EHR functionality will be piloted.

Data capture

A neonatal data dictionary will be developed in line with similar existing guidelines.(18, 19) It will define the types and formats of data captured in NeoTree, including standard data definitions to make it accessible to all data users. This will be made publicly available alongside open source code on GitHub. Data capture forms for admission, discharge and laboratory will be refined according to usability feedback. Data quality will be reviewed monthly to assure completeness and consistency of data by monitoring and reducing missing data where applicable. Periodic prospective paper audits comparing NeoTree capture of admissions and discharges to those recorded by ward paper records will be conducted. Data pipeline will be optimised to ensure automated data processing aligned with clinicians' needs and secure backup in UCL research database.

Dashboard development

The prototype initially developed at Kamuzu Central Hospital will be further developed in design/usability workshops with end-users. In both countries, one-to-one 'in vitro' user-tests will be conducted using a think aloud approach and an adapted user experience topic guide. During these sessions, pages of the dashboard (MVP-1) will be presented to HCP to gauge understanding and interpretation of the visualisations and collect feedback. Usability themes will be generated and analysed using agile rapid analysis. Following one-to-one user tests, participants will be invited to attend a videoed group workshop to consider new visualisations for MVP-2-dashboard. Optimal dashboard software will be identified.

Clinical decision support

For peer review only - http://bmjopen.bmj.com/site/about/guidelines.xhtml

Clinical decision support will be refined according to best available evidence and operationalised within the system. With the NeoTree, we will have a context-specific, detailed stream of clinical data at admission and outcome (discharge/death), in combination with laboratory diagnostic data for neonatal sepsis. With these data, we will build models to predict which babies are likely to benefit from specific interventions such as antibiotics. Clinical validation of the sepsis algorithm will be conducted retrospectively using data collected from Sally Mugabe and Kamuzu Central Hospitals. Admission diagnoses from the admitting healthcare professional, the senior clinician (based on admission data alone) and blood culture results (gold standard) will be retrospectively compared with the NeoTree algorithm diagnosis. Assuming sensitivity and specificity of 92% (lower 95% CI: 84%) >222 babies would need to be diagnosed with blood culture positive sepsis over five months, during which more than 2000 babies will be admitted with sepsis across sites.(20)

Data linkage

We will develop and demonstrate the ability for NeoTree data to be exported to the Zimbabwean national EHR system and to DHISv2, the most commonly used aggregate data system for reporting of health service data in the African region.

Daily electronic health record

A scoping study of the potential to extend NeoTree-gamma to include daily EHR will be conducted (*n*~20 babies).

(ii) Implementation evaluation

We will conduct qualitative studies of acceptability and feasibility, informed by behaviour science theories and frameworks. Qualitative and quantitative usability data will be gathered in addition to quantitative measures of usage, patterns in clinical outcomes and measures of quality newborn care.

Acceptability, feasibility and usability

Focus groups and individual interviews will be conducted with HCP, after each new functionality becomes embedded within clinical practice at three timepoints: pre-implementation (Chinhoyi Provincial Hospital only), implementation and sustainability.

In the pre-implementation phase (at baseline), we will explore current practice, quality improvement needs, and potential barriers and enablers to implementation at the new site of Chinhoyi Provincial Hospital, Zimbabwe. We will hold one focus group discussion with HCP (*n*~10) and approximately ten semi-structured interviews with senior doctors, nurses and hospital administrators.

For peer review only - http://bmjopen.bmj.com/site/about/guidelines.xhtml

In the implementation phase, we will deploy NeoTree to Chinhoyi Provincial Hospital while continuing to test and co-develop new functionalities across all sites. We will conduct three rounds of focus group discussions at each site (nine in total) with approximately ten participants in each (*n*~90). Perceived acceptability, feasibility and usability will be explored as follows:

- Round 1: Basic functionality of the NeoTree-beta MVP-2 (data capture at the bedside, clinical decision support for resuscitation and stabilisation and education) – Sally Mugabe Central and Chinhoyi Provincial Hospitals only.
- Round 2: Data dashboards (all sites)
- Round 3: Non-resuscitation clinical decision support (all sites)

To complement these data, we will conduct one set of individual interviews with approximately five senior clinical and managerial staff at each site (*n*~15). Topic guides for both focus groups and individual interviews will be semi-structured. Questions to explore acceptability of NeoTree will be based on the domains from the Theoretical Framework of Intervention Acceptability(21) e.g. burden, intervention coherence, opportunity costs, ethicality. Questions to explore barriers and enablers to implementing NeoTree in practice will be based on the Theoretical Domains Framework;(22) an integrative framework of 33 behaviour change theories proposing 14 domains of factors facilitating/hindering behaviour change e.g. knowledge, available resources, social influences, motivation, etc. Drafts of topic guides (Supplementary Files 1-3) will be reviewed and piloted before the final versions are implemented. Focus groups and interviews will last for a maximum of two hours and one hour respectively. These will be conducted by a trained researcher, either face-to-face in a private location in the hospital, or remotely via platforms such as Microsoft Teams, at a convenient date and time to participants.

Focus groups and interviews will be audio-recorded, transcribed verbatim, and fully anonymised. Anonymised data will be stored securely at UCL for 10 years. We will analyse the transcripts using a combined deductive framework and inductive thematic analytical approach,(23) to identify which domains are key influences on implementation and acceptability. To ensure reliability, a subset of 10% of transcripts will be double-coded by another researcher (KC). We will compare themes over time (i.e., across the implementation period), across countries, and according to professional role. Refreshments will be provided to those taking part and travel costs will be reimbursed where relevant. Following analyses of focus group and interview data, we will identify any potential refinements or additions to be made to NeoTree or the associated training materials, in order to improve acceptability, usability and address barriers and enablers to implementation. We will draw on behavioural science intervention development frameworks(24, 25) to identify relevant behaviour change techniques to address identified barriers and enablers.

For peer review only - http://bmjopen.bmj.com/site/about/guidelines.xhtml

In the sustainability phase, we will conduct a final round of data collection with ~10 healthcare professionals and ~5 senior clinical and managerial staff at each site (total *n*~45) with a focus on intervention sustainability once research and software development teams have withdrawn. No further changes to functionality will be made to NeoTree-gamma during this period. We will compare themes from sustainability versus initial implementation data collection period, across countries and across professional roles using the same methodology described above.

We will interview ~10 parents/carers of newborns at each of the three hospitals (~30 parents/carers) to explore their views on the use of digital innovations in healthcare, and the perceived acceptability of NeoTree. Interviews will be semi-structured, based on the Theoretical Framework of Acceptability.(21) Interviews will be conducted by an independent researcher, who is not involved in the provision of clinical care. Assurances will be given to parents/ caregivers that their participation will not affect the care of the baby or other family members. Findings will inform refinements of the NeoTree system and associated training to ensure it is acceptable both to those providing and receiving newborn care.

Analysis of routine health data

Admission data from intervention sites will be analysed monthly to estimate measures such as overall and disease specific case-fatality rate, quality of newborn care and usage. In Zimbabwe, newborn outcome data from comparator sites will be compared with those from intervention sites. In Malawi, aggregate routine case fatality data from representative health facilities will be compared with those from Kamuzu Central Hospital. These case fatality rate estimates will inform the sample size calculation for the large scale evaluation. We will test the feasibility of collecting the quality of newborn care endpoints shown in Table 2, to inform the data collection procedures in the large-scale evaluation. Most map directly to WHO standards of quality of maternal and newborn care in health facilities.(5)

Table 2: Quality of newborn care endpoints

**Temperature on admission:**<br>1. The proportion of all newborns who had a normal body temperature (36.5-37.5 deg C) at the first complete examination (60-120 min after birth) [WHO quality statement 1.1b]
**Documentation:**<br>2. The proportion of all newborns for whom there is documented information on a) body temperature, b) respiratory rate, c) HR, d) O2 saturations in air e) saturations in oxygen f) blood sugar, feeding behaviour d) absence or presence of danger signs, e) admission weight [WHO quality statement 1.1c]
**Resuscitation:**<br>3. The proportion of all newborns who were not breathing spontaneously after additional stimulation at the health facility who were resuscitated with a bag-and-mask. [WHO quality statement 1.5]<br>4. The proportion of all newborns who were not breathing spontaneously after additional stimulation at

For peer review only - http://bmjopen.bmj.com/site/about/guidelines.xhtml

the health facility who were resuscitated with a bag-and-mask within 1 min of birth. [WHO quality statement 1.5]
**Infection:**<br>5. The proportion of all newborns in the health facility ***with signs of infection*** who received injectable antibiotics. [WHO quality statement 1.7b]<br>6. The proportion of all newborns ***of mothers with signs of infection*** in the health facility who received injectable antibiotics. [WHO quality statement 1.7b]<br>7. Proportion of newborns ***with suspected severe bacterial infection*** who received appropriate antibiotic therapy. [WHO quality statement 1.8]<br>8. Proportion of newborns born to HIV +ve mothers who received Nevirapine on first day of life [discharge]<br>9. Proportion of neonates with low blood sugar who are treated with a feed or dextrose as appropriate.
**Data collection & health system:**<br>10. The proportion of all newborns currently in the health facility who have a patient identifier and individual clinical medical record*. [WHO quality statement 2.1]<br>11. The proportion of all newborns discharged from the health facility within the past 24 h who had an accurately completed record of processes of care, treatments, outcomes and **diagnoses** (with ICD code). [WHO quality statement 2.1]<br>12. Data are collected routinely in the health facility during labour, childbirth and the postnatal period (and used regularly to make decisions on quality improvement)<br>13. The proportion of newborns seen in the health facility in the past three months who fulfilled the facility's criteria for referral who were actually referred. [WHO quality standard 3.1]

(iii) Economic Evaluation-intervention costing

During all phases, cost and resource implication data will be collected and analysed. These include NeoTree development costs, training, planning and set up, implementation, and resource implications for the hospital/healthcare system. Costs of developing and implementing the NeoTree system will be collected through expenditure reports, time-use survey and interviews with the project staff (Supplementary Files 4-5). Information on potential impact on intervention hospitals will be collected through time-use surveys with all HCP involved in NeoTree development and implementation, supplemented with project records on their involvement in different intervention activities, such as software development and training workshops (i.e., opportunity costs). To measure the effect of implementation on time spent on procedures/activities in the delivery of newborn care pathways, a pilot time-use survey will be conducted (implementation phase) at all intervention sites and one comparable site in each setting, to record admission and discharge activities and time spent on each activity for around 10 newborn patients in each hospital (n=30 in total). The tools developed and used for economic data collection will be modified and adopted for use in a larger trial/evaluation study of NeoTree.

Protocol for large scale evaluation

Data from all phases will inform the study protocol, costings and implementation strategy for a future large-scale evaluation. Of note, the data collected here are insufficient and not designed to test clinical and cost effectiveness – rather they will be used to refine the NeoTree and to inform a

For peer review only

robust evaluation. Primary, secondary, process, quality of care and economic outcomes will be clarified alongside data collection procedures and our logic model.

Patient/Public Engagement

Our pilot acceptability and feasibility work in Malawi in 2016 indicated that HCP were concerned about parent perceptions of the use of the NeoTree in routine newborn care.(8) They reported that some parents were "*afraid they think you are taking the information someplace else*". Student-nurses reported that parents/guardians thought the tablets were a distraction and that "*we are just on social networks*", while others viewed it positively "*with gladness*".(8, 26) In response, we developed public information posters for the newborn care unit with strong visual elements. Feedback from HCP and parents informed the development of this protocol. We will develop a public engagement strategy and work with partners (including ArtGlo Africa and the UCL Co-Production Collective) to build the capacity of patient (particularly mothers/carers) to support dissemination of study results in catchment areas of intervention hospitals and to co-produce future iterations of the NeoTree.

Data management plan

Study protocols, training manuals and data collection tools will be made available on a study website. Upon study completion, all documents and record forms will be stored onsite for at least 10 years. Our team has had extensive discussions with clinical teams and Ministries of Health in setting up this study. Our agreed data management plan for newborn data is in Supplementary File 6. A subset of the anonymised research database will be made open source after publication of the main study findings to ensure maximum reach and accessibility.

Focus groups, semi-structured interviews and workshops will be recorded on two digital audio recording devices. Recordings will be anonymised and transcribed verbatim using password-protected laptops. Hard copies of field notes and transcripts will be anonymised and locked away. Soft anonymised copies will be stored on a secure laptop. Similarly, data collected through time-use surveys will be anonymized and stored on a secure laptop.

ETHICS AND DISSEMINATION

Research ethics approvals for this study were granted from: Malawi College of Medicine Research and Ethics Committee (P.01/20/2909; P.02/19/2613); University College London (17123/001; 6681/001; 17123/001; 5019/004); Medical Research Council Zimbabwe (MRCZ/A/2570); Biomedical Research Training Institute institutional review board, Zimbabwe (AP155/2020), Joint Research Ethics Committee, University of Zimbabwe review board (JREC/327/19), and Sally Mugabe Hospital Ethics Committee (HCHEC 250418/48; 071119/64).

For peer review only - http://bmjopen.bmj.com/site/about/guidelines.xhtml

Potential ethical considerations

There are no conflicts of interest. No new drugs/biologic agents will be administered to the participants during this study, nor will previously used agents be used in a new manner. No additional tests of clinical management will be initiated beyond those of standard practice. The NeoTree will support the consistent implementation of national/international evidence-based guidelines.

Dissemination

Our output management plan - co-developed with our partners and co-investigators - will be reviewed every three months. Discussions with the Zimbabwean Ministry of Health are ongoing about how the NeoTree could, if successful, be rolled out across the country. We will develop long-term data sharing and access procedures (including a Data Access Committee with independent academic and lay members to assess requests for aggregated data).

A stakeholder event (Zimbabwe) will focus on synergies in research needs between Malawi and Zimbabwe, using the NeoTree as a platform for African-driven research questions, projects and higher degrees. It will include presentations on Quality Improvement projects/audits completed using the NeoTree. An anonymised research database comprising clinical newborn data from all three hospital sites will be designed at the NeoTree collaborator meeting with a data management plan setting out conditions for access according to national/international guidelines. We will disseminate our discussions/conclusions in an opinion piece in a peer-reviewed journal.

Impact of COVID-19

Our research programme has continued throughout the COVID-19 pandemic.(27) Key changes to our research plan are described in Supplementary File 7.

Discussion

Few digital interventions in LRS have been well described and rigorously evaluated thus limiting their potential with respect to clinical and implementation effectiveness, sustainability and generalisability. The work described here will allow the completion of co-development of NeoTree-gamma with key functionalities configured, operationalised, tested and ready for larger scale roll out and evaluation across Zimbabwe and Malawi. Behavioural science theory and frameworks will be used to explore barriers/enablers to implementation and inform intervention refinement and development of strategies to encourage implementation on scale and sustainability longer term.(21-23) The economic evaluation will estimate pilot implementation costs and resource requirements for

For peer review only - http://bmjopen.bmj.com/site/about/guidelines.xhtml

sustainability and scale up, and affordability. Clinical outcome and cost data will inform sample size and resources for a large scale evaluated roll out.

Our overall vision is to use evidenced-based best practice and information technology to improve clinical decisions for newborn care and increase rates of newborn survival in under-resourced health care settings. We aim to create a locally led and curated database of aggregate newborn outcomes that can be used to robustly evaluate health systems, undertake healthcare planning and resource allocation at both the micro level (by site), regional, national and international levels. In addition, we are committed to open-source code ensuring the NeoTree code is freely available for countries and hospitals to adopt, modify and run, and enabling those hospitals and ministries to own and control their data.

This work addresses a primary sustainable development goal: reducing neonatal mortality in low resource settings and addresses key strategic aims of the Every Newborn Action Plan.(28) Our clinical algorithms and linkage to microbiology data aim to optimise the management of leading causes of newborn death. In this study, the care for an estimated 15,000 babies across the three test sites will be impacted by the NeoTree. Through successful rollout across Zimbabwe and Malawi - the care for nearly 300,000 babies could be improved annually.

For peer review only

For peer review only - http://bmjopen.bmj.com/site/about/guidelines.xhtml

Authors' contributions: MH and EW wrote the first draft of the protocol paper with all other co-authors inputting to content and approving the final draft. MH, EW and HG led the writing of the research ethics applications. MH designed the study methods with guidance and input from all co-authors. Co-authors also offered specific input and expertise as follows: FL, EW and KC on behavioural sciences methodology; FF, HG, AS, SC, GC, CC, DN, TC, THB, EK and MCB on clinical validation and implementation; MCB on quantitative analyses; ML on parent/carer qualitative study and public engagement; CC on usability testing, data dashboard development and assessment of impact on quality newborn care; YS, THB and NK on software development and data pipeline; and HHB on economic evaluation.

Funding statement: This work was supported by Wellcome Trust grant number 215742_Z_19_Z.

Competing interests' statement: The authors declare no competing interests

For peer review only

For peer review only - http://bmjopen.bmj.com/site/about/guidelines.xhtml

References

1. Liu L, Oza S, Hogan D, et al. Global, regional, and national causes of child mortality in 2000-13, with projections to inform post-2015 priorities: an updated systematic analysis. *Lancet (London, England)*. 2015;385(9966):430-40.
2. Knippenberg R, Lawn JE, Darmstadt GL, et al. Systematic scaling up of neonatal care in countries. *Lancet (London, England)*. 2005;365(9464):1087-98.
3. Lawn JE, Kinney MV, Black RE, et al. Newborn survival: a multi-country analysis of a decade of change. *Health Policy Plan*. 2012;27 Suppl 3:iii6-28.
4. Moxon SG, Lawn JE, Dickson KE, et al. Inpatient care of small and sick newborns: a multi-country analysis of health system bottlenecks and potential solutions. *BMC Pregnancy and Childbirth*. 2015;15(2):S7.
5. World Health Organisation. Standards for improving quality of maternal and newborn care in health facilities. Geneva WHO; 2016. Available from: https://apps.who.int/iris/bitstream/handle/10665/249155/9789241511216-eng.pdf;jsessionid=74464E5C8BD4821F8C1B93584988EEE0?sequence=1.
6. Agarwal S, Labrique A. Newborn health on the line: the potential mHealth applications. *Jama*. 2014;312(3):229-30.
7. Li Y-p, Fang L-q, Gao S-q, et al. Decision Support System for the Response to Infectious Disease Emergencies Based on WebGIS and Mobile Services in China. *PLOS ONE*. 2013;8(1):e54842.
8. Crehan C, Kesler E, Nambiar B, et al. The NeoTree application: developing an integrated mHealth solution to improve quality of newborn care and survival in a district hospital in Malawi. *BMJ Glob Health*. 2019;4(1):e000860.
9. Campbell M, Fitzpatrick R, Haines A, et al. Framework for design and evaluation of complex interventions to improve health. *Bmj*. 2000;321(7262):694-6.
10. Kesler E, Costello A, Heys M, et al. G259(P) A systematic review of health worker-led interventions to reduce mortality in low birth weight neonates in low and middle-income institutional settings. *Archives of Disease in Childhood*. 2015;100(Suppl 3):A112.
11. Crehan C, Colbourn T, Heys M, et al. Evaluation of 'TRY': an algorithm for neonatal continuous positive airways pressure in low-income settings. *Arch Dis Child*. 2018;103(8):732-8.
12. Crehan C, Kesler E, Chikomoni IA, et al. Admissions to a Low-Resource Neonatal Unit in Malawi Using a Mobile App: Digital Perinatal Outcome Audit. *JMIR Mhealth Uhealth*. 2020;8(10):e16485.
13. Gannon H, Chimhuya S, Chimhini G, et al. Electronic application to improve management of infections in low-income neonatal units: pilot implementation of the NeoTree beta app in a public sector hospital in Zimbabwe. *BMJ Open Quality*. 2021;10(1):e001043.
14. Craig P, Dieppe P, Macintyre S, et al. Developing and evaluating complex interventions: the new Medical Research Council guidance. *Bmj*. 2008;337:a1655.
15. The United Nations Inter-agency Group for Child Mortality Estimation (UN IGME). Available from: https://data.unicef.org/topic/child-survival/neonatal-mortality/
16. https://www.clevermed.com/badgernet/badgernet-neonatal [
17. https://www.dhis2.org/ [
18. https://public.vtoxford.org/ [
19. Imperial College London Neonatal dataset. Available from: https://www.imperial.ac.uk/media/imperial-college/medicine/dept-medicine/infectious-diseases/neonatology/Neonatal-dataset-ISB1595-release-1-version-22.pdf
20. Chu H, Cole SR. Sample size calculation using exact methods in diagnostic test studies. *J Clin Epidemiol*. 2007;60(11):1201-2; author reply 2.
21. Sekhon M, Cartwright M, Francis JJ. Acceptability of healthcare interventions: an overview of reviews and development of a theoretical framework. *BMC Health Serv Res*. 2017;17(1):88.

For peer review only

For peer review only - http://bmjopen.bmj.com/site/about/guidelines.xhtml

22. Francis JJ, O'Connor D, Curran J. Theories of behaviour change synthesised into a set of theoretical groupings: introducing a thematic series on the theoretical domains framework. *Implement Sci*. 2012;7:35.
23. Atkins L, Francis J, Islam R, et al. A guide to using the Theoretical Domains Framework of behaviour change to investigate implementation problems. *Implement Sci*. 2017;12(1):77.
24. Michie S, Richardson M, Johnston M, et al. The behavior change technique taxonomy (v1) of 93 hierarchically clustered techniques: building an international consensus for the reporting of behavior change interventions. *Ann Behav Med*. 2013;46(1):81-95.
25. Michie S, van Stralen MM, West R. The behaviour change wheel: a new method for characterising and designing behaviour change interventions. *Implement Sci*. 2011;6:42.
26. Crehan C, Huq T, Kesler E, et al. 073 A qualitative study of healthcare workers perceived barriers and facilitators to neonatal care in a central hospital in malawi. *Archives of Disease in Childhood*. 2018;103(Suppl 2):A30-A.
27. Chimhuya S, Neal SR, Chimhini G, et al. Indirect impacts of the COVID-19 pandemic at two tertiary neonatal units in Zimbabwe and Malawi: an interrupted time series analysis. *medRxiv*. 2021:2021.01.06.21249322.
28. Kinney MV, Cocoman O, Dickson KE, et al. Implementation of the Every Newborn Action Plan: Progress and lessons learned. *Semin Perinatol*. 2015;39(5):326-37.

For peer review only

Supplementary File 1

Draft Focus Group Discussion (FGD) and Semi-Structured Interview Topic Guides with Healthcare Professionals (nurses and doctors)

Topic Guide 1: Baseline SSI

Interview guide for NeoTree semi-structured interview

Aim: to explore practice pre-implementation

Timing: pre-implementation (months 5-6)

Participants: Healthcare Professionals

Version: 1.0

Introduction

Hello, thank you very much for taking the time to speak to me today. My name is ______ and I work for _____________. This interview will probably last around 1 hour. As a reminder, I am talking to you today, as I work for a study that aims to improve the care given to sick and vulnerable babies in hospitals.

Before we start, can I check whether you have:

- been told about the study
- had an information sheet
- signed a consent form
- agreed to audio recording of the discussion

At this point, do you have any questions about the purpose of the study, or the documents you've been given [e.g. PIS/ consent form]? Is there anything that isn't clear?

I want to reassure you that I work for a research organisation [name] and not [name of health facility]. Anything you tell me today is confidential and will not be shared with your colleagues. All personal and identifying information (such as your name/names of others) mentioned will be removed and replaced with a code.

I just want to remind you that your participation in this interview is entirely voluntary [i.e. it is your choice]. If you do not want to answer a question you can just say 'pass' and we will move on to the next question.

I am interested to understand your role at [health facility] in the provision of neonatal care and how care is delivered. There are no right or wrong answers to these questions; I am just interested in your views so please answer honestly.

If you want to take a break or stop at any point, please tell me. And if you wish to withdraw from the study you are completely free to do so at any point.

For peer review only

1) Warm up questions

   a) What is your role on the unit? How long have you worked here? Where did you complete your training?

2) Staffing

   a) How many nurses, midwives, student nurses are on the unit, what qualifications, what are their roles?
   b) Is there a paediatrician in the unit? If not can you elaborate on the doctors' support you receive in the unit?
   c) What professional training is offered to nurse/ doctors?

3) Services and Procedures

   a) What health care services are provided in the neonatal ward? How linked with maternity ward?
   b) What are existing roles/ responsibilities/ practices for neonatal care? (from admission to discharge, i.e. who does what? when? where?)

4) Pathways and communication

   a) Can you describe the pathways for referrals into and out of the neonatal unit? How well do referral pathways work in practice? What could be improved?
   b) How would you describe your relationship with your co-workers, doctors, management? Any challenges in team working/ communication?
   c) How would you describe morale among HCWs on the unit? What could be improved?

5) MIS

   a) How are patient data captured currently (admission/ discharge/ lab results)? Any challenges with data capture?
   b) How are data used for quality improvement?

6) Equipment and supplies

   a) What machinery is available in the unit?
   b) What lab facilities are there? Drug supplies and consumables?
   c) What equipment/ supplies do you feel are lacking? Any problems with procurement?

7) Challenges in provision of neonatal care

   a) What do you feel are the main challenges to providing neonatal care in this hospital?
   b) How do you feel neonatal services could be improved?

8) Closing remarks

   a) Is there anything you would like to ask me?

For peer review only

Thank you so much, we really appreciate the time you've taken to participate in this study.

For peer review only

Topic Guide 2: Baseline FGD

Topic guide for NeoTree focus groups based on Theoretical Framework of Acceptability (TFA) and the Theoretical Domains Framework (TDF)

Timing of focus group: Pre-implementation (months 5-6)

Participants: Healthcare Professionals

Version: 1.0

Introduction

Hello, thank you very much for taking the time to speak to me today. My name is ______ and I work for _____________. This discussion will probably between 1-2 hours. As a reminder, I am talking to you today, as I work for a study that aims to improve the care given to sick and vulnerable babies in hospitals.

Before we start, can I check whether you have:

- been told about the study
- had an information sheet
- signed a consent form
- agreed to audio recording of the discussion

At this point, do you have any questions about the purpose of the study, or the documents you've been given [e.g. PIS/ consent form]? Is there anything that isn't clear?

I just want to remind you that your participation in this discussion is entirely voluntary [i.e. it is your choice].

I am interested in your views about the implementation of the NeoTree at [health facility]. Your opinions as health care workers at the frontline of newborn care are very important to us. There are no right or wrong answers to these questions, just differing points of views. Please feel free to share your point of view even if it differs from what others have said. Keep in mind that we are just as interested in negative comments as positive comments.

Please give everyone a chance to speak, and please do not share what we discuss today outside this group. Please say your ID number before you speak and do not say names during the discussion as we want to keep them anonymised.

If you want to take a break or stop at any point, please tell me. And if you wish to withdraw from the study you are completely free to do so at any point.

Before we start, do you have any questions for me?

For peer review only

1) Warm up questions

What is your role at this hospital? How long have you worked here?

2) Introduction to NeoTree [demonstration of the NeoTree]

Today I'd like us to discuss the NeoTree, which is a digital health intervention that we are planning to introduce at [health facility] to improve the quality of newborn care. The NeoTree is an app. It allows you to enter in all the clinical information about the baby on admission and discharge. It gives you guidance on what is likely to be wrong with the baby and how to best treat and manage the baby, based on international guidelines. Whilst the baby is on the newborn care unit it will also provide an electronic linkage to any tests that are done to check for infection. The NeoTree also stores the clinical information for all of the babies and then feeds back to healthcare workers and the hospital on things like how many babies were admitted each month and what was wrong with them so that they can best plan the services needed.

Do you have any questions?

3) Knowledge & Skills (TDF)

a) How easy or difficult do you think it would be to use the NeoTree at [health facility]?

Prompt: What in particular would be easy? What in particular would be difficult?

b) Do you think you would need any additional skills or training to use NeoTree?

4) Beliefs about consequences (TDF)

a) What do you perceive to be the benefits/positives of using NeoTree?

Prompt: for: you as nurses/ doctors, other colleagues/roles and for patients & families?

b) Would there be any drawbacks?

Prompt: for you as nurses/ doctors, other colleagues/roles and for patients and families?

c) Overall, do you think the pros would outweigh the cons?

5) Burden (TFA) + Social Professional Role/Identity (TDF)

a) Do you think using the NeoTree would create extra work for you? In what way?

b) Do you feel it is would be your responsibility to use a tool like this? Who else might be responsible for completing the information in NeoTree or using it to guide decisions around care?

6) Social influences (TDF)

a) To what extent would the views of others influence whether you use the NeoTree?

For example the opinions of other nurses in the unit, other doctors, senior hospital managers, the NeoTree project management team, parents/guardians?

For peer review only

7) Opportunity costs (TFA)

Is there anything that you feel you would have to give up in order to use the NeoTree?

8) Ethicality (TFA)

a) Do you feel the NeoTree would be safe? For you and for babies?

b) Do you feel introducing the NeoTree would be fair?

9) Self-efficacy (TFA) & Beliefs about capabilities (TDF)

a) How confident would you be using the NeoTree? Is there anything in particular you would not be confident about?

b) Is there anything that could help you become more confident?

10) Environmental context and resources (TDF)

a) Do you think you would have sufficient resources to implement the NeoTree?

11) Goals (TDF)

a) Compared to other things you have to do, where does introducing the NeoTree fit in in terms of a priority? What would be the competing priorities?

b) Do you have any targets/goals for practice? How would the NeoTree fit in with that if at all?

12) Perceived effectiveness (TFA) + optimism (TDF)

In your view, how likely is the NeoTree to improve quality of newborn care?

13) Intentions (TDF)

a) Would you like to use the NeoTree? Can you explain your reasons why/why not?

b) To what extent do you intend to use it?

14) Barriers/ facilitators (general)

a) What do you think might be some of the barriers to introducing the NeoTree at [health facility]?

b) Is there anything that might make it easier to implement the NeoTree at [health facility]?

To summarise, the main points from our discussion are (facilitator to summarise key points). Is there anything else you'd like to add? Is there anything you'd like to ask me?

Thank you so much. We really appreciate the time you've taken to participate in this study.

For peer review only

Topic Guide 3: FGD - basic functionality of the NeoTree

Topic guide for NeoTree focus groups based on Theoretical Framework of Acceptability (TFA) and the Theoretical Domains framework (TDF)

Target behaviours – digital documentation on NeoTree, following emergency diagnostic guidelines, receiving educational support

Timing of focus group: implementation phase (months 6/7)

Participants: Healthcare Professionals

Version 1.0

Introduction

Hello, thank you very much for taking the time to speak to me today. My name is _______ and I work for ______________. This discussion will probably between 1-2 hours. As a reminder, I am talking to you today, as I work for a study that aims to improve the care given to sick and vulnerable babies in hospitals.

Before we start, can I check whether you have:

- been told about the study
- had an information sheet
- signed a consent form
- agreed to audio recording of the discussion

At this point, do you have any questions about the purpose of the study, or the documents you've been given [e.g. PIS/ consent form]? Is there anything that isn't clear?

I just want to remind you that your participation in this discussion is entirely voluntary [i.e. it is your choice].

I am interested in your views about the implementation of the NeoTree at [health facility]. Your opinions as health care workers at the frontline of newborn care are very important to us. There are no right or wrong answers to these questions, just differing points of views. Please feel free to share your point of view even if it differs from what others have said. Keep in mind that we're just as interested in negative comments as positive comments

Please give everyone a chance to speak, and please do not share what we discuss today outside this group. Please say your ID number before you speak and do not say names during the discussion as we want to keep them anonymised.

If you want to take a break or stop at any point, please tell me. And if you wish to withdraw from the study you are completely free to do so at any point.

Before we start, do you have any questions for me?

1) Warm up questions

What is your position at this hospital? How long have you worked here?

For peer review only

2) Intervention coherence (TFA)

We are going to ask you a few questions about NeoTree which as you know is a new digital platform we have started using on the ward.

a) Can you talk me through your understanding of what NeoTree is? What it is for?

Optional Prompt: What are the functions / objectives of the NeoTree?

3) Barriers (general)

a) What in general do you think are the barriers to using NeoTree in day to day practice?

b) Is there anything that made it easier or encouraged you to use NeoTree in day to day practice?

4) Knowledge & Skills (TDF)

a) Overall, 'how easy or difficult is it to use NeoTree?'

Prompt: What in particular is easy? What in particular is difficult?

b) Do you think you need any additional skills or training to use NeoTree?

5) Beliefs about consequences (TDF)

a) How does using NeoTree differ to the previous paper-based system for documentation?

b) Are there any benefits/positives of using NeoTree?

Prompt: for you as nurses, other colleagues/roles and for patients & families?

c) Are there any draw-backs/negatives?

Prompt: for you as nurses, other colleagues/roles and for patients and families?

d) Overall, do you think the pros outweigh the cons?

6) Burden (TFA)/ Beliefs about consequences

a) How does it affect your capacity to do your job? Does it help or hinder you to do your job?

b) How much effort does it require to use NeoTree compared to the paper form you were using before?

- does it require more time? more support from colleagues?

7) Affective attitude (TFA) + Emotion (TDF)

a) Do you like using NeoTree? What in particular do you like or dislike?

Prompt: Any concerns or worries about NeoTree?

For peer review only

8) Social influences (TDF)

a) To what extent do the views of others influence if and how you use the NeoTree? For example the opinions of other nurses in the unit, the NeoTree ambassador, doctors, the NeoTree project management team, parents/guardians?

b) Has the NeoTree changed team working and communication in the unit in any way?

Prompt: communication, roles, responsibilities?

9) Opportunity costs (TFA)

Is there anything that you feel you must give up in order to use the NeoTree?

10) Ethicality (TFA) Social/Professional role & identity (TDF)

a) Do you feel it is safe to use NeoTree? For you and for babies?

b) How does NeoTree fit in with your ways of working? Do you feel it is your responsibility to use a tool like this? Who else might be responsible for completing the information in NeoTree or using it to guide decisions around care?

11) Self-efficacy (TFA) Beliefs about capabilities (TDF)

a) How confident are you in using the NeoTree? Is there anything in particular you are not confident about?

b) Is there anything that could help you become more confident?

12) Environmental context and resources (TDF)

a) Do you have enough resources to use the NeoTree?

13) Memory, attention, decision making (TDF)

a) Do you use NeoTree to guide your decision-making? How so?

Does it make things easier/more difficult?

b) Have you ever forgotten to use the NeoTree?

If yes - in what kind of situations? and how can this be avoided in future?

c) Are there ever any instances when you decided to deviate from recommendations in NeoTree? Talk me through this....

d) Are there ever any instances where you decided not to use NeoTree? If so, can you talk me through this...

14) Goals (TDF)

a) How important is using NeoTree for you? And why?

b) Compared to other things you have to do, where does completing the NeoTree forms and using NeoTree fit in in terms of a priority? What are the competing priorities?

c) Do you have any targets/goals for practice? How does NeoTree fit in with that if at all etc

For peer review only

15) Perceived effectiveness (TFA) + TDF optimism

a) In your view, how likely is the NeoTree to improve quality of newborn care?

16) Reinforcement (TDF)

a) What positive experiences have you had with the NeoTree that would encourage you to use it again? Are there any negative experiences that would discourage you from using NeoTree again?

b) Are there any incentives / rewards / pressures to use NeoTree?

17) Intentions (TDF)

a) Do you intend to use the NeoTree in your day-to-day practice?

18) Behavioural regulation (TDF)

a) To what extent has using NeoTree become habitual or something you do without thinking, day to day? Why/how so?

b) Do you ever review the data you enter into NeoTree? Use it to monitor practice? Discuss it with colleagues?

c) Have you ever encountered any problems using NeoTree? How did you overcome these?

d) Is there anything you think we can do to improve the implementation of the NeoTree on the ward?

To summarise, the main points from our discussion are (facilitator to summarise key points). Is there anything else you'd like to add? Is there anything you'd like to ask me?

Thank you so much. We really appreciate the time you've taken to participate in this study.

For peer review only

Topic Guide 4: FGD -data dashboard

Topic guide for NeoTree focus groups based on Theoretical Framework of Acceptability (TFA) and the Theoretical Domains Framework (TDF)

Target behaviours – Interpreting data from data dashboard; changing clinical practise/behaviours to achieve agreed quality improvement goals.

Timing of focus group: implementation phase (months 10/11)

Participants: Healthcare Professionals

Version 1.0

Introduction

Hello, thank you very much for taking the time to speak to me today. My name is _______ and I work for ______________. This discussion will probably between 1-2 hours. As a reminder, I am talking to you today, as I work for a study that aims to improve the care given to sick and vulnerable babies in hospitals.

Before we start, can I check whether you have:

- been told about the study
- had an information sheet
- signed a consent form
- agreed to audio recording of the discussion

At this point, do you have any questions about the purpose of the study, or the documents you've been given [e.g. PIS/ consent form]? Is there anything that isn't clear?

I just want to remind you that your participation in this discussion is entirely voluntary [i.e. it is your choice].

I am interested in your views about the implementation of the NeoTree at [health facility]. Your opinions as health care workers at the frontline of newborn care are very important to us. There are no right or wrong answers to these questions, just differing points of views. Please feel free to share your point of view even if it differs from what others have said. Keep in mind that we're just as interested in negative comments as positive comments

Please give everyone a chance to speak, and please do not share what we discuss today outside this group. Please say your ID number before you speak and do not say names during the discussion as we want to keep them anonymised.

If you want to take a break or stop at any point, please tell me. And if you wish to withdraw from the study you are completely free to do so at any point.

Before we start, do you have any questions for me?

For peer review only

1) Intervention coherence (TFA) / Knowledge (TDF)

   a) Have you noticed any recent changes to the NeoTree? Any recent additions?

      [show data dashboard]

   b) Can you talk me through the dashboard and what you think it hopes to achieve?

2) Knowledge & Skills (TDF)

   a) Overall, how easy or difficult is it to understand the information presented in the dashboards? What in particular is easy? What in particular is difficult?

   b) What would make the dashboards easier to understand?

3) Beliefs about consequences (TDF)

   a) How might having these dashboards change practice, if at all?

      Prompt- how do they influence your decision making/actions?

   b) What are the benefits of having these dashboards? Any downsides?

4) Burden (TFA)

   a) To what extent do you use the dashboard in your day-to-day job? Talk me through this

   b) Has using it had an impact on your workload in any way? i.e. generated more tasks, needed time away from other responsibilities etc

5) Affective attitude (TFA) & Emotion (TDF)

   a) Do you like using the data dashboards ? What in particular do you like or dislike?

   b) How helpful is the dashboard? Any concerns or worries?

6) Social influences (TDF)

   a) To what extent do the views of others influence if and how you use the dashboard? For example the opinions of other nurses in the unit, the NeoTree ambassador, doctors, the NeoTree project management team, parents/guardians?

   b) Has the NeoTree changed team working in the unit in any way?

      Prompt: communication, roles, responsibilities?

7) Opportunity costs (TFA)

   a) Is there anything that you feel you must give up in order to use the data dashboard?

   b) Given the choice between the basic NeoTree and NeoTree with data dashboard which would you choose to use, and why?

For peer review only

8) Ethicality (TFA) & Social/Professional role & identity (TDF)

a) Do you feel displaying data and quality improvement targets in the ward is fair? for you as a HCW?

b) Do you feel it is your responsibility to use a tool like this? Who else might be responsible to use the data dashboards?

9) Self-efficacy (TFA) & Beliefs about capabilities (TDF)

a) How confident are you using the data dashboard? What in particular are you more/less confident about?

b) What could help improve your confidence?

10) Environmental context and resources (TDF)

a) Do you have enough time to use the data on the data dashboard in your day-to-day practice?

b) Do you have the necessary resources available to work towards achieving the dashboard recommendations and targets?

11) Memory, attention, decision making (TDF)

a) Is there anything on the dashboards that grab your attention? What in particular?

b) How easy/difficult is it to extract key information from the dashboard?

c) Are there ever any instances when you decided to deviate from suggested behaviour and quality improvement target? Talk me through this....

12) Goals (TDF)

a) How important is using the data dashboard for you? And why?

b) Compared to other things you have to do, where does the data dashboard fit in terms of priority? What are the competing priorities?

c) Do you have any targets/goals for practice? How does the data dashboard fit in with that if at all?

13) Perceived effectiveness (TFA) + Optimism (TDF)

a) How likely is the data dashboard to improve quality of newborn care?

14) Reinforcement (TDF)

a) What positive experiences have you had with the data dashboard that would encourage you to keep using it?

b) What incentives / rewards / pressures are there to use the data dashboard and achieve behaviour change targets?

For peer review only

15) Intentions (TDF)

a) To what extent do you intend to use the dashboards in your everyday work? Can you explain your reasons?

16) Behavioural regulation (TDF)

a) To what extent has using the data dashboard become habitual (i.e. something that you do routinely) in your day to day practice? Why/how so?
b) Have you ever encountered any problems using the dashboards? What were these and how did you overcome these?
c) What can we do to improve the implementation of the dashboards on the ward??

To summarise, the main points from our discussion are (facilitator to summarise key points). Is there anything else you'd like to add? Is there anything you'd like to ask me?

Thank you so much. We really appreciate the time you've taken to participate in this study.

For peer review only

Topic Guide 5: FGD - activated non-emergency clinical algorithm

Topic guide for NeoTree focus groups based on Theoretical Framework of Acceptability (TFA) and the Theoretical Domains Framework (TDF)

Target behaviours – use of the non-emergency algorithm to diagnose and manage newborns with sepsis and HIE.

Timing of focus group: implementation phase (month 16)

Participants: Healthcare Professionals

Version: 1.0

Introduction

Hello, thank you very much for taking the time to speak to me today. My name is _______ and I work for _______________. This discussion will probably between 1-2 hours. As a reminder, I am talking to you today, as I work for a study that aims to improve the care given to sick and vulnerable babies in hospitals.

Before we start, can I check whether you have:

- been told about the study
- had an information sheet
- signed a consent form
- agreed to audio recording of the discussion

At this point, do you have any questions about the purpose of the study, or the documents you've been given [e.g. PIS/ consent form]? Is there anything that isn't clear?

I just want to remind you that your participation in this discussion is entirely voluntary [i.e. it is your choice].

I am interested in your views about the implementation of the NeoTree at [health facility]. Your opinions as health care workers at the frontline of newborn care are very important to us. There are no right or wrong answers to these questions, just differing points of views. Please feel free to share your point of view even if it differs from what others have said. Keep in mind that we're just as interested in negative comments as positive comments

Please give everyone a chance to speak, and please do not share what we discuss today outside this group. Please say your ID number before you speak and do not say names during the discussion as we want to keep them anonymised.

If you want to take a break or stop at any point, please tell me. And if you wish to withdraw from the study you are completely free to do so at any point.

Before we start, do you have any questions for me?

For peer review only

1) Intervention coherence (TFA) / Knowledge (TDF)

a) Have you noticed any recent changes to the NeoTree? Any recent additions?

[show diagnostic tool]

b) Can you talk me through the diagnostic tool and what you think it hopes to achieve?

2) Knowledge & Skills (TDF)

a) Overall, how easy or difficult to use the non-emergency diagnostic tool? What in particular is easy? What in particular is difficult?

b) What would make the diagnostic tool easier to use?

3) Beliefs about consequences (TDF)

a) How might having the diagnostic tool change practice, if at all?

Prompt- how might it influence your decision making/actions?

b) What are the benefits of having the diagnostic tool? Any downsides?

4) Burden (TFA)

a) How does the new diagnostic tool affect your workload?

Prompt: Do you have any extra tasks with the addition of the diagnostic tool? Does it require more effort/ time compared to when the NeoTree did not have this function?

5) Affective attitude (TFA) and Emotion (TDF)

a) Do you like using the diagnostic tool? What in particular do you like or dislike?

Optional Prompt: Any concerns or worries?

6) Social influences (TDF)

a) To what extent do the views of others influence if and how you use the diagnostic tool? For example the opinions of other nurses in the unit, the NeoTree ambassador, doctors, the NeoTree project management team, parents/guardians?

b) Has the diagnostic tool changed team working in the unit in any way?

Prompt: communication, roles, responsibilities?

7) Opportunity costs (TFA)

a) Is there anything that you feel you must give up in order to use the new diagnostic tool?

8) Ethicality (TFA) & Social/Professional role & identity (TDF)

a) Do you feel the use of the NeoTree to diagnose and manage sepsis and HIE is safe? For you and for babies?

b) How does NeoTree fit in with your ways of working? Do you feel it is your responsibility to use a tool like this? Who else might be responsible for using the diagnostic tool?

For peer review only

9) Self-efficacy (TFA) Beliefs about capabilities (TDF)

a) How confident do you feel using the diagnostic tool?

b) Is there anything that could help you become more confident?

10) Environmental context and resources (TDF)

a) Do you have enough time to use the diagnostic tool?

b) Do you have the necessary resources available to use the diagnostic tool?

11) Memory, attention, decision making (TDF)

a) Do you use the diagnostic tool to guide your decision-making? Does it make things easier / more difficult? How so?

b) Have you ever forgotten to use the diagnostic tool?

If yes - in what kind of situations?

c) Are there ever any instances when you decided to deviate from the 1) the recommended diagnosis 2) the recommended management plan. Talk me through this….

d) Are there ever any instances where you decided not to use the diagnostic tool? If so, can you talk me through this…

12) Goals (TDF)

a) How important is using the diagnostic tool for you? And why?

b) Compared to other things you have to do, where does the diagnostic tool fit in terms of priority? What are the competing priorities?

c) Do you have any targets/goals for practice? How does the diagnostic tool fit in with that if at all?

13) Perceived effectiveness (TFA) and optimism (TDF)

a) How likely is the diagnostic tool to improve quality of newborn care?

14) Reinforcement (TDF)

a) What positive experiences have you had with the diagnostic tool that would encourage you to keep using it? Are there any negative experiences that would discourage you?

b) What incentives / rewards / pressures are there to use the diagnostic tool?

15) Intentions (TDF)

a) Do you intend to use the diagnostic tool in your day-to-day work? Can you explain your reasons?

For peer review only

16) Behavioural regulation (TDF)

a) To what extent has using the diagnostic tool become habitual (i.e. something that you do routinely) in your day to day practice? Why/how so?

b) Have you ever encountered any problems using the diagnostic tool? What were these and how did you overcome these?

c) What can we do to improve the use of the diagnostic tool on the ward?

To summarise, the main points from our discussion are (facilitator to summarise key points). Is there anything else you'd like to add? Is there anything you'd like to ask me?

Thank you so much. We really appreciate the time you've taken to participate in this study.

For peer review only

Topic Guide 6: FGD - sustainability

Topic guide for NeoTree focus groups based on Theoretical Framework of Acceptability (TFA) and the Theoretical Domains Framework (TDF)

Target behaviours – Use of all functions of the NeoTree as part of routine care (without support of NeoTree team)

Timing of focus group: sustainability phase (months 16-21)

Participants: Healthcare Professionals

Version: 1.0

Introduction

Hello, thank you very much for taking the time to speak to me today. My name is ______ and I work for _____________. This discussion will probably between 1-2 hours. As a reminder, I am talking to you today, as I work for a study that aims to improve the care given to sick and vulnerable babies in hospitals.

Before we start, can I check whether you have:

- been told about the study
- had an information sheet
- signed a consent form
- agreed to audio recording of the discussion

At this point, do you have any questions about the purpose of the study, or the documents you've been given [e.g. PIS/ consent form]? Is there anything that isn't clear?

I just want to remind you that your participation in this discussion is entirely voluntary [i.e. it is your choice].

I am interested in your views about the implementation of the NeoTree at [health facility]. Your opinions as health care workers at the frontline of newborn care are very important to us. There are no right or wrong answers to these questions, just differing points of views. Please feel free to share your point of view even if it differs from what others have said. Keep in mind that we're just as interested in negative comments as positive comments

Please give everyone a chance to speak, and please do not share what we discuss today outside this group. Please say your ID number before you speak and do not say names during the discussion as we want to keep them anonymised.

If you want to take a break or stop at any point, please tell me. And if you wish to withdraw from the study you are completely free to do so at any point.

Before we start, do you have any questions for me?

For peer review only

1) Introductory questions

NeoTree has been implemented on this unit/ward for X months now.

a) How much, if at all, do you think NeoTree is still used in day-to-day practice at the moment?

b) Has this changed over time (i.e. increased/ decreased)?

2) Knowledge & Skills (TDF)

a) How easy or difficult is it to use the NeoTree? Is it more or less easy to use the NeoTree since the NeoTree team have left?

b) Do you think any additional training, information or support is needed to continue to use NeoTree now that the research team are no longer present/ have left?

If so, what would be needed?

3) Beliefs about consequences (TDF)

a) Do you think there are any benefits to continuing to use the NeoTree in future?

b) Do you think there are any drawbacks to continuing to use the NeoTree in future?

c) Do the pros outweigh the cons?

4) Burden (TFA)

a) Has your role changed at all since the team has left? How?

b) How does the NeoTree affect your capacity to do your job? Does it help or hinder you to do your job?

5) Affective attitude (TFA) and Emotion (TDF)

a) Do you like using NeoTree? What in particular do you like or dislike now that the team has left?

b) Have your feelings about using the NeoTree changed since the team has left?

6) Social influences (TDF)

a) Do you have any concerns or fears about using the NeoTree now that the team has left?

b) Do the views of others influence if and how you use the NeoTree? For example the opinions of other nurses in the unit, the NeoTree ambassador, doctors, parents/guardians

c) Since the team left, has the NeoTree changed team working in the unit? In what way? (e.g. communication, roles. Responsibilities?)

Optional prompt: Any disagreements/ conflict?

For peer review only

7) Opportunity costs (TFA)

a) Is there anything that you feel you must give up in order to use the NeoTree? Do you feel that you have given up anything new since the team has left?

b) Given the choice, would you prefer the team to return? Can you explain your reasons?

8) Ethicality (TFA) Social/Professional role & identity (TDF)

a) Do you feel it is safe to continue to use NeoTree? For you and for babies? Do you feel that the NeoTree is more/ less safe since the team has left?

b) Do you feel using the NeoTree is fair now that the team has left?

c) Now that the team has left, how does NeoTree fit in with your ways of working?

d) Going forward, do you feel it is your responsibility to continue to use the NeoTree? Who else might be responsible to ensure the sustained use of the NeoTree?

9) Self-efficacy (TFA) Beliefs about capabilities (TDF)

a) Since the team has left, are you more or less confident to use the NeoTree?

b) Which aspects of the NeoTree are you confident about?

c) Which aspects are you less confident about? What could help to improve your confidence going forward?

10) Environmental context and resources (TDF)

a) Now that the team has left, do you have enough time to use the NeoTree?

b) Do you have the necessary resources to continue to use the NeoTree in future?

What else would be needed?

11) Memory, attention, decision making (TDF)

a) Have you ever forgotten to use the NeoTree since the team has left? Talk me through this

b) Since the team left, have there been instances when you decided not to use the NeoTree? Talk me through this, what did you do instead?

c) Since the team left, have there been instances when you decided not to follow the guidance of the NeoTree? Talk me through this, what did you do instead?

12) Goals (TDF)

a) How important is using NeoTree for you? And why? Has this changed since the team left?

b) Compared to other things you have to do, where using NeoTree fit in in terms of a priority? What are the competing priorities?

c) Do you have any targets/goals for future practice? How does NeoTree fit in with that if at all?

For peer review only

13) Perceived effectiveness (TFA) + Optimism (TDF)

a) Going forward, how likely is the NeoTree to improve quality of newborn care?

14) Reinforcement (TDF)

a) What positive experiences have you had with the NeoTree that would encourage you to use it in the long term? Are there any negative experiences that would discourage you from using NeoTree in the long term?

b) Are there any incentives / rewards / pressures to continue to use NeoTree in future?

15) Intentions (TDF)

a) To what extent do you intend to use NeoTree in the future? Why/ why not?

16) Behavioural regulation (TDF)

a) Have you encountered any problems using NeoTree since the team has left? How did you overcome these?

b) What needs to be done to support use of NeoTree longer term? (what, by whom, where, when?)

To summarise, the main points from our discussion are (facilitator to summarise key points). Is there anything else you'd like to add? Is there anything you'd like to ask me?

Thank you so much. We really appreciate the time you've taken to participate in this study.

For peer review only

For peer review only

Supplementary File 2

Draft topic guides for semi-structured interviews (SSIs) with senior managers and clinical staff

SSI guide 1 – Baseline

Interview guide based on Theoretical Framework of Acceptability/ Theoretical Domains Framework (TDF).

Target behaviour: intention to introduce and implement the NeoTree

Timing of interviews: pre- implementation phase (months 5-6)

Participants: senior managers/ administrative staff/ clinicians

Version 1.0

Introduction

Hello, thank you very much for taking the time to speak to me today. My name is ______ and I work for _____________. This interview will probably last around 1 hour. As a reminder, I am talking to you today, as I work for a study that aims to improve the care given to sick and vulnerable babies in hospitals.

Before we start, can I check whether you have:

- been told about the study
- had an information sheet
- signed a consent form
- agreed to audio recording of the discussion

At this point, do you have any questions about the purpose of the study, or the documents you've been given [e.g. PIS/ consent form]? Is there anything that isn't clear?

I want to reassure you that I work for a research organisation [name] and not [name of health facility]. Anything you tell me today is confidential and will not be shared with your colleagues. All personal and identifying information (such as your name/names of others) mentioned will be removed and replaced with a code.

I just want to remind you that your participation in this interview is entirely voluntary [i.e. it is your choice]. If you do not want to answer a question you can just say 'pass' and we will move on to the next question.

I am interested in your views about the introduction of the NeoTree at [health facility]. There are no right or wrong answers to these questions; I am just interested in your views so please answer honestly.

If you want to take a break or stop at any point, please tell me. And if you wish to withdraw from the study you are completely free to do so at any point.

Before we start, do you have any questions for me?

For peer review only

1) Warm up questions

What is your position at this hospital? How long have you worked here?

2) Introduction/ demonstration of the NeoTree

The NeoTree is a tablet-based clinical app, NeoTree, which combines evidence-based clinical guidelines with real-time data collection and education about newborn clinical needs. The NeoTree aims to improve quality of care and newborn survival through:

A) data-capture,

B) emergency decision-support

C) non-emergency clinical and management decision-support

D) feedback of data to dashboards and national aggregate data systems.

Health care workers (HCWs) use the app at the bedside to admit and discharge each newborn patient. As they complete the admission, they receive prompts to respond appropriately to the data they have entered and manage patients according to evidence-based guidelines. We also plan to link to national data systems (Electronic Medical Records).

Do you have any questions?

1) Knowledge & Skills (TDF)

a) How easy or difficult do you think it would be to introduce the NeoTree at [HEALTH FACILITY]?

Prompt: What in particular would be easy? What in particular would be difficult?

b) Do you think the staff would require any additional skills or training to use/ manage NeoTree?

2) Beliefs about consequences (TDF)

a) What do you perceive to be the benefits/positives of introducing the NeoTree at [HEALTH FACILITY]?

Prompt: for: you, other colleagues/roles and for patients & families?

b) Would there be any drawbacks?

Prompt: for you, other colleagues/roles and for patients and families?

c) Overall, do you think the pros would outweigh the cons?

3) Burden (TFA + Social Professional Role/Identity (TDF)

a) Do you think using the NeoTree would create extra work for you, for other staff? In what way?

b) Do you feel it is your responsibility oversee the introduction of the NeoTree? Who else might be responsible?

For peer review only

4) Affective attitude (TFA) and Emotion (TDF)

a) In general, how would you feel about the introduction and use of the NeoTree at [health facility]?

Positive emotions: pride, confidence, satisfaction, reassurance
Negative emotions; guilt, worry, concern, pressure

b) Do you have any worries or concerns about introducing digital aids such as the NeoTree into clinical care at this hospital?

5) Social influences (TDF)

a) Do you know of other hospitals using NeoTree or digital innovations? To what extent has this impacted on your decision to introduce the NeoTree in this facility?

6) Perceived Opportunity costs (TFA)

a) Do you think introducing the NeoTree would be a good use of resources?

7) Perceived Self-efficacy (TFA) Beliefs about capabilities (TDF)

a) To what extent do you feel confident staff in this facility could implement Neotree? What else would be needed?

8) Ethicality (TFA)

a) Do you feel the NeoTree would be safe? For you and for babies?

b) Do you feel introducing the NeoTree would be fair for you? For other staff?

9) Environmental context and resources (TDF)

a) Do you think you would have sufficient resources to implement the NeoTree?

10) Goals (TDF)

a) Compared to other things you have to do, where does introducing the NeoTree fit in in terms of a priority? What would be the competing priorities?

b) Do you have any targets/goals for practice? How would the NeoTree fit in with that if at all?

11) Perceived effectiveness (TFA) + optimism (TDF)

In your view, how likely is the NeoTree to improve quality of newborn care at [HEALTH FACILITY]?

12) Intentions (TDF)

a) Would you like this hospital to use the NeoTree? Can you explain your reasons why/why not?

13) Barriers/ facilitators (general)

a) What do you think might be some of the barriers to introducing the NeoTree at [HEALTH FACILITY]?

For peer review only

b) Is there anything that might make it easier to implement the NeoTree at [HEALTH FACILITY]?

14) Closing questions/ remarks

a) Now that we've completed the interview, is there anything you'd like to ask me or do you have anything further to add?

Thank you so much, we really appreciate the time you've taken to participate in this study.

For peer review only

SSI guide 2 - Implementation

Interview guide based on Theoretical Framework of Acceptability/ Theoretical Domains Framework (TDF).

Interview guide to explore (A) perceptions and understanding of quality newborn care and (B) acceptability of Neotree/ barriers and facilitators to implementation

Timing of interviews: implementation phase (months 6-16)

Participants: senior managers/ administrative staff/ clinicians

Version 1.0

Introduction

Hello, thank you very much for taking the time to speak to me today. My name is _______ and I work for ______________. This interview will probably last around 1 hour. As a reminder, I am talking to you today, as I work for a study that aims to improve the care given to sick and vulnerable babies in hospitals.

Before we start, can I check whether you have:

- been told about the study
- had an information sheet
- signed a consent form
- agreed to audio recording of the discussion

At this point, do you have any questions about the purpose of the study, or the documents you've been given [e.g. PIS/ consent form]? Is there anything that isn't clear?

I want to reassure you that I work for a research organisation [name] and not [name of health facility]. Anything you tell me today is confidential and will not be shared with your colleagues. All personal and identifying information (such as your name/names of others) mentioned will be removed and replaced with a code.

I just want to remind you that your participation in this interview is entirely voluntary [i.e. it is your choice]. If you do not want to answer a question you can just say 'pass' and we will move on to the next question.

I am interested in your views about the implementation of the NeoTree at [health facility]. There are no right or wrong answers to these questions; I am just interested in your views so please answer honestly.

If you want to take a break or stop at any point, please tell me. And if you wish to withdraw from the study you are completely free to do so at any point.

Before we start, do you have any questions for me?

1) Warm up questions

What is your position at this hospital? How long have you worked here?

For peer review only

2) Quality of newborn care

a) What does high quality newborn care mean to you?

Prompt- What do you understand by high quality care?

b) Who do you see as the providers of care in the neonatal unit?

Prompt - nurses, students, doctors, mother, other family members

Now I'd like to ask you some questions about the NeoTree. I'm really interested to understand your views and feelings with regard to the NeoTree.

3) Intervention coherence (TFA)

a) Can you talk me through what your understanding of NeoTree is? What it involves, its purpose, who it is intended to be used by, when, where etc?

4) Barriers/ enablers (general)

a) What have been the barriers to implementing NeoTree in this hospital?

b) What would help make it easier to implement the NeoTree here?

5) Experienced affective attitude (TFA) and Emotion (TDF)

a) In general, how do you feel about the introduction and use of the NeoTree at [health facility]?

Positive emotions: pride, confidence, satisfaction, reassurance
Negative emotions; guilt, worry, concern, pressure

b) Specifically, how useful do you find the different functionalities of the NeoTree:

- Data capture (i.e. admission, discharge and lab data);
- Diagnostic support (emergency and non-emergency);
- Data dashboards and quality improvement goals
- Data linkage to local and national databases

c) Do you have any worries or concerns about introducing digital aids such as the NeoTree into clinical care at this hospital?

6) Experienced burden (TFA)

a) Has the NeoTree required any extra time/ work/ resources? For you personally, from your staff?

7) Ethicality (TFA)

a) Do you think that the NeoTree is safe? For staff, for patients? Any concerns/ worries?

b) Has the NeoTree led to any changes in team working and communication? Have these been positive or negative? Any divisions between staff or conflicts?

For peer review only

8) Experienced opportunity costs (TFA)

a) Do you think the NeoTree is a good use of resources? Why/why not?

9) Experienced effectiveness (TFA)

a) At this stage of implementation to what extent do you feel the NeoTree has improved quality of newborn care? What in particular has helped improve quality of care?

10) Experienced self-efficacy (TFA)

a) What is your role in the implementation of the NeoTree?

b) How confident do you feel in carrying out this role? Which aspect of your role do you like/ and which do you like less?

c) To what extent do you feel confident staff in this facility can implement Neotree? What else would be needed?

11) Social influences (TDF)

a) Do you know of other hospitals using NeoTree or digital innovations? Did that impact on your decision to implement NeoTree in this facility at all?

12) Intentions (TDF)

a) To what extent do you plan to use the NeoTree in the future? Can you explain your reasons?

13) Behavioural regulation (TDF)

a) Have you ever encountered any problems in implementing/introducing NeoTree to this facility? If so, can you talk me through these? How did you overcome these?

b) Is there anything you think we can do to improve the implementation of the NeoTree in this hospital?

14) Closing questions/ remarks

a) In your view, what 5 things are needed to improve quality of neonatal care in this hospital?

b) Now that we've completed the interview, is there anything you'd like to ask me or do you have anything further to add?

Thank you so much, we really appreciate the time you've taken to participate in this study.

For peer review only

SSI Guide 3 - Sustainability

Interview guide to explore acceptability of Neotree/ barriers and facilitators to sustainability. Based on Theoretical Framework of Acceptability (TFA)/ Theoretical Domains Framework (TDF)

Timing: Sustainability phase (months 16-21)

Participants: senior managers/ administrative staff/ clinicians

Version: 1.0

Introduction

Hello, thank you very much for taking the time to speak to me today. My name is ______ and I work for ____________. This interview will probably last around 1 hour. As a reminder, I am talking to you today, as I work for a study that aims to improve the care given to sick and vulnerable babies in hospitals.

Before we start, can I check whether you have:

- been told about the study
- had an information sheet
- signed a consent form
- agreed to audio recording of the discussion

At this point, do you have any questions about the purpose of the study, or the documents you've been given [e.g. PIS/ consent form]? Is there anything that isn't clear?

I want to reassure you that I work for a research organisation [name] and not [name of health facility]. Anything you tell me today is confidential and will not be shared with your colleagues. All personal and identifying information (such as your name/names of others) mentioned will be removed and replaced with a code.

I just want to remind you that your participation in this interview is entirely voluntary [i.e. it is your choice]. If you do not want to answer a question you can just say 'pass' and we will move on to the next question.

I am interested in your views about the implementation of the NeoTree at [health facility]. There are no right or wrong answers to these questions; I am just interested in your views so please answer honestly.

If you want to take a break or stop at any point, please tell me. And if you wish to withdraw from the study you are completely free to do so at any point.

Before we start, do you have any questions for me?

For peer review only

1) Warm up questions

How are you? How have you been since we last spoke? Any change in terms of your role and responsibilities at the hospital?

The last time we spoke, I asked you a series of questions about your views and experiences of using the NeoTree. Since then, the NeoTree project managers and developers have left and no longer provide support on site.

I'd like to ask you some questions about how things have been since they left.

2) Intervention coherence (TFA)

a) To what extent do you think NeoTree is currently used/ has this changed over time/ why / how so
b) Has there been anything that has made it harder to implement NeoTree and sustain its use?
c) Anything that has made it easier?

3) Experienced affective attitude (TFA) & Emotions (TDF)

a) Have your feelings about the NeoTree changed since we last spoke [prompt previous answer].
b) Do you have any concerns or fears about implementing the NeoTree in this hospital now that the team has left

4) Experienced Burden (TFA)

Since we last spoke [prompt previous answers] do you think the NeoTree:

a) Has generated any extra work in terms of supervision or staffing?
b) Has required any extra time. Extra resources? from you personally, from your staff?
c) Since we last spoke, has the NeoTree diverted staff/ equipment/ funds away from other aspects of patient care? If so, what impact has this had on you personally, on other staff and on patients?

5) Ethicality (TFA)

a) Now that the team has left, do you think that the NeoTree is safe? For staff, for patients? Any changes/ concerns since we last spoke?
b) Since the last interview, do you think the NeoTree has led to any changes in team working and communication? Have these been positive or negative?

6) Experienced opportunity costs (TFA)

a) Do you think the NeoTree is a good use of resources? Why/why not? Has your opinion changed since we last spoke?

For peer review only

7) Experienced effectiveness (TFA)

a) Since we last spoke, do you feel the NeoTree has led to any further improvements in quality of care? What in particular has helped/ hindered quality of care?

8) Experienced self-efficacy (TFA)

a) Last time you told me that your role in the NeoTree is [insert from previous interview]. Has this changed since our last interview?

b) How confident do you feel in carrying out this role? Do you feel more or less confident since our last interview? Why?

c) To what extent do you feel confident staff in this facility can sustain NeoTree in the future? What else would be needed?

9) Intentions (TDF)

a) To what extent do you intend to sustain the NeoTree at this facility? Can you explain your reasons to me?

10) Barriers and enablers to sustainability (general)

a) What would it take to continue to use NeoTree going forward? What might get in the way of sustainability?

b) Is sustaining NeoTree a priority given other demands at this hospital?

c) What could help support sustainability longer term?

11) Closing questions/ remarks

a) Now that we've completed the interview, is there anything you'd like to ask me or do you have anything further to add?

Thank you so much, we really appreciate the time you've taken to participate in this study.

For peer review only

Supplementary File 3:

Parent/ Carer Interview Guide to explore (A) perceptions and understanding of quality newborn care and (B) acceptability of Neotree

Timing of interviews: implementation phase (months 6-16)

Version 1.0

Introduction

Hello, thank you very much for taking the time to speak to me today. My name is ______ [name of researcher] and I work for _____________. This interview will probably last around 1 hour. As a reminder, I am talking to you today, as I work for a study that aims to improve the care given to sick and vulnerable babies in hospitals.

Before we start, can I check whether you have:

- been told about the study
- had an information sheet
- signed a consent form
- agreed to audio recording of the discussion

At this point, do you have any questions about the purpose of the study, or the documents you've been given [e.g. PIS/ consent form]? Is there anything that isn't clear?

I want to reassure you that I work for a research organisation [name] and not [name of health facility]. Anything you tell me today will not affect the care of your baby or your family. All personal and identifying information (such as your name/names of others) mentioned will be removed and replaced with a code.

I just want to remind you that your participation in this interview is entirely voluntary [i.e. it is your choice]. If you do not want to answer a question you can just say 'pass' and we will move on to the next question.

I am interested in your views about the care of sick newborns, and your experiences of receiving care in this hospital. There are no right or wrong answers to these questions; I am just interested in your views so please answer honestly.

If you want to take a break or stop at any point, please tell me. And if you wish to withdraw from the study you are completely free to do so at any point.

As a reminder, your answers to these questions will not affect the care you or your family will receive at all. Do you have any questions for me?

For peer review only

1) Warm up questions

How are you feeling today? How long have you/ your baby/ family member been in the hospital? Where have you travelled from?

2) Quality of newborn care

a) What does high quality newborn care mean to you?

Prompt: What do you understand by high quality newborn care?

b) In your view, who provides most of the care in the neonatal unit?

Prompt: nurses, students, doctors, mothers, family members etc..

Acceptability of current care and use of digital aids

Now I'd like to ask you some questions about the NeoTree digital health aid. You probably noticed that when the HCW asked you questions about you/ your baby/ family member she/ he was using a tablet with the NeoTree app.

The NeoTree app uses the information that you give to the healthcare worker to work out what is likely to be wrong with the baby and how to best treat and manage the baby. Whilst the baby is on the newborn care unit it will also provide an electronic linkage to any tests that are done to check for infection. As well as providing information on what might be wrong with the baby, the NeoTree app also gives the healthcare workers education and tips around how to care for the newborn. The NeoTree also stores the clinical information for all of the babies and then feeds back to the healthcare workers and the hospital on things like how many babies were admitted each month and what was wrong with them so that they can best plan the services needed.

3) Experienced affective attitude (TFA) and Emotions (TDF)

Gathering and storing health information

a) How did you feel about answering questions about you and about your baby's health?

➢ Prompts to illicit feelings/ emotions:
Positive emotions: pride, confidence, satisfaction, reassurance
Negative emotions; guilt, worry, concern, pressure

b) How did you feel about the HCW entering your answers into the tablet [show visual aid]? Did you have any concerns about this at all?

c) Once your answers were entered into the tablet, your answers were stored electronically. How did you feel about the HCW storing (keeping) your answers on the tablet [show visual aid]?

Diagnosis and management of newborns

- How do you feel about the use of the NeoTree to support HCWs to diagnose and treat sick babies? [show example] Any concerns or worries?

For peer review only

4) Experienced burden (TFA)

a) How easy or difficult was it to answer the HCW's questions, when your baby was admitted using the NeoTree?

b) Were there any questions that were difficult or complicated to answer?

c) Do you think the NeoTree helped the HCWs provide care more efficiently to your baby? Did it get in the way at all? How so?

5) Experienced effectiveness (TFA)

a) Do you think the NeoTree was helpful for HCWs to provide care to babies? How so?

b) Do you feel the NeoTree has helped in the care of your baby? In what way?

c) Have you experienced any challenges while your baby has been in the unit?

6) Ethicality (TFA)

a) Did you feel fairly treated while they were using the NeoTree? Would you change any aspect of the way you were treated while they were using the NeoTree?

b) Do you think the NeoTree distracted HCWs from caring for your baby?

c) Do you have any safety concerns about the use of the NeoTree in the care of your baby?

7) Intervention coherence (TFA)

a) How clear was the information you were given about your baby's care [treatment/procedures]? Was anything not clear/ difficult to understand?

b) How do you think the data/information about you and your baby's health is used? What do you think happens to this information?

c) Do you think the NeoTree can help improve quality of newborn care? In what way?

8) Experienced opportunity costs (TFA)

a) Do you think there are any risks to you or your baby when the HCW uses NeoTree?

b) If you had the choice, would you prefer HCWs to record information about you and your baby on paper (i.e. not use the NeoTree)? If so why?

c) If you had the choice, would you prefer HCWs diagnose and manage your baby without the help of the NeoTree? If so, why?

9) Experienced self-efficacy (TFA)

a) How confident did you feel speaking to healthcare workers about the care of your baby?

b) How involved did you feel involved in the care of you baby? Clinical decisions etc?

For peer review only

10) Closing questions/ remarks

a) If you could change one thing about the care and treatment you've received at the unit, what would you change?

- Now that we've completed the interview, is there anything you'd like to ask me?

Thank you so much, we really appreciate the time you've taken to participate in this study.

For peer review only

For peer review only

Tool for collecting Non-Staff cost items. Adapted from "Haghparast-Bid

**Item/activity description** (Description of each item or activity should be specific and detailed)*	**Year**

Notes

* Among the non-staff costs, travel and overhead/joint costs will be the

** For items (such overhead, rent and running costs of an office, equipr overhead or running cost of an office, you can use for example, % of pe

For peer review only

For peer review only

ɟgoli, Skordis J et al: Protocol for the cost-consequence and equity impact analyses o

Line Item	% allocated to NeoTree (mainly relevant to capital/equipment, rent, and office overhead)**	Date/Time period (Optional)	Cost allocated to NeoTree (Local currency) or spcify currency if different

ɘ common cost items.
ment etc.) that are shared across different projects, you can use an allocation rule fo
ɘople working on the NeoTree activities, or % time spent on or space used for the Ne

For peer review only

Yes

No

For peer review only

›f a cluster randomised controlled trial comparing three variants of a nutrition-sensitive agricultura

**Is it possible to allocate the cost directly to the intervention components?**<br>If No, the cost item will be 100% joint cost. If yes, please specify % allocated to each component.				
	Data Entry (Admission and Discharges/Deaths)	Data back-up	M&M dashboard preparation	Research Meetings

›r allocating them to different projects. For example, for allocating
›oTree activities.

For peer review only

For peer review only

al extension intervention to improve maternal and child dietary diversity and nutritional status in rura

NeoTree activities (%)

Any other meetings relating to NeoTree	NeoTree Support/Testing/Pilot/Roll-out	NeoTree data pipeline	Diagnosis Data Entry	Data Quality Checks/Data audits/Data cleaning	Research (paper writing, etc)	Software development

For peer review only

For peer review only

l Odisha, India (UPAVAN trial) Trials (2019) 20:287"

MOH Database integration	Training	Clinical validation	Joint/admin	Check (should be 100%)
				0%
				0%
				0%
				0%
				0%
				0%
				0%
				0%
				0%
				0%
				0%
				0%
				0%
				0%
				0%
				0%
				0%
				0%
				0%
				0%
				0%
				0%
				0%
				0%
				0%

For peer review only

Staff time-use data collection tool. Adapted from "Haghparast-Bidgoli, Skordis J et al

Name
Position/role

Time period (e.g. Sep 2019 or Sep-Dec 2019)*	**Non-NeoTree activities (%)**	**NeoTree activities (%)**	Check- should be 100%	Data Entry (Admission and Discharges/Deaths)
			0%	
			0%	
			0%	
			0%	
			0%	
			0%	
			0%	
			0%	
			0%	
			0%	
			0%	
			0%	
			0%	
			0%	
			0%	
			0%	
			0%	
			0%	
			0%	
			0%	
			0%	
			0%	
			0%	
			0%	
			0%	
			0%	
			0%	
			0%	
			0%	

For peer review only

: Protocol for the cost-consequence and equity impact analyses of a cluster randomised c

N

Data back-up	M&M dashboard preparation	Research Meetings	Any other meetings relating to NeoTree	NeoTree Support/Testing/Pilot/Roll-out	NeoTree data pipeline

For peer review only

:ontrolled trial comparing three variants of a nutrition-sensitive agricultural extension interv

\eoTree activities (%)

Diagnosis Data Entry	Data Quality Checks/Data audits/Data cleaning	Research (paper writing, etc)	Software development	MOH Database integration	Training

For peer review only

ention to improve maternal and child dietary diversity and nutritional status in rural Odisha, India (

Clinical validation	Joint/admin	Check (should be 100%)
		0%
		0%
		0%
		0%
		0%
		0%
		0%
		0%
		0%
		0%
		0%
		0%
		0%
		0%
		0%
		0%
		0%
		0%
		0%
		0%
		0%
		0%
		0%
		0%
		0%
		0%
		0%
		0%
		0%

For peer review only

(UPAVAN trial) Trials (2019) 20:287"

For peer review only

Supplementary File 6

Data Management Procedures

When a baby is admitted, an admission form is completed on the NeoTree tablet app and an ID for the neonate is generated. The NeoTree ID generated is stored in the nurses' routine admission book along with the mother's name, acting as the data spine. Personal identifiable information (PII) is collected as part of the electronic admission form (incl. mother's name, baby's name, baby's date of birth). All data (including PII) are stored temporarily in an encrypted format on the tablet and hidden from view: only members of the research/clinical team can retrieve these data from the tablet by supplying a valid password. The identifiable NeoTree forms will be printed and inserted into clinical paper records to replace usual hand-written forms. All PII fields (e.g. mother's name, baby's name are not exported from the tablet into the NeoTree backend. PII fields are only stored in the tablet in an encrypted format.

Any other forms (e.g. discharge forms, laboratory results forms) submitted on the app are recorded against the neonates "NeoTree ID". In addition, some PII may also be stored (e.g. the mother's name) on the tablet, although this will be password protected as above, and records will be routinely wiped from the tablet as an extra safeguard.

In Zimbabwe, non-PII data from all forms are saved to a secure database at a Zimbabwean data centre: the "Clinical database". This database will be locally owned, stored and managed. The NeoTree team will have access to the database in order to ensure that the process for moving data from tablets to database is successful, and to enable a copy of the anonymized data for research purposes. (See "Anonymised research database" below.) Data moved from the tablets to the secure database will be encrypted in transit. Data are encrypted at rest in the Clinical database, where they are password protected. The only way to tie individual records in the database back to the neonate they describe is by matching the NeoTreeID with the PII, based on the paper print out stored in the patient's notes and the nurses' routine admission book. A regular process is in place to wipe data from the tablets, so encrypted identifiable data are stored for only a brief period of time (e.g. if a second copy of the print out is needed for clinical reasons). Aggregate statistics are computed on the data in the secure database, and served back to staff in the hospital via dashboards. A regular process will be put in place to copy anonymized data from the Clinical database into the "Anonymised research database", curated by the NeoTree research team and held on a UCL server. Data will be encrypted in transit and at rest. The entire approach to data security is twofold: (i) minimize the chance of data leakage, by ensuring that data is encrypted in transit and at rest, and by

For peer review only

minimizing the number of places the data is stored and (ii) pseudonomizing the data at the point of collection, so that even in the event of a breach, PII would not be leaked.

In Malawi, the process is identical except that the “clinical database” is stored on a secure virtual machine in an encrypted format using Amazon Webserver (AWS).

For peer review only

Supplementary File 7

Summary of implementation and research plan adaptation in response to COVID-19

1. Data collection.

In 2020 we submitted ethics amendments to allow for the option of remote data collection via secure platforms such as Microsoft Teams, if face-to-face data generation was deemed is not feasible due to COVID-19 restrictions. In August- December 2020, researchers conducted 17 semi-structured interviews with healthcare professionals at Chinhoyi Provincial Hospital, Zimbabwe. Most were conducted via remote methods (Microsoft Teams) due to travel restrictions which prevented on-site visits. The remainder of data collection is likely to be generated in face-to-face settings, with enhanced safety measures in line with hospital protocols, such as physical distancing, increased ventilation and mask wearing.

Routine admission and discharge data collection via the NeoTree platform has continued uninterrupted.

2. Implementation

Roll out of some functionalities was delayed for example the roll-out of the data dashboards in Zimbabwe. Clinical processes have inevitably adapted, for example reduced rotas of staff and suspension of face-to-face morbidity and mortality meetings during periods of heightened case numbers.

Nevertheless the NeoTree has proved robust in the face of these challenges. We have been highly responsive and adaptive to the pandemic, ensuring the safety of staff (for example remote support to clinical teams) while ensuring the NeoTree system has maintained function, data capture, analysis and emergency decision support for HCWs.

After media reports of increased stillbirths at Sally Mugabe Central Hosspital (SMCH) associated with COVID-19 pandemic reductions in hospital access, the NeoTree was adapted in one week at SMH to capture maternal and stillbirth outcomes. A similar process has been approved at Kamuzu Central Hospital, Malawi.

For peer review only

BMJ Open

Protocol for an intervention development and pilot implementation evaluation study of an e-health solution to improve newborn care quality and survival in two low resource settings, Malawi and Zimbabwe: Neotree.

Journal:	*BMJ Open*
Manuscript ID	bmjopen-2021-056605.R1
Article Type:	Protocol
Date Submitted by the Author:	21-Feb-2022
Complete List of Authors:	Wilson, Emma; UCL Great Ormond Street Institute of Child Health Population Policy and Practice, Population, Policy and Practice Research and Teaching Department<br>Gannon, Hannah; UCL Great Ormond Street Institute of Child Health Population Policy and Practice, Population, Policy and Practice Research and Teaching Department<br>Chimhini, Gwen; University of Zimbabwe College of Health Sciences, Department of Paediatrics and Child Health<br>Fitzgerald, Felicity; University College London, Infection Immunity and Inflammation, Great Ormond Street Institute of Child Health<br>Khan, Nushrat; UCL Great Ormond Street Institute of Child Health Population Policy and Practice<br>Lorencatto, Fabiana; University College London Centre for Behaviour Change<br>Kesler, Erin; The Children's Hospital of Philadelphia<br>Nkhoma, Deliwe; Parent and Child Health Initiative<br>Chiyaka, Tarisai; Biomedical Research and Training Institute<br>Haghparast-Bidgoli, Hassan; University College London Institute for Global Health<br>Lakhanpaul, Monica; UCL Great Ormond Street Institute of Child Health Population Policy and Practice<br>Cortina Borja, Mario; UCL Great Ormond Street Institute of Child Health Population Policy and Practice<br>Stevenson, Alexander; Mbuya Nehanda Maternity Hospital<br>Crehan, Caroline; University College London, Institute of Child Health UCL-GOSH<br>Sassoon, Yali; Snowplow Analytics<br>Hull-Bailey, Tim; University College London, Population<br>Curtis, Kristina; University College London Centre for Behaviour Change<br>Chiume, Msandeni; Kamuzu Central Hospital, Department of Paediatrics<br>Chimhuya, Simbarashe; University of Zimbabwe, Unit of Child and Adolescent Health, Faculty of Medicine and Health Sciences,<br>Heys, Michelle; UCL Great Ormond Street Institute of Child Health Population Policy and Practice
**Primary Subject Heading**:	Global health

Secondary Subject Heading:	Paediatrics, Research methods, Health informatics
Keywords:	NEONATOLOGY, Health informatics < BIOTECHNOLOGY & BIOINFORMATICS, Quality in health care < HEALTH SERVICES ADMINISTRATION & MANAGEMENT

SCHOLARONE™ Manuscripts

TITLE: Protocol for an intervention development and pilot implementation evaluation study of an e-health solution to improve newborn care quality and survival in two low resource settings, Malawi and Zimbabwe: Neotree.

Emma Wilson
Population, Policy and Practice Research and Teaching Department, University College London Great Ormond Street Institute of Child Health, 30 Guilford Street, London WC1N 1EH, UK, e.k.wilson@ucl.ac.uk

Hannah Gannon
Population, Policy and Practice Research and Teaching Department, University College London Great Ormond Street Institute of Child Health, 30 Guilford Street, London WC1N 1EH, UK, h.gannon@ucl.ac.uk

Gwendoline Chimhini
Unit of Child and Adolescent Health, Faculty of Medicine and Health Science, University of Zimbabwe, P.O. Box A178, Avondale, Harare, Zimbabwe, gwenchimhini@gmail.com.

Felicity Fitzgerald
Infection, Immunity and Inflammation, University College London Great Ormond Street Institute of Child Health, 30 Guilford Street, London WC1N 1EH, UK, felicity.fitzgerald@ucl.ac.uk

Nushrat Khan
Population, Policy and Practice Research and Teaching Department, University College London Great Ormond Street Institute of Child Health, 30 Guilford Street, London WC1N 1EH, UK, nushrat.khan@ucl.ac.uk

Fabiana Lorencatto
Centre for Behaviour Change, University College London, London, UK, f.lorencatto@ucl.ac.uk

Erin Kesler
Children's Hospital of Philadelphia, 3401 Civic Center Blvd, Philadelphia, USA, keslerem@gmail.com

Deliwe Nkhoma
Parent and Child Health Initiative (PACHI), Lilongwe, Malawi, dbnkhoma@gmail.com

Tarisai Chiyaka
Biomedical Research and Training Institute (BRTI), 10 Seagrave Road Avondale, Harare, Zimbabwe, tariemc@gmail.com

Hassan Haghparast-Bidgoli
Institute for Global Health, University College London, 30 Guilford Street, London WC1N 1EH, UK, h.haghparast-bidgoli@ucl.ac.uk

Monica Lakhanpaul
Population, Policy and Practice Research and Teaching Department, University College London Great Ormond Street Institute of Child Health, 30 Guilford Street, London WC1N 1EH, UK, m.lakanpaul@ucl.ac.uk

Mario Cortina-Borja
Population, Policy and Practice Research and Teaching Department, University College London Great

For peer review only - http://bmjopen.bmj.com/site/about/guidelines.xhtml

Ormond Street Institute of Child Health, 30 Guilford Street, London WC1N 1EH, UK, m.cortina@ucl.ac.uk

Alexander G. Stevenson,
Mbuya Nehanda Maternity Hospital, Harare, Zimbabwe. Department of Paediatrics, University of Zimbabwe, Harare, Zimbabwe doctoralexstevenson@gmail.com

Caroline Crehan
Population, Policy and Practice, University College London Great Ormond Street Institute of Child Health, 30 Guilford Street, London WC1N 1EH, UK, c.crehan@ucl.ac.uk

Yali Sassoon
Snowplow Analytics, Floor 6, 17 Bevis Marks, London, EC3A 7LN, yali@snowplowanalytics.com

Tim Hull-Bailey
Population, Policy and Practice Research and Teaching Department, University College London Great Ormond Street Institute of Child Health, 30 Guilford Street, London WC1N 1EH, UK, t.hull-bailey@ucl.ac.uk

Kristina Curtis
Centre for Behaviour Change, University College London, UK, k.curtis@ucl.ac.uk

Msandeni Chiume
Department of Paediatrics, Kamuzu Central Hospital, Lilongwe, Malawi, msandeni@gmail.com

Simbarashe Chimhuya
Child and Adolescent Health Unit, Faculty of Medicine and Health Sciences, University of Zimbabwe, Harare, Zimbabwe, chimhuya.simbarashe@gmail.com

Michelle Heys*
Population, Policy and Practice Research and Teaching Department, University College London Great Ormond Street Institute of Child Health, 30 Guilford Street, London WC1N 1EH, UK, m.heys@ucl.ac.uk

*Corresponding author

For peer review only - http://bmjopen.bmj.com/site/about/guidelines.xhtml

ABSTRACT

Introduction

Every year 2.4 million deaths occur worldwide in babies younger than 28 days. Approximately 70% of these deaths occur in low resource settings because of failure to implement evidence-based interventions. Digital health technologies may offer an implementation solution. Since 2014 we have worked in Bangladesh, Malawi, Zimbabwe, and the UK to develop and pilot Neotree: an android app with accompanying data visualisation, linkage and export. Its low-cost hardware and state-of-the-art software are used to improve bedside postnatal care and to provide insights into population health trends, to impact wider policy and practice.

Methods and analysis

This is a mixed methods (i) intervention co-development and optimisation and (ii) pilot implementation evaluation (including economic evaluation) study. Neotree will be implemented in two hospitals in Zimbabwe, and one in Malawi. Over the two-year study period clinical and demographic newborn data will be collected via Neotree, in addition to behavioural science informed qualitative and quantitative implementation evaluation and measures of cost, newborn care quality and usability. Neotree clinical decision support algorithms will be optimised according to best available evidence and clinical validation studies.

Ethics and dissemination

This is a Wellcome Trust funded project (215742_Z_19_Z). Research ethics approvals have been obtained: Malawi College of Medicine Research and Ethics Committee (P.01/20/2909; P.02/19/2613); UCL (17123/001, 6681/001, 5019/004); Medical Research Council Zimbabwe (MRCZ/A/2570), BRTI and JREC institutional review boards (AP155/2020; JREC/327/19), Sally Mugabe Hospital Ethics Committee (071119/64; 250418/48). Results will be disseminated via academic publications and public and policy engagement activities. In this study, the care for an estimated 15,000 babies across three sites will be impacted.

For peer review only - http://bmjopen.bmj.com/site/about/guidelines.xhtml

Strengths and limitations of this study

- Mixed methods intervention co-development and pilot implementation underpinned by behavioural science frameworks will optimise acceptability, feasibility and usability of Neotree, and the implementation strategy for larger scale roll out.
- Piloting of quantitative and qualitative clinical, quality of care, process, and economic measures will allow for a robust protocol for larger scale evaluation
- Collecting case fatality rate data from five hospitals in low resource settings will ensure informed sample size calculations for larger scale evaluation.
- Clinical, quality of care and demographic data from an anticipated 15,000 sick and vulnerable babies will enable studies of patterns, causes and risk factors for mortality and key diagnoses (e.g., sepsis/ neonatal encephalopathy).
- Clinical and cost effectiveness data will not be possible through this study design

Word count: 3946

For peer review only

For peer review only - http://bmjopen.bmj.com/site/about/guidelines.xhtml

INTRODUCTION

Worldwide, 2.4 million children younger than 28 days die yearly, accounting for 44% of deaths in children under five.(1) Approximately 70% of newborn deaths are avoidable through the implementation of simple, evidence-based interventions.(2) Health systems strengthening and training in newborn care are key to saving newborn lives.(3-5) Implementation of evidence-based guidelines can be supported through provision of reliable data systems, clinical decision support tools and education.(6, 7) We are developing and robustly evaluating an integrated quality improvement system for hospital-based sick and vulnerable newborns: Neotree. Neotree combines evidence-based clinical guidelines with real-time newborn data collection, data visualisation and export and newborn education on one platform.(8) This tablet-based digital system is for use at the hospital bedside by healthcare professionals (HCP) with a range of skills and competencies (primarily nurses) supporting the care and treatment of newborns. Neotree development has followed standard software development and Medical Research Council (MRC) complex intervention development frameworks.(9)

Background work to Neotree

A literature review of HCP-led newborn interventions in low resource settings (LRS) identified gaps in successful implementation of proven interventions.(10) To address these gaps, we explored the concept and acceptability of digital data capture and clinical decision support via workshops with HCP in Bangladesh (n~15; 2014, unpublished) and developed a prototype app (2015). An editor platform was designed to allow a clinician to configure the data capture forms and Neotree-alpha was configured (2016). Next, a qualitative study was conducted with HCP in Zomba Central Hospital (ZCH), Malawi to understand barriers to delivering quality newborn care and to explore the potential for digital health interventions to mitigate these.(8) We selected ZCH as a Neotree co-investigator had previously piloted a clinical algorithm to support babies with respiratory distress at this site.(11) Neotree-beta minimal viable product 1 (MVP-1) was subsequently co-developed with Malawian HCP (*n*=46, 2016-17) in the same clinical setting.(8) Neotree-beta MVP-1 included data capture on admission, resuscitation clinical diagnostic and management support and associated educational material. Clinical management advice pages were designed and linked to HCP chosen diagnoses. Neotree was found to be acceptable, feasible and highly usable and the potential for electronic clinical audit data demonstrated.(8, 12) HCP reported improved perceived ability to deliver quality care.(8) Neotree implementation did not continue at Zomba Central Hospital due to lack of ongoing funding. Identified strategies to optimise implementation included the introduction of a technical support role (suggested title: Neotree Ambassador).

For peer review only - http://bmjopen.bmj.com/site/about/guidelines.xhtml

In November 2018, Neotree was introduced at Sally Mugabe Central Hospital Neonatal Unit, Harare, Zimbabwe, at the request of local clinical teams,(13) presenting an opportunity to co-develop and test Neotree in a doctor-led unit within a new LRS with unique health system challenges. Neotree Ambassador roles were included in deployment, discharge data capture was developed, and linkage between clinical and laboratory blood culture data was undertaken to improve management of neonatal sepsis. A second site was identified in Malawi - Kamuzu Central Hospital - for further development and piloting. Since May 2019 we have been further developing Neotree-beta MVP-2 data capture on admission and discharge at this site, co-developing a data dashboard prototype and assessing usability, acceptability, barriers to implementation, usage and feasibility of both of these functions, using behavioural science frameworks. The data dashboard prototype included a summary statistics page, an admission hypothermia page and monthly morbidity mortality statistics developed using PowerBi.

Following this work, our priority has been the ongoing development and pilot implementation evaluation of remaining functions of Neotree, to create Neotree-gamma. Neotree-gamma will include data capture (admission, discharge and laboratory); clinical decision support (resuscitation and non-resuscitation diagnosis and management); education; and data transfer to local teams, dashboards and national databases. As HCP complete the admission, they will receive prompts to respond appropriately to the data they have entered and manage patients according to evidence-based guidelines. Neotree works offline to synchronise with a network when available. It is a not-for-profit venture with open-source code and can be preconfigured to adapt to available resources (medication and technology) within the index facility.

Aims and Objectives

Our primary research question is:

- *Can an integrated digital quality improvement system be implemented and sustained in two hospitals in Zimbabwe and one hospital in Malawi to improve newborn care?*

Secondary research questions include:

- *What is the case-fatality rate of intervention hospital neonatal units before and after implementation of Neotree?*
- *What is the case-fatality rate of two matched control hospital neonatal units not implementing Neotree during this time period?*
- *Can a predictive algorithm be developed to improve identification of neonatal sepsis?*
- *What is the sensitivity/specificity of Neotree sepsis algorithm versus gold standard?*

For peer review only - http://bmjopen.bmj.com/site/about/guidelines.xhtml

Study aims and objectives are to:

1. Further develop, implement and evaluate Neotree at three intervention sites;
2. Collect outcome data for newborns admitted to two hospitals where Neotree is not being implemented;
3. Test the clinical validity of Neotree neonatal sepsis diagnostic algorithm against gold standard diagnosis;
4. Add data visualisation and linkage to Neotree functionality;
5. Develop and test proof of concept for communicating daily electronic health records (EHR) using Neotree.

METHODS AND ANALYSIS

I. Study design

This is a mixed methods intervention co-development, pilot implementation and economic evaluation study (which will run from 7th Oct 2019 to 6th April 2022). We will continue to follow the MRC complex intervention development framework.(14) Table 1 shows anticipated timeline and study overview.

Table 1: Intervention roll-out and study timelines

Months 1-4	Study set up	Protocol development, recruitment and securing necessary permissions/approvals.
Months 1-21	Ongoing embedding of Neotree into standard care and continuation of data collection in SMCH and KCH as part of studies (REF: P.02/19/2613)	
Months 5-24	Implementation of Neotree into standard clinical care at CPH	
Months 1-9 (or until sample size reached)	Clinical validation sub-study	Clinical validity of the diagnostic algorithms
Months 5-9	Ongoing data dashboard development and data linkage	Co-development and optimisation the dashboard via design/usability workshops
Months 6-21	Pilot implementation science evaluation	Qualitative studies with HCP (nurses, nursing students and doctors), hospital administration staff (senior doctors/nurses, managers), and parents/carers
Months 6-21	Collection of quality of newborn care measures	
Months 10-15	Testing concept of electronic record system	Configure and test linkage of Neotree data to the MoH electronic record system
Months 1-21	Economic evaluation/costings data	During all phases, cost and resource implications data will be collected and analysed.
Months 21-24	Data analysis and write up	

For peer review only - http://bmjopen.bmj.com/site/about/guidelines.xhtml

Setting

This is a two-country study in Malawi and Zimbabwe where, in 2019, the neonatal mortality rates were 19.8 and 25.9 per 1000 births respectively.(15) In Zimbabwe, Neotree will continue to be implemented at Sally Mugabe Central Hospital, the largest of three newborn tertiary care facilities in Zimbabwe, delivering 12,000 newborns annually, where the 100-cot nursery often runs at 120-130% capacity. Audit data show case fatality rates of 210 deaths per 1000 admissions.(13) Neotree will also be introduced to clinical processes at Chinhoyi Provincial Hospital, Zimbabwe - a provincial level hospital, delivering 4,500 newborns annually with audit data showing case fatality rates of 180 per 1000 admitted babies. Sally Mugabe Central Hospital is one of six central hospitals in Zimbabwe and delivers primarily doctor-led care. Chinhoyi Provincial Hospital is one of eight provincial hospitals in Zimbabwe and delivers predominantly nurse-led newborn care. In Malawi, Neotree will continue to be delivered at Kamuzu Central Hospital, one of four central hospitals in Malawi, delivering 4,500 newborns annually. The neonatal unit admits around 2600 babies per year (from both within and outside of the hospital) with a case fatality rate of 210 per 1000 admitted babies.

Study participants

Study participants include frontline and managerial staff involved in the delivery of newborn care (e.g., nurses, doctors, and nursing students) at the three implementation sites, and other key personnel and stakeholders such as hospital managers and parents/caregivers of newborns admitted to newborn care units. We estimate that we will recruit ~170 HCP and 30 parents/caregivers across three sites in a phased pilot evaluation of each new co-developed functionality of the Neotree system.

Routine clinical admission, discharge and microbiological data will be prospectively recorded on the Neotree system for all newborns admitted to newborn care units at the three intervention sites (~12,000 admitted babies).

Consent procedures

Informed written consent will be sought from participants who take part in semi-structured interviews and focus group discussions. Participants with low literacy levels may give verbal consent which will be audio recorded and witnessed. We will follow international and local precedent for collection of neonatal pseudonymised data for the purposes of epidemiological surveillance and service evaluation such as the neonatal UK/ Australia/ New Zealand Badger net system,(16) and the WHO-led District Health Information Software (DHISv2).(17) Hence, we will not obtain informed consent from guardians to enter patient level data. No novel data will be recorded beyond that usually documented for clinical management in these settings, and no new procedures performed.

For peer review only - http://bmjopen.bmj.com/site/about/guidelines.xhtml

II. Co-development and optimisation of the Neotree system

Data capture functions will be optimised and validated; data dashboards and clinical decision support functions will be fully developed; data linkage to Zimbabwean national EHR and aggregate data DHISv2 systems demonstrated; and daily EHR functionality will be piloted.

Data capture

A neonatal data dictionary will be developed in line with similar existing guidelines.(18, 19) It will define the types and formats of data captured in Neotree, including standard data definitions to make it accessible to all data users. This will be made publicly available alongside open source code on GitHub (https://github.com/Neotree/Neotree). Data capture forms for admission, discharge and laboratory will be refined according to usability feedback. Data quality will be reviewed monthly to assure completeness and consistency of data by monitoring and reducing missing data where applicable. Annual prospective paper audits comparing Neotree capture of admissions and discharges to those recorded by ward paper records will be conducted. Data pipeline will be optimised to ensure automated data processing aligned with clinicians' needs and secure backup in UCL research database.

Dashboard development

The prototype initially developed at Kamuzu Central Hospital will be further developed in design/usability workshops with end-users. In both countries, one-to-one 'in vitro' user-tests will be conducted using a think aloud approach and an adapted user experience topic guide. During these sessions, pages of the dashboard (MVP-1) will be presented to HCP to gauge understanding and interpretation of the visualisations and collect feedback. Usability themes will be generated and analysed using agile rapid analysis. Following one-to-one user tests, participants will be invited to attend a videoed group workshop to consider new visualisations for MVP-2-dashboard. Optimal dashboard software will be identified.

Clinical decision support

Clinical decision support will be refined according to best available evidence and operationalised within the system. With Neotree, we will have a context-specific, detailed stream of clinical data at admission and outcome (discharge/death), in combination with laboratory diagnostic data for neonatal sepsis. With these data, we will build models to predict which babies are likely to benefit from specific interventions such as antibiotics. Clinical validation of the sepsis algorithm will be conducted retrospectively using data collected from Sally Mugabe and Kamuzu Central Hospitals. Admission diagnoses from the admitting healthcare professional, the senior clinician (based on

For peer review only - http://bmjopen.bmj.com/site/about/guidelines.xhtml

admission data alone) and blood culture results (gold standard) will be retrospectively compared with Neotree algorithm diagnosis. Assuming sensitivity and specificity of 92% (lower 95% CI: 84%) >222 babies would need to be diagnosed with blood culture positive sepsis over five months, during which more than 2000 babies will be admitted with sepsis across sites.(20)

Data linkage

We will develop and demonstrate the ability for Neotree data to be exported to the Zimbabwean national EHR system and to DHISv2, the most commonly used aggregate data system for reporting of health service data in the African region.

Daily electronic health record

A scoping study of the potential to extend Neotree-gamma to include daily EHR will be conducted (*n*~20 babies).

III. Implementation evaluation

We will conduct qualitative studies to assess the acceptability and feasibility of the functionalities of Neotree, informed by behaviour science theories and frameworks. Qualitative and quantitative usability data will be gathered in addition to quantitative measures of usage, patterns in clinical outcomes and measures of quality newborn care.

Acceptability, feasibility and usability

Focus groups and individual interviews will be conducted with HCP, after each new functionality becomes embedded within clinical practice at three timepoints: pre-implementation (Chinhoyi Provincial Hospital only), implementation and sustainability.

In the pre-implementation phase (at baseline), we will explore current practice, quality improvement needs, and potential barriers and enablers to implementation at the new site of Chinhoyi Provincial Hospital, Zimbabwe. We will hold one focus group discussion with HCP (*n*~10) and approximately ten semi-structured interviews with senior doctors, nurses and hospital administrators.

In the implementation phase, we will deploy Neotree to Chinhoyi Provincial Hospital while continuing to test and co-develop new functionalities across all sites. We will conduct three rounds of focus group discussions at each site (nine in total) with approximately ten participants in each (*n*~90). Perceived acceptability, feasibility and usability will be explored as follows:

- Round 1: Basic functionality of Neotree-beta MVP-2 (data capture at the bedside, clinical decision support for resuscitation and stabilisation and education) – Sally Mugabe Central and Chinhoyi Provincial Hospitals only.

For peer review only - http://bmjopen.bmj.com/site/about/guidelines.xhtml

- Round 2: Data dashboards (all sites)
- Round 3: Non-resuscitation clinical decision support (all sites)

To complement these data, we will conduct one set of individual interviews with approximately five senior clinical and managerial staff at each site (*n*~15). Topic guides for both focus groups and individual interviews will be semi-structured. Questions to explore acceptability of Neotree will be based on the domains from the Theoretical Framework of Intervention Acceptability(21) e.g. burden, intervention coherence, opportunity costs, ethicality. Questions to explore barriers and enablers to implementing Neotree in practice will be based on the Theoretical Domains Framework;(22) an integrative framework of 33 behaviour change theories proposing 14 domains of factors facilitating/hindering behaviour change e.g. knowledge, available resources, social influences, motivation, etc. Drafts of topic guides (Supplementary Files 1-3) will be reviewed and piloted before the final versions are implemented. Focus groups and interviews will last for a maximum of two hours and one hour respectively. These will be conducted by a trained researcher, either face-to-face in a private location in the hospital, or remotely via platforms such as Microsoft Teams, at a convenient date and time to participants.

Focus groups and interviews will be audio-recorded, transcribed verbatim, and fully anonymised. Anonymised data will be stored securely at UCL for 10 years. We will analyse the transcripts using a combined deductive framework and inductive thematic analytical approach,(23) to identify which domains are key influences on implementation and acceptability. To ensure reliability, a subset of 10% of transcripts will be double-coded by another researcher (KC). We will compare themes over time (i.e., across the implementation period), across countries, and according to professional role. Refreshments will be provided to those taking part and travel costs will be reimbursed where relevant. Following analyses of focus group and interview data, we will identify any potential refinements or additions to be made to Neotree or the associated training materials, in order to improve acceptability, usability and address barriers and enablers to implementation. We will draw on behavioural science intervention development frameworks(24, 25) to identify relevant behaviour change techniques to address identified barriers and enablers.

In the sustainability phase, we will conduct a final round of data collection with ~10 healthcare professionals and ~5 senior clinical and managerial staff at each site (total *n*~45) with a focus on intervention sustainability once research and software development teams have withdrawn. No further changes to functionality will be made to Neotree-gamma during this period. We will compare themes from sustainability versus initial implementation data collection period, across countries and across professional roles using the same methodology described above.

For peer review only

We will interview ~10 parents/carers of newborns at each of the three hospitals (~30 parents/carers) to explore their views on the use of digital innovations in healthcare, and the perceived acceptability of Neotree. Interviews will be semi-structured, based on the Theoretical Framework of Acceptability.(21) Interviews will be conducted by an independent researcher, who is not involved in the provision of clinical care. Assurances will be given to parents/ caregivers that their participation will not affect the care of the baby or other family members. Findings will inform refinements of Neotree system and associated training to ensure it is acceptable both to those providing and receiving newborn care.

Analysis of routine health data

Admission data from intervention sites will be analysed monthly to estimate measures such as overall and disease specific case-fatality rate, quality of newborn care and usage. We will test the feasibility of collecting the quality of newborn care endpoints shown in Table 2, to inform the data collection procedures in the large-scale evaluation. Most map directly to WHO standards of quality of maternal and newborn care in health facilities.(5)

Table 2: Quality of newborn care endpoints

**Temperature on admission:**<br>1. The proportion of all newborns who had a normal body temperature (36.5-37.5 deg C) at the first complete examination (60-120 min after birth) [WHO quality statement 1.1b]
**Documentation:**<br>2. The proportion of all newborns for whom there is documented information on a) body temperature, b) respiratory rate, c) HR, d) O2 saturations in air e) saturations in oxygen f) blood sugar, feeding behaviour d) absence or presence of danger signs, e) admission weight [WHO quality statement 1.1c]
**Resuscitation:**<br>3. The proportion of all newborns who were not breathing spontaneously after additional stimulation at the health facility who were resuscitated with a bag-and-mask. [WHO quality statement 1.5]<br>4. The proportion of all newborns who were not breathing spontaneously after additional stimulation at<br>the health facility who were resuscitated with a bag-and-mask within 1 min of birth. [WHO quality statement 1.5]
**Infection:**<br>5. The proportion of all newborns in the health facility ***with signs of infection*** who received injectable antibiotics. [WHO quality statement 1.7b]<br>6. The proportion of all newborns ***of mothers with signs of infection*** in the health facility who received injectable antibiotics. [WHO quality statement 1.7b]<br>7. Proportion of newborns ***with suspected severe bacterial infection*** who received appropriate antibiotic therapy. [WHO quality statement 1.8]<br>8. Proportion of newborns born to HIV +ve mothers who received Nevirapine on first day of life [discharge]<br>9. Proportion of neonates with low blood sugar who are treated with a feed or dextrose as appropriate.
**Data collection & health system:**<br>10. The proportion of all newborns currently in the health facility who have a patient identifier and individual clinical medical record*. [WHO quality statement 2.1]

For peer review only - http://bmjopen.bmj.com/site/about/guidelines.xhtml

11. The proportion of all newborns discharged from the health facility within the past 24 h who had an accurately completed record of processes of care, treatments, outcomes and **diagnoses** (with ICD code). [WHO quality statement 2.1]
12. Data are collected routinely in the health facility during labour, childbirth and the postnatal period (and used regularly to make decisions on quality improvement)
13. The proportion of newborns seen in the health facility in the past three months who fulfilled the facility's criteria for referral who were actually referred. [WHO quality standard 3.1]

Economic Evaluation-intervention costing

During all phases, cost and resource implication data will be collected and analysed to determine to costs of developing and piloting Neotree from the provider perspective These include Neotree development costs, training, planning and set up, implementation, and resource implications for the hospital/healthcare system. Costs of developing and implementing the Neotree system will be collected through expenditure reports, time-use survey and interviews with the project staff (Supplementary Files 4-5). Information on potential impact on intervention hospitals will be collected through time-use surveys with all HCP involved in Neotree development and implementation, supplemented with project records on their involvement in different intervention activities, such as software development and training workshops (i.e., opportunity costs). To measure the effect of implementation on time spent on procedures/activities in the delivery of newborn care pathways, a pilot time-use survey will be conducted (implementation phase) at all intervention sites and one comparable site in each setting, to record admission and discharge activities and time spent on each activity for around 10 newborn patients in each hospital (*n*=30 in total). The tools developed and used for economic data collection will be modified and adopted for use in a larger trial/evaluation study of Neotree.

IV. Protocol for future large scale evaluation

Data from all phases will inform the study protocol, costings and implementation strategy for a future large-scale roll-out and evaluation.

To inform the sample size calculation for a large-scale evaluation (e.g., a stepped wedge trial) we will collect 6 months of clinical, morbidity and mortality data from newborns admitted to two comparator hospitals providing usual care in Zimbabwe. These are: Mbuya Nehanda Maternity Hospital (part of the Parirenyatwa hospital group) and Bindura Provincial Hospital. Selection of comparator hospitals is based on geographic proximity and similarities in catchment area and service level provision to intervention hospitals Data from paper-based admission forms will be entered retrospectively at these sites onto the Neotree app. In Malawi, aggregate electronic routine case fatality data from representative health facilities will be attained from the Health Management

For peer review only

For peer review only - http://bmjopen.bmj.com/site/about/guidelines.xhtml

Information System (i.e., from a central hospital, with nurse-led provision and a similar catchment area to Kamuzu Central Hospital).

Of note, data collective during this pilot implementation phase are insufficient and not designed to test clinical and cost effectiveness – rather they will be used to refine Neotree and to inform a robust evaluation. Primary, secondary, process, quality of care and economic outcomes will be clarified, alongside data collection procedures and our logic model.

V. Patient and public involvement

No patients were directly involved in the design of the study. However, this protocol responds to findings from our pilot acceptability and feasibility work in Malawi in 2016, which indicated that HCP were concerned about parent perceptions of the use of Neotree in routine newborn care.(8)They reported that some parents were "*afraid they think you are taking the information someplace else*". Student-nurses reported that parents/guardians thought the tablets were a distraction and that "*we are just on social networks*", while others viewed it positively "*with gladness*".(8, 26)

In response, this study will explore the acceptability of Neotree via semi-structured interviews with parents/carers across the three implementation sites, to capture patient perspectives. We will also develop a patient and public involvement strategy. This will include working with partners (Art & Global Health Center Africa and the UCL Co-Production Collective) to build the capacity of mothers/carers so that they may support dissemination of study results in catchment areas of intervention hospitals, and co-produce future iterations of Neotree. We will use participatory methods, such as community dialogues, to disseminate study results.

VI. Data management plan

Study protocols, training manuals and data collection tools will be made available on a study website. Upon study completion, all documents and record forms will be stored onsite for at least 10 years. Our team has had extensive discussions with clinical teams and Ministries of Health in setting up this study. Our agreed data management plan for newborn data is in Supplementary File 6. A subset of the anonymised research database will be made open source after publication of the main study findings to ensure maximum reach and accessibility.

Focus groups, semi-structured interviews and workshops will be recorded on two digital audio recording devices. Recordings will be anonymised and transcribed verbatim using password-protected laptops. Hard copies of field notes and transcripts will be anonymised and locked away. Soft anonymised copies will be stored on a secure laptop. Similarly, data collected through time-use surveys will be anonymized and stored on a secure laptop.

For peer review only - http://bmjopen.bmj.com/site/about/guidelines.xhtml

ETHICS AND DISSEMINATION

Research ethics approvals for this study were granted from: Malawi College of Medicine Research and Ethics Committee (P.01/20/2909; P.02/19/2613); University College London (17123/001; 6681/001; 17123/001; 5019/004); Medical Research Council Zimbabwe (MRCZ/A/2570); Biomedical Research Training Institute institutional review board, Zimbabwe (AP155/2020), Joint Research Ethics Committee, University of Zimbabwe review board (JREC/327/19), and Sally Mugabe Hospital Ethics Committee (HCHEC 250418/48; 071119/64).

Potential ethical considerations

There are no conflicts of interest. No new drugs/biologic agents will be administered to the participants during this study, nor will previously used agents be used in a new manner. No additional tests of clinical management will be initiated beyond those of standard practice. Neotree will support the consistent implementation of national/international evidence-based guidelines.

Dissemination

Our output management plan - co-developed with our partners and co-investigators - will be reviewed every three months. Discussions with the Zimbabwean Ministry of Health are ongoing about how the Neotree could, if successful, be rolled out across the country. We will develop long-term data sharing and access procedures (including a Data Access Committee with independent academic and lay members to assess requests for aggregated data).

A stakeholder event (Zimbabwe) will focus on synergies in research needs between Malawi and Zimbabwe, using Neotree as a platform for African-driven research questions, projects and higher degrees. It will include presentations on Quality Improvement projects/audits completed using Neotree. An anonymised research database comprising clinical newborn data from all three hospital sites will be designed at the Neotree collaborator meeting with a data management plan setting out conditions for access according to national/international guidelines. We will disseminate our discussions/conclusions in an opinion piece in a peer-reviewed journal.

IMPACT OF COVID-19

Our research programme has continued throughout the COVID-19 pandemic.(27) Key changes to our research plan are described in Supplementary File 7.

DISCUSSION

For peer review only - http://bmjopen.bmj.com/site/about/guidelines.xhtml

Few digital interventions in LRS have been well described and rigorously evaluated thus limiting their potential with respect to clinical and implementation effectiveness, sustainability and generalisability. The work described here will allow the completion of co-development of Neotree-gamma with key functionalities configured, operationalised, tested and ready for larger scale roll out and evaluation across Zimbabwe and Malawi. Behavioural science theory and frameworks will be used to explore barriers/enablers to implementation and inform intervention refinement and development of strategies to encourage implementation on scale and sustainability longer term.(21-23) The economic evaluation will estimate pilot implementation costs and resource requirements for sustainability and scale up, and affordability. Clinical outcome and cost data will inform sample size and potential resources for a large scale evaluated roll out. It should be acknowledged that the costs and resource use at the pilot hospitals will not necessarily generalisable to other hospitals, but we hope that the detailed cost analysis alongside the pilot, will provide information to estimate potential costs at scale.

Our overall vision is to use evidenced-based best practice and information technology to improve clinical decisions for newborn care and increase rates of newborn survival in under-resourced health care settings. We aim to create a locally led and curated database of aggregate newborn outcomes that can be used to robustly evaluate health systems, undertake healthcare planning and resource allocation at both the micro level (by site), regional, national and international levels. In addition, we are committed to open-source code ensuring the Neotree code is freely available for countries and hospitals to adopt, modify and run, and enabling those hospitals and ministries to own and control their data.

This work addresses a primary sustainable development goal - reducing neonatal mortality in low resource settings - and addresses key strategic aims of the Every Newborn Action Plan.(28) Our clinical algorithms and linkage to microbiology data aim to optimise the management of leading causes of newborn death. In this study, the care for an estimated 15,000 babies across the three test sites will be impacted by Neotree. Through successful rollout across Zimbabwe and Malawi - the care for nearly 300,000 babies could be improved annually.

For peer review only

Authors' contributions: MH and EW wrote the first draft of the protocol paper with all other co-authors inputting to content and approving the final draft. MH, EW and HG led the writing of the research ethics applications. MH designed the study methods with guidance and input from all co-authors. Co-authors also offered specific input and expertise as follows: FL, EW and KC on behavioural sciences methodology; FF, HG, AS, SC, GC, CC, DN, TC, THB, EK and MCB on clinical validation and implementation; MCB on quantitative analyses; ML on parent/carer qualitative study and public engagement; CC on usability testing, data dashboard development and assessment of impact on quality newborn care; YS, THB and NK on software development and data pipeline; and HHB on economic evaluation.

Funding statement: This work was supported by Wellcome Trust grant number 215742_Z_19_Z.

Competing interests' statement: MH, YS, EK and FF are trustees of the Neotree charity (www.neotree.org) but receive no financial payment from this role. CC was a trustee of the Neotree charity until 2018 and received no financial payment for this role. There are no other conflicts of interest to declare from any other co-author.

For peer review only

References

1. Liu L, Oza S, Hogan D, et al. Global, regional, and national causes of child mortality in 2000-13, with projections to inform post-2015 priorities: an updated systematic analysis. *Lancet (London, England)*. 2015;385(9966):430-40.
2. Knippenberg R, Lawn JE, Darmstadt GL, et al. Systematic scaling up of neonatal care in countries. *Lancet (London, England)*. 2005;365(9464):1087-98.
3. Lawn JE, Kinney MV, Black RE, et al. Newborn survival: a multi-country analysis of a decade of change. *Health Policy Plan*. 2012;27 Suppl 3:iii6-28.
4. Moxon SG, Lawn JE, Dickson KE, et al. Inpatient care of small and sick newborns: a multi-country analysis of health system bottlenecks and potential solutions. *BMC Pregnancy and Childbirth*. 2015;15(2):S7.
5. World Health Organisation. Standards for improving quality of maternal and newborn care in health facilities. Geneva WHO; 2016. Available from: https://apps.who.int/iris/bitstream/handle/10665/249155/9789241511216-eng.pdf;jsessionid=74464E5C8BD4821F8C1B93584988EEE0?sequence=1.
6. Agarwal S, Labrique A. Newborn health on the line: the potential mHealth applications. *Jama*. 2014;312(3):229-30.
7. Li Y-p, Fang L-q, Gao S-q, et al. Decision Support System for the Response to Infectious Disease Emergencies Based on WebGIS and Mobile Services in China. *PLOS ONE*. 2013;8(1):e54842.
8. Crehan C, Kesler E, Nambiar B, et al. The NeoTree application: developing an integrated mHealth solution to improve quality of newborn care and survival in a district hospital in Malawi. *BMJ Glob Health*. 2019;4(1):e000860.
9. Campbell M, Fitzpatrick R, Haines A, et al. Framework for design and evaluation of complex interventions to improve health. *Bmj*. 2000;321(7262):694-6.
10. Kesler E, Costello A, Heys M, et al. G259(P) A systematic review of health worker-led interventions to reduce mortality in low birth weight neonates in low and middle-income institutional settings. *Archives of Disease in Childhood*. 2015;100(Suppl 3):A112.
11. Crehan C, Colbourn T, Heys M, et al. Evaluation of 'TRY': an algorithm for neonatal continuous positive airways pressure in low-income settings. *Arch Dis Child*. 2018;103(8):732-8.
12. Crehan C, Kesler E, Chikomoni IA, et al. Admissions to a Low-Resource Neonatal Unit in Malawi Using a Mobile App: Digital Perinatal Outcome Audit. *JMIR Mhealth Uhealth*. 2020;8(10):e16485.
13. Gannon H, Chimhuya S, Chimhini G, et al. Electronic application to improve management of infections in low-income neonatal units: pilot implementation of the NeoTree beta app in a public sector hospital in Zimbabwe. *BMJ Open Quality*. 2021;10(1):e001043.
14. Craig P, Dieppe P, Macintyre S, et al. Developing and evaluating complex interventions: the new Medical Research Council guidance. *Bmj*. 2008;337:a1655.
15. The United Nations Inter-agency Group for Child Mortality Estimation (UN IGME). Available from: https://data.unicef.org/topic/child-survival/neonatal-mortality/
16. https://www.clevermed.com/badgernet/badgernet-neonatal [
17. https://www.dhis2.org/ [
18. https://public.vtoxford.org/ [
19. Imperial College London Neonatal dataset. Available from: https://www.imperial.ac.uk/media/imperial-college/medicine/dept-medicine/infectious-diseases/neonatology/Neonatal-dataset-ISB1595-release-1-version-22.pdf
20. Chu H, Cole SR. Sample size calculation using exact methods in diagnostic test studies. *J Clin Epidemiol*. 2007;60(11):1201-2; author reply 2.
21. Sekhon M, Cartwright M, Francis JJ. Acceptability of healthcare interventions: an overview of reviews and development of a theoretical framework. *BMC Health Serv Res*. 2017;17(1):88.

For peer review only

For peer review only - http://bmjopen.bmj.com/site/about/guidelines.xhtml

22. Francis JJ, O'Connor D, Curran J. Theories of behaviour change synthesised into a set of theoretical groupings: introducing a thematic series on the theoretical domains framework. *Implement Sci*. 2012;7:35.
23. Atkins L, Francis J, Islam R, et al. A guide to using the Theoretical Domains Framework of behaviour change to investigate implementation problems. *Implement Sci*. 2017;12(1):77.
24. Michie S, Richardson M, Johnston M, et al. The behavior change technique taxonomy (v1) of 93 hierarchically clustered techniques: building an international consensus for the reporting of behavior change interventions. *Ann Behav Med*. 2013;46(1):81-95.
25. Michie S, van Stralen MM, West R. The behaviour change wheel: a new method for characterising and designing behaviour change interventions. *Implement Sci*. 2011;6:42.
26. Crehan C, Huq T, Kesler E, et al. 073 A qualitative study of healthcare workers perceived barriers and facilitators to neonatal care in a central hospital in malawi. *Archives of Disease in Childhood*. 2018;103(Suppl 2):A30-A.
27. Chimhuya S, Neal SR, Chimhini G, et al. Indirect impacts of the COVID-19 pandemic at two tertiary neonatal units in Zimbabwe and Malawi: an interrupted time series analysis. *medRxiv*. 2021:2021.01.06.21249322.
28. Kinney MV, Cocoman O, Dickson KE, et al. Implementation of the Every Newborn Action Plan: Progress and lessons learned. *Semin Perinatol*. 2015;39(5):326-37.

For peer review only

For peer review only - http://bmjopen.bmj.com/site/about/guidelines.xhtml

Supplementary File 1

Draft Focus Group Discussion (FGD) and Semi-Structured Interview Topic Guides with Healthcare Professionals (nurses and doctors)

Topic Guide 1: Baseline SSI

Interview guide for NeoTree semi-structured interview

Aim: to explore practice pre-implementation

Timing: pre-implementation (months 5-6)

Participants: Healthcare Professionals

Version: 1.0

Introduction

Hello, thank you very much for taking the time to speak to me today. My name is ______ and I work for _____________. This interview will probably last around 1 hour. As a reminder, I am talking to you today, as I work for a study that aims to improve the care given to sick and vulnerable babies in hospitals.

Before we start, can I check whether you have:

- been told about the study
- had an information sheet
- signed a consent form
- agreed to audio recording of the discussion

At this point, do you have any questions about the purpose of the study, or the documents you've been given [e.g. PIS/ consent form]? Is there anything that isn't clear?

I want to reassure you that I work for a research organisation [name] and not [name of health facility]. Anything you tell me today is confidential and will not be shared with your colleagues. All personal and identifying information (such as your name/names of others) mentioned will be removed and replaced with a code.

I just want to remind you that your participation in this interview is entirely voluntary [i.e. it is your choice]. If you do not want to answer a question you can just say 'pass' and we will move on to the next question.

I am interested to understand your role at [health facility] in the provision of neonatal care and how care is delivered. There are no right or wrong answers to these questions; I am just interested in your views so please answer honestly.

If you want to take a break or stop at any point, please tell me. And if you wish to withdraw from the study you are completely free to do so at any point.

For peer review only

1) Warm up questions

   a) What is your role on the unit? How long have you worked here? Where did you complete your training?

2) Staffing

   a) How many nurses, midwives, student nurses are on the unit, what qualifications, what are their roles?
   b) Is there a paediatrician in the unit? If not can you elaborate on the doctors' support you receive in the unit?
   c) What professional training is offered to nurse/ doctors?

3) Services and Procedures

   a) What health care services are provided in the neonatal ward? How linked with maternity ward?
   b) What are existing roles/ responsibilities/ practices for neonatal care? (from admission to discharge, i.e. who does what? when? where?)

4) Pathways and communication

   a) Can you describe the pathways for referrals into and out of the neonatal unit? How well do referral pathways work in practice? What could be improved?
   b) How would you describe your relationship with your co-workers, doctors, management? Any challenges in team working/ communication?
   c) How would you describe morale among HCWs on the unit? What could be improved?

5) MIS

   a) How are patient data captured currently (admission/ discharge/ lab results)? Any challenges with data capture?
   b) How are data used for quality improvement?

6) Equipment and supplies

   a) What machinery is available in the unit?
   b) What lab facilities are there? Drug supplies and consumables?
   c) What equipment/ supplies do you feel are lacking? Any problems with procurement?

7) Challenges in provision of neonatal care

   a) What do you feel are the main challenges to providing neonatal care in this hospital?
   b) How do you feel neonatal services could be improved?

8) Closing remarks

   a) Is there anything you would like to ask me?

For peer review only

Thank you so much, we really appreciate the time you've taken to participate in this study.

For peer review only

Topic Guide 2: Baseline FGD

Topic guide for NeoTree focus groups based on Theoretical Framework of Acceptability (TFA) and the Theoretical Domains Framework (TDF)

Timing of focus group: Pre-implementation (months 5-6)

Participants: Healthcare Professionals

Version: 1.0

Introduction

Hello, thank you very much for taking the time to speak to me today. My name is _______ and I work for _______________. This discussion will probably between 1-2 hours. As a reminder, I am talking to you today, as I work for a study that aims to improve the care given to sick and vulnerable babies in hospitals.

Before we start, can I check whether you have:

- been told about the study
- had an information sheet
- signed a consent form
- agreed to audio recording of the discussion

At this point, do you have any questions about the purpose of the study, or the documents you've been given [e.g. PIS/ consent form]? Is there anything that isn't clear?

I just want to remind you that your participation in this discussion is entirely voluntary [i.e. it is your choice].

I am interested in your views about the implementation of the NeoTree at [health facility]. Your opinions as health care workers at the frontline of newborn care are very important to us. There are no right or wrong answers to these questions, just differing points of views. Please feel free to share your point of view even if it differs from what others have said. Keep in mind that we are just as interested in negative comments as positive comments.

Please give everyone a chance to speak, and please do not share what we discuss today outside this group. Please say your ID number before you speak and do not say names during the discussion as we want to keep them anonymised.

If you want to take a break or stop at any point, please tell me. And if you wish to withdraw from the study you are completely free to do so at any point.

Before we start, do you have any questions for me?

For peer review only

1) Warm up questions

What is your role at this hospital? How long have you worked here?

2) Introduction to NeoTree [demonstration of the NeoTree]

Today I'd like us to discuss the NeoTree, which is a digital health intervention that we are planning to introduce at [health facility] to improve the quality of newborn care. The NeoTree is an app. It allows you to enter in all the clinical information about the baby on admission and discharge. It gives you guidance on what is likely to be wrong with the baby and how to best treat and manage the baby, based on international guidelines. Whilst the baby is on the newborn care unit it will also provide an electronic linkage to any tests that are done to check for infection. The NeoTree also stores the clinical information for all of the babies and then feeds back to healthcare workers and the hospital on things like how many babies were admitted each month and what was wrong with them so that they can best plan the services needed.

Do you have any questions?

3) Knowledge & Skills (TDF)

a) How easy or difficult do you think it would be to use the NeoTree at [health facility]?

Prompt: What in particular would be easy? What in particular would be difficult?

b) Do you think you would need any additional skills or training to use NeoTree?

4) Beliefs about consequences (TDF)

a) What do you perceive to be the benefits/positives of using NeoTree?

Prompt: for: you as nurses/ doctors, other colleagues/roles and for patients & families?

b) Would there be any drawbacks?

Prompt: for you as nurses/ doctors, other colleagues/roles and for patients and families?

c) Overall, do you think the pros would outweigh the cons?

5) Burden (TFA) + Social Professional Role/Identity (TDF)

a) Do you think using the NeoTree would create extra work for you? In what way?

b) Do you feel it is would be your responsibility to use a tool like this? Who else might be responsible for completing the information in NeoTree or using it to guide decisions around care?

6) Social influences (TDF)

a) To what extent would the views of others influence whether you use the NeoTree?

For example the opinions of other nurses in the unit, other doctors, senior hospital managers, the NeoTree project management team, parents/guardians?

7) Opportunity costs (TFA)

Is there anything that you feel you would have to give up in order to use the NeoTree?

8) Ethicality (TFA)

a) Do you feel the NeoTree would be safe? For you and for babies?

b) Do you feel introducing the NeoTree would be fair?

9) Self-efficacy (TFA) & Beliefs about capabilities (TDF)

a) How confident would you be using the NeoTree? Is there anything in particular you would not be confident about?

b) Is there anything that could help you become more confident?

10) Environmental context and resources (TDF)

a) Do you think you would have sufficient resources to implement the NeoTree?

11) Goals (TDF)

a) Compared to other things you have to do, where does introducing the NeoTree fit in in terms of a priority? What would be the competing priorities?

b) Do you have any targets/goals for practice? How would the NeoTree fit in with that if at all?

12) Perceived effectiveness (TFA) + optimism (TDF)

In your view, how likely is the NeoTree to improve quality of newborn care?

13) Intentions (TDF)

a) Would you like to use the NeoTree? Can you explain your reasons why/why not?

b) To what extent do you intend to use it?

14) Barriers/ facilitators (general)

a) What do you think might be some of the barriers to introducing the NeoTree at [health facility]?

b) Is there anything that might make it easier to implement the NeoTree at [health facility]?

To summarise, the main points from our discussion are (facilitator to summarise key points). Is there anything else you'd like to add? Is there anything you'd like to ask me?

Thank you so much. We really appreciate the time you've taken to participate in this study.

For peer review only

Topic Guide 3: FGD - basic functionality of the NeoTree

Topic guide for NeoTree focus groups based on Theoretical Framework of Acceptability (TFA) and the Theoretical Domains framework (TDF)

Target behaviours – digital documentation on NeoTree, following emergency diagnostic guidelines, receiving educational support

Timing of focus group: implementation phase (months 6/7)

Participants: Healthcare Professionals

Version 1.0

Introduction

Hello, thank you very much for taking the time to speak to me today. My name is ______ and I work for _____________. This discussion will probably between 1-2 hours. As a reminder, I am talking to you today, as I work for a study that aims to improve the care given to sick and vulnerable babies in hospitals.

Before we start, can I check whether you have:

- been told about the study
- had an information sheet
- signed a consent form
- agreed to audio recording of the discussion

At this point, do you have any questions about the purpose of the study, or the documents you've been given [e.g. PIS/ consent form]? Is there anything that isn't clear?

I just want to remind you that your participation in this discussion is entirely voluntary [i.e. it is your choice].

I am interested in your views about the implementation of the NeoTree at [health facility]. Your opinions as health care workers at the frontline of newborn care are very important to us. There are no right or wrong answers to these questions, just differing points of views. Please feel free to share your point of view even if it differs from what others have said. Keep in mind that we're just as interested in negative comments as positive comments

Please give everyone a chance to speak, and please do not share what we discuss today outside this group. Please say your ID number before you speak and do not say names during the discussion as we want to keep them anonymised.

If you want to take a break or stop at any point, please tell me. And if you wish to withdraw from the study you are completely free to do so at any point.

Before we start, do you have any questions for me?

1) Warm up questions

What is your position at this hospital? How long have you worked here?

For peer review only

2) Intervention coherence (TFA)

We are going to ask you a few questions about NeoTree which as you know is a new digital platform we have started using on the ward.

a) Can you talk me through your understanding of what NeoTree is? What it is for?

Optional Prompt: What are the functions / objectives of the NeoTree?

3) Barriers (general)

a) What in general do you think are the barriers to using NeoTree in day to day practice?

b) Is there anything that made it easier or encouraged you to use NeoTree in day to day practice?

4) Knowledge & Skills (TDF)

a) Overall, 'how easy or difficult is it to use NeoTree?'

Prompt: What in particular is easy? What in particular is difficult?

b) Do you think you need any additional skills or training to use NeoTree?

5) Beliefs about consequences (TDF)

a) How does using NeoTree differ to the previous paper-based system for documentation?

b) Are there any benefits/positives of using NeoTree?

Prompt: for you as nurses, other colleagues/roles and for patients & families?

c) Are there any draw-backs/negatives?

Prompt: for you as nurses, other colleagues/roles and for patients and families?

d) Overall, do you think the pros outweigh the cons?

6) Burden (TFA)/ Beliefs about consequences

a) How does it affect your capacity to do your job? Does it help or hinder you to do your job?

b) How much effort does it require to use NeoTree compared to the paper form you were using before?

- does it require more time? more support from colleagues?

7) Affective attitude (TFA) + Emotion (TDF)

a) Do you like using NeoTree? What in particular do you like or dislike?

Prompt: Any concerns or worries about NeoTree?

For peer review only

8) Social influences (TDF)

a) To what extent do the views of others influence if and how you use the NeoTree? For example the opinions of other nurses in the unit, the NeoTree ambassador, doctors, the NeoTree project management team, parents/guardians?

b) Has the NeoTree changed team working and communication in the unit in any way?

Prompt: communication, roles, responsibilities?

9) Opportunity costs (TFA)

Is there anything that you feel you must give up in order to use the NeoTree?

10) Ethicality (TFA) Social/Professional role & identity (TDF)

a) Do you feel it is safe to use NeoTree? For you and for babies?

b) How does NeoTree fit in with your ways of working? Do you feel it is your responsibility to use a tool like this? Who else might be responsible for completing the information in NeoTree or using it to guide decisions around care?

11) Self-efficacy (TFA) Beliefs about capabilities (TDF)

a) How confident are you in using the NeoTree? Is there anything in particular you are not confident about?

b) Is there anything that could help you become more confident?

12) Environmental context and resources (TDF)

a) Do you have enough resources to use the NeoTree?

13) Memory, attention, decision making (TDF)

a) Do you use NeoTree to guide your decision-making? How so?

Does it make things easier/more difficult?

b) Have you ever forgotten to use the NeoTree?

If yes - in what kind of situations? and how can this be avoided in future?

c) Are there ever any instances when you decided to deviate from recommendations in NeoTree? Talk me through this....

d) Are there ever any instances where you decided not to use NeoTree? If so, can you talk me through this...

14) Goals (TDF)

a) How important is using NeoTree for you? And why?

b) Compared to other things you have to do, where does completing the NeoTree forms and using NeoTree fit in in terms of a priority? What are the competing priorities?

c) Do you have any targets/goals for practice? How does NeoTree fit in with that if at all etc

For peer review only

15)Perceived effectiveness (TFA) + TDF optimism

a) In your view, how likely is the NeoTree to improve quality of newborn care?

16)Reinforcement (TDF)

a) What positive experiences have you had with the NeoTree that would encourage you to use it again? Are there any negative experiences that would discourage you from using NeoTree again?

b) Are there any incentives / rewards / pressures to use NeoTree?

17)Intentions (TDF)

a) Do you intend to use the NeoTree in your day-to-day practice?

18)Behavioural regulation (TDF)

a) To what extent has using NeoTree become habitual or something you do without thinking, day to day? Why/how so?

b) Do you ever review the data you enter into NeoTree? Use it to monitor practice? Discuss it with colleagues?

c) Have you ever encountered any problems using NeoTree? How did you overcome these?

d) Is there anything you think we can do to improve the implementation of the NeoTree on the ward?

To summarise, the main points from our discussion are (facilitator to summarise key points). Is there anything else you'd like to add? Is there anything you'd like to ask me?

Thank you so much. We really appreciate the time you've taken to participate in this study.

For peer review only

Topic Guide 4: FGD -data dashboard

Topic guide for NeoTree focus groups based on Theoretical Framework of Acceptability (TFA) and the Theoretical Domains Framework (TDF)

Target behaviours – Interpreting data from data dashboard; changing clinical practise/behaviours to achieve agreed quality improvement goals.

Timing of focus group: implementation phase (months 10/11)

Participants: Healthcare Professionals

Version 1.0

Introduction

Hello, thank you very much for taking the time to speak to me today. My name is ______ and I work for _____________. This discussion will probably between 1-2 hours. As a reminder, I am talking to you today, as I work for a study that aims to improve the care given to sick and vulnerable babies in hospitals.

Before we start, can I check whether you have:

- been told about the study
- had an information sheet
- signed a consent form
- agreed to audio recording of the discussion

At this point, do you have any questions about the purpose of the study, or the documents you've been given [e.g. PIS/ consent form]? Is there anything that isn't clear?

I just want to remind you that your participation in this discussion is entirely voluntary [i.e. it is your choice].

I am interested in your views about the implementation of the NeoTree at [health facility]. Your opinions as health care workers at the frontline of newborn care are very important to us. There are no right or wrong answers to these questions, just differing points of views. Please feel free to share your point of view even if it differs from what others have said. Keep in mind that we're just as interested in negative comments as positive comments

Please give everyone a chance to speak, and please do not share what we discuss today outside this group. Please say your ID number before you speak and do not say names during the discussion as we want to keep them anonymised.

If you want to take a break or stop at any point, please tell me. And if you wish to withdraw from the study you are completely free to do so at any point.

Before we start, do you have any questions for me?

For peer review only

1) Intervention coherence (TFA) / Knowledge (TDF)

   a) Have you noticed any recent changes to the NeoTree? Any recent additions?

      [show data dashboard]

   b) Can you talk me through the dashboard and what you think it hopes to achieve?

2) Knowledge & Skills (TDF)

   a) Overall, how easy or difficult is it to understand the information presented in the dashboards? What in particular is easy? What in particular is difficult?

   b) What would make the dashboards easier to understand?

3) Beliefs about consequences (TDF)

   a) How might having these dashboards change practice, if at all?

      Prompt- how do they influence your decision making/actions?

   b) What are the benefits of having these dashboards? Any downsides?

4) Burden (TFA)

   a) To what extent do you use the dashboard in your day-to-day job? Talk me through this

   b) Has using it had an impact on your workload in any way? i.e. generated more tasks, needed time away from other responsibilities etc

5) Affective attitude (TFA) & Emotion (TDF)

   a) Do you like using the data dashboards ? What in particular do you like or dislike?

   b) How helpful is the dashboard? Any concerns or worries?

6) Social influences (TDF)

   a) To what extent do the views of others influence if and how you use the dashboard? For example the opinions of other nurses in the unit, the NeoTree ambassador, doctors, the NeoTree project management team, parents/guardians?

   b) Has the NeoTree changed team working in the unit in any way?

      Prompt: communication, roles, responsibilities?

7) Opportunity costs (TFA)

   a) Is there anything that you feel you must give up in order to use the data dashboard?

   b) Given the choice between the basic NeoTree and NeoTree with data dashboard which would you choose to use, and why?

For peer review only

8) Ethicality (TFA) & Social/Professional role & identity (TDF)

a) Do you feel displaying data and quality improvement targets in the ward is fair? for you as a HCW?

b) Do you feel it is your responsibility to use a tool like this? Who else might be responsible to use the data dashboards?

9) Self-efficacy (TFA) & Beliefs about capabilities (TDF)

a) How confident are you using the data dashboard? What in particular are you more/less confident about?

b) What could help improve your confidence?

10) Environmental context and resources (TDF)

a) Do you have enough time to use the data on the data dashboard in your day-to-day practice?

b) Do you have the necessary resources available to work towards achieving the dashboard recommendations and targets?

11) Memory, attention, decision making (TDF)

a) Is there anything on the dashboards that grab your attention? What in particular?

b) How easy/difficult is it to extract key information from the dashboard?

c) Are there ever any instances when you decided to deviate from suggested behaviour and quality improvement target? Talk me through this....

12) Goals (TDF)

a) How important is using the data dashboard for you? And why?

b) Compared to other things you have to do, where does the data dashboard fit in terms of priority? What are the competing priorities?

c) Do you have any targets/goals for practice? How does the data dashboard fit in with that if at all?

13) Perceived effectiveness (TFA) + Optimism (TDF)

a) How likely is the data dashboard to improve quality of newborn care?

14) Reinforcement (TDF)

a) What positive experiences have you had with the data dashboard that would encourage you to keep using it?

b) What incentives / rewards / pressures are there to use the data dashboard and achieve behaviour change targets?

For peer review only

15)Intentions (TDF)

a) To what extent do you intend to use the dashboards in your everyday work? Can you explain your reasons?

16)Behavioural regulation (TDF)

a) To what extent has using the data dashboard become habitual (i.e. something that you do routinely) in your day to day practice? Why/how so?
b) Have you ever encountered any problems using the dashboards? What were these and how did you overcome these?
c) What can we do to improve the implementation of the dashboards on the ward??

To summarise, the main points from our discussion are (facilitator to summarise key points). Is there anything else you'd like to add? Is there anything you'd like to ask me?

Thank you so much. We really appreciate the time you've taken to participate in this study.

For peer review only

Topic Guide 5: FGD - activated non-emergency clinical algorithm

Topic guide for NeoTree focus groups based on Theoretical Framework of Acceptability (TFA) and the Theoretical Domains Framework (TDF)

Target behaviours – use of the non-emergency algorithm to diagnose and manage newborns with sepsis and HIE.

Timing of focus group: implementation phase (month 16)

Participants: Healthcare Professionals

Version: 1.0

Introduction

Hello, thank you very much for taking the time to speak to me today. My name is ______ and I work for _____________. This discussion will probably between 1-2 hours. As a reminder, I am talking to you today, as I work for a study that aims to improve the care given to sick and vulnerable babies in hospitals.

Before we start, can I check whether you have:

- been told about the study
- had an information sheet
- signed a consent form
- agreed to audio recording of the discussion

At this point, do you have any questions about the purpose of the study, or the documents you've been given [e.g. PIS/ consent form]? Is there anything that isn't clear?

I just want to remind you that your participation in this discussion is entirely voluntary [i.e. it is your choice].

I am interested in your views about the implementation of the NeoTree at [health facility]. Your opinions as health care workers at the frontline of newborn care are very important to us. There are no right or wrong answers to these questions, just differing points of views. Please feel free to share your point of view even if it differs from what others have said. Keep in mind that we're just as interested in negative comments as positive comments

Please give everyone a chance to speak, and please do not share what we discuss today outside this group. Please say your ID number before you speak and do not say names during the discussion as we want to keep them anonymised.

If you want to take a break or stop at any point, please tell me. And if you wish to withdraw from the study you are completely free to do so at any point.

Before we start, do you have any questions for me?

For peer review only

1) Intervention coherence (TFA) / Knowledge (TDF)

a) Have you noticed any recent changes to the NeoTree? Any recent additions?

[show diagnostic tool]

b) Can you talk me through the diagnostic tool and what you think it hopes to achieve?

2) Knowledge & Skills (TDF)

a) Overall, how easy or difficult to use the non-emergency diagnostic tool? What in particular is easy? What in particular is difficult?

b) What would make the diagnostic tool easier to use?

3) Beliefs about consequences (TDF)

a) How might having the diagnostic tool change practice, if at all?

Prompt- how might it influence your decision making/actions?

b) What are the benefits of having the diagnostic tool? Any downsides?

4) Burden (TFA)

a) How does the new diagnostic tool affect your workload?

Prompt: Do you have any extra tasks with the addition of the diagnostic tool? Does it require more effort/ time compared to when the NeoTree did not have this function?

5) Affective attitude (TFA) and Emotion (TDF)

a) Do you like using the diagnostic tool? What in particular do you like or dislike?

Optional Prompt: Any concerns or worries?

6) Social influences (TDF)

a) To what extent do the views of others influence if and how you use the diagnostic tool? For example the opinions of other nurses in the unit, the NeoTree ambassador, doctors, the NeoTree project management team, parents/guardians?

b) Has the diagnostic tool changed team working in the unit in any way?

Prompt: communication, roles, responsibilities?

7) Opportunity costs (TFA)

a) Is there anything that you feel you must give up in order to use the new diagnostic tool?

8) Ethicality (TFA) & Social/Professional role & identity (TDF)

a) Do you feel the use of the NeoTree to diagnose and manage sepsis and HIE is safe? For you and for babies?

b) How does NeoTree fit in with your ways of working? Do you feel it is your responsibility to use a tool like this? Who else might be responsible for using the diagnostic tool?

For peer review only

9) Self-efficacy (TFA) Beliefs about capabilities (TDF)

a) How confident do you feel using the diagnostic tool?
b) Is there anything that could help you become more confident?

10) Environmental context and resources (TDF)

a) Do you have enough time to use the diagnostic tool?
b) Do you have the necessary resources available to use the diagnostic tool?

11) Memory, attention, decision making (TDF)

a) Do you use the diagnostic tool to guide your decision-making? Does it make things easier / more difficult? How so?
b) Have you ever forgotten to use the diagnostic tool?

If yes - in what kind of situations?

c) Are there ever any instances when you decided to deviate from the 1) the recommended diagnosis 2) the recommended management plan. Talk me through this....
d) Are there ever any instances where you decided not to use the diagnostic tool? If so, can you talk me through this...

12) Goals (TDF)

a) How important is using the diagnostic tool for you? And why?
b) Compared to other things you have to do, where does the diagnostic tool fit in terms of priority? What are the competing priorities?
c) Do you have any targets/goals for practice? How does the diagnostic tool fit in with that if at all?

13) Perceived effectiveness (TFA) and optimism (TDF)

a) How likely is the diagnostic tool to improve quality of newborn care?

14) Reinforcement (TDF)

a) What positive experiences have you had with the diagnostic tool that would encourage you to keep using it? Are there any negative experiences that would discourage you?
b) What incentives / rewards / pressures are there to use the diagnostic tool?

15) Intentions (TDF)

a) Do you intend to use the diagnostic tool in your day-to-day work? Can you explain your reasons?

For peer review only

16)Behavioural regulation (TDF)

a) To what extent has using the diagnostic tool become habitual (i.e. something that you do routinely) in your day to day practice? Why/how so?

b) Have you ever encountered any problems using the diagnostic tool? What were these and how did you overcome these?

c) What can we do to improve the use of the diagnostic tool on the ward?

To summarise, the main points from our discussion are (facilitator to summarise key points). Is there anything else you'd like to add? Is there anything you'd like to ask me?

Thank you so much. We really appreciate the time you've taken to participate in this study.

For peer review only

Topic Guide 6: FGD - sustainability

Topic guide for NeoTree focus groups based on Theoretical Framework of Acceptability (TFA) and the Theoretical Domains Framework (TDF)

Target behaviours – Use of all functions of the NeoTree as part of routine care (without support of NeoTree team)

Timing of focus group: sustainability phase (months 16-21)

Participants: Healthcare Professionals

Version: 1.0

Introduction

Hello, thank you very much for taking the time to speak to me today. My name is ______ and I work for _____________. This discussion will probably between 1-2 hours. As a reminder, I am talking to you today, as I work for a study that aims to improve the care given to sick and vulnerable babies in hospitals.

Before we start, can I check whether you have:

- been told about the study
- had an information sheet
- signed a consent form
- agreed to audio recording of the discussion

At this point, do you have any questions about the purpose of the study, or the documents you've been given [e.g. PIS/ consent form]? Is there anything that isn't clear?

I just want to remind you that your participation in this discussion is entirely voluntary [i.e. it is your choice].

I am interested in your views about the implementation of the NeoTree at [health facility]. Your opinions as health care workers at the frontline of newborn care are very important to us. There are no right or wrong answers to these questions, just differing points of views. Please feel free to share your point of view even if it differs from what others have said. Keep in mind that we're just as interested in negative comments as positive comments

Please give everyone a chance to speak, and please do not share what we discuss today outside this group. Please say your ID number before you speak and do not say names during the discussion as we want to keep them anonymised.

If you want to take a break or stop at any point, please tell me. And if you wish to withdraw from the study you are completely free to do so at any point.

Before we start, do you have any questions for me?

For peer review only

1) Introductory questions

NeoTree has been implemented on this unit/ward for X months now.

a) How much, if at all, do you think NeoTree is still used in day-to-day practice at the moment?

b) Has this changed over time (i.e. increased/ decreased)?

2) Knowledge & Skills (TDF)

a) How easy or difficult is it to use the NeoTree? Is it more or less easy to use the NeoTree since the NeoTree team have left?

b) Do you think any additional training, information or support is needed to continue to use NeoTree now that the research team are no longer present/ have left?

If so, what would be needed?

3) Beliefs about consequences (TDF)

a) Do you think there are any benefits to continuing to use the NeoTree in future?

b) Do you think there are any drawbacks to continuing to use the NeoTree in future?

c) Do the pros outweigh the cons?

4) Burden (TFA)

a) Has your role changed at all since the team has left? How?

b) How does the NeoTree affect your capacity to do your job? Does it help or hinder you to do your job?

5) Affective attitude (TFA) and Emotion (TDF)

a) Do you like using NeoTree? What in particular do you like or dislike now that the team has left?

b) Have your feelings about using the NeoTree changed since the team has left?

6) Social influences (TDF)

a) Do you have any concerns or fears about using the NeoTree now that the team has left?

b) Do the views of others influence if and how you use the NeoTree? For example the opinions of other nurses in the unit, the NeoTree ambassador, doctors, parents/guardians

c) Since the team left, has the NeoTree changed team working in the unit? In what way? (e.g. communication, roles. Responsibilities?)

Optional prompt: Any disagreements/ conflict?

For peer review only

7) Opportunity costs (TFA)

a) Is there anything that you feel you must give up in order to use the NeoTree? Do you feel that you have given up anything new since the team has left?

b) Given the choice, would you prefer the team to return? Can you explain your reasons?

8) Ethicality (TFA) Social/Professional role & identity (TDF)

a) Do you feel it is safe to continue to use NeoTree? For you and for babies? Do you feel that the NeoTree is more/ less safe since the team has left?

b) Do you feel using the NeoTree is fair now that the team has left?

c) Now that the team has left, how does NeoTree fit in with your ways of working?

d) Going forward, do you feel it is your responsibility to continue to use the NeoTree? Who else might be responsible to ensure the sustained use of the NeoTree?

9) Self-efficacy (TFA) Beliefs about capabilities (TDF)

a) Since the team has left, are you more or less confident to use the NeoTree?

b) Which aspects of the NeoTree are you confident about?

c) Which aspects are you less confident about? What could help to improve your confidence going forward?

10)Environmental context and resources (TDF)

a) Now that the team has left, do you have enough time to use the NeoTree?

b) Do you have the necessary resources to continue to use the NeoTree in future?

What else would be needed?

11)Memory, attention, decision making (TDF)

a) Have you ever forgotten to use the NeoTree since the team has left? Talk me through this

b) Since the team left, have there been instances when you decided not to use the NeoTree? Talk me through this, what did you do instead?

c) Since the team left, have there been instances when you decided not to follow the guidance of the NeoTree? Talk me through this, what did you do instead?

12)Goals (TDF)

a) How important is using NeoTree for you? And why? Has this changed since the team left?

b) Compared to other things you have to do, where using NeoTree fit in in terms of a priority? What are the competing priorities?

c) Do you have any targets/goals for future practice? How does NeoTree fit in with that if at all?

13)Perceived effectiveness (TFA) + Optimism (TDF)

a) Going forward, how likely is the NeoTree to improve quality of newborn care?

14)Reinforcement (TDF)

a) What positive experiences have you had with the NeoTree that would encourage you to use it in the long term? Are there any negative experiences that would discourage you from using NeoTree in the long term?

b) Are there any incentives / rewards / pressures to continue to use NeoTree in future?

15)Intentions (TDF)

a) To what extent do you intend to use NeoTree in the future? Why/ why not?

16)Behavioural regulation (TDF)

a) Have you encountered any problems using NeoTree since the team has left? How did you overcome these?

b) What needs to be done to support use of NeoTree longer term? (what, by whom, where, when?)

To summarise, the main points from our discussion are (facilitator to summarise key points). Is there anything else you'd like to add? Is there anything you'd like to ask me?

Thank you so much. We really appreciate the time you've taken to participate in this study.

For peer review only

For peer review only

Supplementary File 2

Draft topic guides for semi-structured interviews (SSIs) with senior managers and clinical staff

SSI guide 1 – Baseline

Interview guide based on Theoretical Framework of Acceptability/ Theoretical Domains Framework (TDF).

Target behaviour: intention to introduce and implement the NeoTree

Timing of interviews: pre- implementation phase (months 5-6)

Participants: senior managers/ administrative staff/ clinicians

Version 1.0

Introduction

Hello, thank you very much for taking the time to speak to me today. My name is ______ and I work for _____________. This interview will probably last around 1 hour. As a reminder, I am talking to you today, as I work for a study that aims to improve the care given to sick and vulnerable babies in hospitals.

Before we start, can I check whether you have:

- been told about the study
- had an information sheet
- signed a consent form
- agreed to audio recording of the discussion

At this point, do you have any questions about the purpose of the study, or the documents you've been given [e.g. PIS/ consent form]? Is there anything that isn't clear?

I want to reassure you that I work for a research organisation [name] and not [name of health facility]. Anything you tell me today is confidential and will not be shared with your colleagues. All personal and identifying information (such as your name/names of others) mentioned will be removed and replaced with a code.

I just want to remind you that your participation in this interview is entirely voluntary [i.e. it is your choice]. If you do not want to answer a question you can just say 'pass' and we will move on to the next question.

I am interested in your views about the introduction of the NeoTree at [health facility]. There are no right or wrong answers to these questions; I am just interested in your views so please answer honestly.

If you want to take a break or stop at any point, please tell me. And if you wish to withdraw from the study you are completely free to do so at any point.

Before we start, do you have any questions for me?

For peer review only

1) Warm up questions

What is your position at this hospital? How long have you worked here?

2) Introduction/ demonstration of the NeoTree

The NeoTree is a tablet-based clinical app, NeoTree, which combines evidence-based clinical guidelines with real-time data collection and education about newborn clinical needs. The NeoTree aims to improve quality of care and newborn survival through:

A) data-capture,

B) emergency decision-support

C) non-emergency clinical and management decision-support

D) feedback of data to dashboards and national aggregate data systems.

Health care workers (HCWs) use the app at the bedside to admit and discharge each newborn patient. As they complete the admission, they receive prompts to respond appropriately to the data they have entered and manage patients according to evidence-based guidelines. We also plan to link to national data systems (Electronic Medical Records).

Do you have any questions?

1) Knowledge & Skills (TDF)

a) How easy or difficult do you think it would be to introduce the NeoTree at [HEALTH FACILITY]?

Prompt: What in particular would be easy? What in particular would be difficult?

b) Do you think the staff would require any additional skills or training to use/ manage NeoTree?

2) Beliefs about consequences (TDF)

a) What do you perceive to be the benefits/positives of introducing the NeoTree at [HEALTH FACILITY]?

Prompt: for: you, other colleagues/roles and for patients & families?

b) Would there be any drawbacks?

Prompt: for you, other colleagues/roles and for patients and families?

c) Overall, do you think the pros would outweigh the cons?

3) Burden (TFA + Social Professional Role/Identity (TDF)

a) Do you think using the NeoTree would create extra work for you, for other staff? In what way?

b) Do you feel it is your responsibility oversee the introduction of the NeoTree? Who else might be responsible?

For peer review only

4) Affective attitude (TFA) and Emotion (TDF)

a) In general, how would you feel about the introduction and use of the NeoTree at [health facility]?

Positive emotions: pride, confidence, satisfaction, reassurance
Negative emotions; guilt, worry, concern, pressure

b) Do you have any worries or concerns about introducing digital aids such as the NeoTree into clinical care at this hospital?

5) Social influences (TDF)

a) Do you know of other hospitals using NeoTree or digital innovations? To what extent has this impacted on your decision to introduce the NeoTree in this facility?

6) Perceived Opportunity costs (TFA)

a) Do you think introducing the NeoTree would be a good use of resources?

7) Perceived Self-efficacy (TFA) Beliefs about capabilities (TDF)

a) To what extent do you feel confident staff in this facility could implement Neotree? What else would be needed?

8) Ethicality (TFA)

a) Do you feel the NeoTree would be safe? For you and for babies?

b) Do you feel introducing the NeoTree would be fair for you? For other staff?

9) Environmental context and resources (TDF)

a) Do you think you would have sufficient resources to implement the NeoTree?

10) Goals (TDF)

a) Compared to other things you have to do, where does introducing the NeoTree fit in in terms of a priority? What would be the competing priorities?

b) Do you have any targets/goals for practice? How would the NeoTree fit in with that if at all?

11) Perceived effectiveness (TFA) + optimism (TDF)

In your view, how likely is the NeoTree to improve quality of newborn care at [HEALTH FACILITY]?

12) Intentions (TDF)

a) Would you like this hospital to use the NeoTree? Can you explain your reasons why/why not?

13) Barriers/ facilitators (general)

a) What do you think might be some of the barriers to introducing the NeoTree at [HEALTH FACILITY]?

For peer review only

b) Is there anything that might make it easier to implement the NeoTree at [HEALTH FACILITY]?

14) Closing questions/ remarks

a) Now that we've completed the interview, is there anything you'd like to ask me or do you have anything further to add?

Thank you so much, we really appreciate the time you've taken to participate in this study.

For peer review only

SSI guide 2 - Implementation

Interview guide based on Theoretical Framework of Acceptability/ Theoretical Domains Framework (TDF).

Interview guide to explore (A) perceptions and understanding of quality newborn care and (B) acceptability of Neotree/ barriers and facilitators to implementation

Timing of interviews: implementation phase (months 6-16)

Participants: senior managers/ administrative staff/ clinicians

Version 1.0

Introduction

Hello, thank you very much for taking the time to speak to me today. My name is ______ and I work for _____________. This interview will probably last around 1 hour. As a reminder, I am talking to you today, as I work for a study that aims to improve the care given to sick and vulnerable babies in hospitals.

Before we start, can I check whether you have:

- been told about the study
- had an information sheet
- signed a consent form
- agreed to audio recording of the discussion

At this point, do you have any questions about the purpose of the study, or the documents you've been given [e.g. PIS/ consent form]? Is there anything that isn't clear?

I want to reassure you that I work for a research organisation [name] and not [name of health facility]. Anything you tell me today is confidential and will not be shared with your colleagues. All personal and identifying information (such as your name/names of others) mentioned will be removed and replaced with a code.

I just want to remind you that your participation in this interview is entirely voluntary [i.e. it is your choice]. If you do not want to answer a question you can just say 'pass' and we will move on to the next question.

I am interested in your views about the implementation of the NeoTree at [health facility]. There are no right or wrong answers to these questions; I am just interested in your views so please answer honestly.

If you want to take a break or stop at any point, please tell me. And if you wish to withdraw from the study you are completely free to do so at any point.

Before we start, do you have any questions for me?

1) Warm up questions

What is your position at this hospital? How long have you worked here?

For peer review only

2) Quality of newborn care

a) What does high quality newborn care mean to you?

Prompt- What do you understand by high quality care?

b) Who do you see as the providers of care in the neonatal unit?

Prompt - nurses, students, doctors, mother, other family members

Now I'd like to ask you some questions about the NeoTree. I'm really interested to understand your views and feelings with regard to the NeoTree.

3) Intervention coherence (TFA)

a) Can you talk me through what your understanding of NeoTree is? What it involves, its purpose, who it is intended to be used by, when, where etc?

4) Barriers/ enablers (general)

a) What have been the barriers to implementing NeoTree in this hospital?

b) What would help make it easier to implement the NeoTree here?

5) Experienced affective attitude (TFA) and Emotion (TDF)

a) In general, how do you feel about the introduction and use of the NeoTree at [health facility]?

Positive emotions: pride, confidence, satisfaction, reassurance
Negative emotions; guilt, worry, concern, pressure

b) Specifically, how useful do you find the different functionalities of the NeoTree:

- Data capture (i.e. admission, discharge and lab data);
- Diagnostic support (emergency and non-emergency);
- Data dashboards and quality improvement goals
- Data linkage to local and national databases

c) Do you have any worries or concerns about introducing digital aids such as the NeoTree into clinical care at this hospital?

6) Experienced burden (TFA)

a) Has the NeoTree required any extra time/ work/ resources? For you personally, from your staff?

7) Ethicality (TFA)

a) Do you think that the NeoTree is safe? For staff, for patients? Any concerns/ worries?

b) Has the NeoTree led to any changes in team working and communication? Have these been positive or negative? Any divisions between staff or conflicts?

For peer review only

8) Experienced opportunity costs (TFA)

a) Do you think the NeoTree is a good use of resources? Why/why not?

9) Experienced effectiveness (TFA)

a) At this stage of implementation to what extent do you feel the NeoTree has improved quality of newborn care? What in particular has helped improve quality of care?

10) Experienced self-efficacy (TFA)

a) What is your role in the implementation of the NeoTree?
b) How confident do you feel in carrying out this role? Which aspect of your role do you like/ and which do you like less?
c) To what extent do you feel confident staff in this facility can implement Neotree? What else would be needed?

11) Social influences (TDF)

a) Do you know of other hospitals using NeoTree or digital innovations? Did that impact on your decision to implement NeoTree in this facility at all?

12) Intentions (TDF)

a) To what extent do you plan to use the NeoTree in the future? Can you explain your reasons?

13) Behavioural regulation (TDF)

a) Have you ever encountered any problems in implementing/introducing NeoTree to this facility? If so, can you talk me through these? How did you overcome these?
b) Is there anything you think we can do to improve the implementation of the NeoTree in this hospital?

14) Closing questions/ remarks

a) In your view, what 5 things are needed to improve quality of neonatal care in this hospital?
b) Now that we've completed the interview, is there anything you'd like to ask me or do you have anything further to add?

Thank you so much, we really appreciate the time you've taken to participate in this study.

For peer review only

SSI Guide 3 - Sustainability

Interview guide to explore acceptability of Neotree/ barriers and facilitators to sustainability. Based on Theoretical Framework of Acceptability (TFA)/ Theoretical Domains Framework (TDF)

Timing: Sustainability phase (months 16-21)

Participants: senior managers/ administrative staff/ clinicians

Version: 1.0

Introduction

Hello, thank you very much for taking the time to speak to me today. My name is ______ and I work for _____________. This interview will probably last around 1 hour. As a reminder, I am talking to you today, as I work for a study that aims to improve the care given to sick and vulnerable babies in hospitals.

Before we start, can I check whether you have:

- been told about the study
- had an information sheet
- signed a consent form
- agreed to audio recording of the discussion

At this point, do you have any questions about the purpose of the study, or the documents you've been given [e.g. PIS/ consent form]? Is there anything that isn't clear?

I want to reassure you that I work for a research organisation [name] and not [name of health facility]. Anything you tell me today is confidential and will not be shared with your colleagues. All personal and identifying information (such as your name/names of others) mentioned will be removed and replaced with a code.

I just want to remind you that your participation in this interview is entirely voluntary [i.e. it is your choice]. If you do not want to answer a question you can just say 'pass' and we will move on to the next question.

I am interested in your views about the implementation of the NeoTree at [health facility]. There are no right or wrong answers to these questions; I am just interested in your views so please answer honestly.

If you want to take a break or stop at any point, please tell me. And if you wish to withdraw from the study you are completely free to do so at any point.

Before we start, do you have any questions for me?

For peer review only

1) Warm up questions

How are you? How have you been since we last spoke? Any change in terms of your role and responsibilities at the hospital?

The last time we spoke, I asked you a series of questions about your views and experiences of using the NeoTree. Since then, the NeoTree project managers and developers have left and no longer provide support on site.

I'd like to ask you some questions about how things have been since they left.

2) Intervention coherence (TFA)

a) To what extent do you think NeoTree is currently used/ has this changed over time/ why / how so
b) Has there been anything that has made it harder to implement NeoTree and sustain its use?
c) Anything that has made it easier?

3) Experienced affective attitude (TFA) & Emotions (TDF)

a) Have your feelings about the NeoTree changed since we last spoke [prompt previous answer].
b) Do you have any concerns or fears about implementing the NeoTree in this hospital now that the team has left

4) Experienced Burden (TFA)

Since we last spoke [prompt previous answers] do you think the NeoTree:

a) Has generated any extra work in terms of supervision or staffing?
b) Has required any extra time. Extra resources? from you personally, from your staff?
c) Since we last spoke, has the NeoTree diverted staff/ equipment/ funds away from other aspects of patient care? If so, what impact has this had on you personally, on other staff and on patients?

5) Ethicality (TFA)

a) Now that the team has left, do you think that the NeoTree is safe? For staff, for patients? Any changes/ concerns since we last spoke?
b) Since the last interview, do you think the NeoTree has led to any changes in team working and communication? Have these been positive or negative?

6) Experienced opportunity costs (TFA)

a) Do you think the NeoTree is a good use of resources? Why/why not? Has your opinion changed since we last spoke?

For peer review only

7) Experienced effectiveness (TFA)

a) Since we last spoke, do you feel the NeoTree has led to any further improvements in quality of care? What in particular has helped/ hindered quality of care?

8) Experienced self-efficacy (TFA)

a) Last time you told me that your role in the NeoTree is [insert from previous interview]. Has this changed since our last interview?

b) How confident do you feel in carrying out this role? Do you feel more or less confident since our last interview? Why?

c) To what extent do you feel confident staff in this facility can sustain NeoTree in the future? What else would be needed?

9) Intentions (TDF)

a) To what extent do you intend to sustain the NeoTree at this facility? Can you explain your reasons to me?

10) Barriers and enablers to sustainability (general)

a) What would it take to continue to use NeoTree going forward? What might get in the way of sustainability?

b) Is sustaining NeoTree a priority given other demands at this hospital?

c) What could help support sustainability longer term?

11) Closing questions/ remarks

a) Now that we've completed the interview, is there anything you'd like to ask me or do you have anything further to add?

Thank you so much, we really appreciate the time you've taken to participate in this study.

For peer review only

Supplementary File 3:

Parent/ Carer Interview Guide to explore (A) perceptions and understanding of quality newborn care and (B) acceptability of Neotree

Timing of interviews: implementation phase (months 6-16)

Version 1.0

Introduction

Hello, thank you very much for taking the time to speak to me today. My name is ______ [name of researcher] and I work for ______________. This interview will probably last around 1 hour. As a reminder, I am talking to you today, as I work for a study that aims to improve the care given to sick and vulnerable babies in hospitals.

Before we start, can I check whether you have:

- been told about the study
- had an information sheet
- signed a consent form
- agreed to audio recording of the discussion

At this point, do you have any questions about the purpose of the study, or the documents you've been given [e.g. PIS/ consent form]? Is there anything that isn't clear?

I want to reassure you that I work for a research organisation [name] and not [name of health facility]. Anything you tell me today will not affect the care of your baby or your family. All personal and identifying information (such as your name/names of others) mentioned will be removed and replaced with a code.

I just want to remind you that your participation in this interview is entirely voluntary [i.e. it is your choice]. If you do not want to answer a question you can just say 'pass' and we will move on to the next question.

I am interested in your views about the care of sick newborns, and your experiences of receiving care in this hospital. There are no right or wrong answers to these questions; I am just interested in your views so please answer honestly.

If you want to take a break or stop at any point, please tell me. And if you wish to withdraw from the study you are completely free to do so at any point.

As a reminder, your answers to these questions will not affect the care you or your family will receive at all. Do you have any questions for me?

For peer review only

1) Warm up questions

How are you feeling today? How long have you/ your baby/ family member been in the hospital? Where have you travelled from?

2) Quality of newborn care

a) What does high quality newborn care mean to you?

Prompt: What do you understand by high quality newborn care?

b) In your view, who provides most of the care in the neonatal unit?

Prompt: nurses, students, doctors, mothers, family members etc..

Acceptability of current care and use of digital aids

Now I'd like to ask you some questions about the NeoTree digital health aid. You probably noticed that when the HCW asked you questions about you/ your baby/ family member she/ he was using a tablet with the NeoTree app.

The NeoTree app uses the information that you give to the healthcare worker to work out what is likely to be wrong with the baby and how to best treat and manage the baby. Whilst the baby is on the newborn care unit it will also provide an electronic linkage to any tests that are done to check for infection. As well as providing information on what might be wrong with the baby, the NeoTree app also gives the healthcare workers education and tips around how to care for the newborn. The NeoTree also stores the clinical information for all of the babies and then feeds back to the healthcare workers and the hospital on things like how many babies were admitted each month and what was wrong with them so that they can best plan the services needed.

3) Experienced affective attitude (TFA) and Emotions (TDF)

Gathering and storing health information

a) How did you feel about answering questions about you and about your baby's health?

➢ Prompts to illicit feelings/ emotions:
Positive emotions: pride, confidence, satisfaction, reassurance
Negative emotions; guilt, worry, concern, pressure

b) How did you feel about the HCW entering your answers into the tablet [show visual aid]? Did you have any concerns about this at all?

c) Once your answers were entered into the tablet, your answers were stored electronically. How did you feel about the HCW storing (keeping) your answers on the tablet [show visual aid]?

Diagnosis and management of newborns

- How do you feel about the use of the NeoTree to support HCWs to diagnose and treat sick babies? [show example] Any concerns or worries?

For peer review only

4) Experienced burden (TFA)

a) How easy or difficult was it to answer the HCW's questions, when your baby was admitted using the NeoTree?

b) Were there any questions that were difficult or complicated to answer?

c) Do you think the NeoTree helped the HCWs provide care more efficiently to your baby? Did it get in the way at all? How so?

5) Experienced effectiveness (TFA)

a) Do you think the NeoTree was helpful for HCWs to provide care to babies? How so?

b) Do you feel the NeoTree has helped in the care of your baby? In what way?

c) Have you experienced any challenges while your baby has been in the unit?

6) Ethicality (TFA)

a) Did you feel fairly treated while they were using the NeoTree? Would you change any aspect of the way you were treated while they were using the NeoTree?

b) Do you think the NeoTree distracted HCWs from caring for your baby?

c) Do you have any safety concerns about the use of the NeoTree in the care of your baby?

7) Intervention coherence (TFA)

a) How clear was the information you were given about your baby's care [treatment/procedures]? Was anything not clear/ difficult to understand?

b) How do you think the data/information about you and your baby's health is used? What do you think happens to this information?

c) Do you think the NeoTree can help improve quality of newborn care? In what way?

8) Experienced opportunity costs (TFA)

a) Do you think there are any risks to you or your baby when the HCW uses NeoTree?

b) If you had the choice, would you prefer HCWs to record information about you and your baby on paper (i.e. not use the NeoTree)? If so why?

c) If you had the choice, would you prefer HCWs diagnose and manage your baby without the help of the NeoTree? If so, why?

9) Experienced self-efficacy (TFA)

a) How confident did you feel speaking to healthcare workers about the care of your baby?

b) How involved did you feel involved in the care of you baby? Clinical decisions etc?

For peer review only

10) Closing questions/ remarks

a) If you could change one thing about the care and treatment you've received at the unit, what would you change?

- Now that we've completed the interview, is there anything you'd like to ask me?

Thank you so much, we really appreciate the time you've taken to participate in this study.

For peer review only

For peer review only

Tool for collecting Non-Staff cost items. Adapted from "Haghparast-Bid

**Item/activity description** (Description of each item or activity should be specific and detailed)*	**Year**

Notes

* Among the non-staff costs, travel and overhead/joint costs will be the

** For items (such overhead, rent and running costs of an office, equipr

overhead or running cost of an office, you can use for example, % of pe

For peer review only

For peer review only

dgoli, Skordis J et al: Protocol for the cost-consequence and equity impact analyses o

Line Item	% allocated to NeoTree (mainly relevant to capital/equipment, rent, and office overhead)**	Date/Time period (Optional)	Cost allocated to NeoTree (Local currency) or spcify currency if different

e common cost items.
ment etc.) that are shared across different projects, you can use an allocation rule fo
eople working on the NeoTree activities, or % time spent on or space used for the Ne

For peer review only

For peer review only

Yes
No

›f a cluster randomised controlled trial comparing three variants of a nutrition-sensitive agricultura

**Is it possible to allocate the cost directly to the intervention components?** If No, the cost item will be 100% joint cost. If yes, please specify % allocated to each component.	Data Entry (Admission and Discharges/Deaths)	Data back-up	M&M dashboard preparation	Research Meetings

›r allocating them to different projects. For example, for allocating
›oTree activities.

For peer review only

For peer review only

al extension intervention to improve maternal and child dietary diversity and nutritional status in rura

NeoTree activities (%)

Any other meetings relating to NeoTree	NeoTree Support/Testing/Pilot/Roll-out	NeoTree data pipeline	Diagnosis Data Entry	Data Quality Checks/Data audits/Data cleaning	Research (paper writing, etc)	Software development

For peer review only

For peer review only

ı Odisha, India (UPAVAN trial) Trials (2019) 20:287"

MOH Database integration	Training	Clinical validation	Joint/admin	Check (should be 100%)
				0%
				0%
				0%
				0%
				0%
				0%
				0%
				0%
				0%
				0%
				0%
				0%
				0%
				0%
				0%
				0%
				0%
				0%
				0%
				0%
				0%
				0%
				0%
				0%
				0%

For peer review only

Staff time-use data collection tool. Adapted from "Haghparast-Bidgoli, Skordis J et al

Name
Position/role

Time period (e.g. Sep 2019 or Sep-Dec 2019)*	**Non-NeoTree activities (%)**	**NeoTree activities (%)**	Check- should be 100%	Data Entry (Admission and Discharges/Deaths)
			0%	
			0%	
			0%	
			0%	
			0%	
			0%	
			0%	
			0%	
			0%	
			0%	
			0%	
			0%	
			0%	
			0%	
			0%	
			0%	
			0%	
			0%	
			0%	
			0%	
			0%	
			0%	
			0%	
			0%	
			0%	
			0%	
			0%	
			0%	
			0%	

For peer review only

: Protocol for the cost-consequence and equity impact analyses of a cluster randomised c

N

Data back-up	M&M dashboard preparation	Research Meetings	Any other meetings relating to NeoTree	NeoTree Support/Testing/Pilot/Roll-out	NeoTree data pipeline

For peer review only

:ontrolled trial comparing three variants of a nutrition-sensitive agricultural extension interv

NeoTree activities (%)

Diagnosis Data Entry	Data Quality Checks/Data audits/Data cleaning	Research (paper writing, etc)	Software development	MOH Database integration	Training

For peer review only

ention to improve maternal and child dietary diversity and nutritional status in rural Odisha, India (

Clinical validation	Joint/admin	Check (should be 100%)
		0%
		0%
		0%
		0%
		0%
		0%
		0%
		0%
		0%
		0%
		0%
		0%
		0%
		0%
		0%
		0%
		0%
		0%
		0%
		0%
		0%
		0%
		0%
		0%
		0%
		0%
		0%
		0%
		0%

For peer review only

(UPAVAN trial) Trials (2019) 20:287"

For peer review only

Supplementary File 6

Data Management Procedures

When a baby is admitted, an admission form is completed on the NeoTree tablet app and an ID for the neonate is generated. The NeoTree ID generated is stored in the nurses' routine admission book along with the mother's name, acting as the data spine. Personal identifiable information (PII) is collected as part of the electronic admission form (incl. mother's name, baby's name, baby's date of birth). All data (including PII) are stored temporarily in an encrypted format on the tablet and hidden from view: only members of the research/clinical team can retrieve these data from the tablet by supplying a valid password. The identifiable NeoTree forms will be printed and inserted into clinical paper records to replace usual hand-written forms. All PII fields (e.g. mother's name, baby's name are not exported from the tablet into the NeoTree backend. PII fields are only stored in the tablet in an encrypted format.

Any other forms (e.g. discharge forms, laboratory results forms) submitted on the app are recorded against the neonates "NeoTree ID". In addition, some PII may also be stored (e.g. the mother's name) on the tablet, although this will be password protected as above, and records will be routinely wiped from the tablet as an extra safeguard.

In Zimbabwe, non-PII data from all forms are saved to a secure database at a Zimbabwean data centre: the "Clinical database". This database will be locally owned, stored and managed. The NeoTree team will have access to the database in order to ensure that the process for moving data from tablets to database is successful, and to enable a copy of the anonymized data for research purposes. (See "Anonymised research database" below.) Data moved from the tablets to the secure database will be encrypted in transit. Data are encrypted at rest in the Clinical database, where they are password protected. The only way to tie individual records in the database back to the neonate they describe is by matching the NeoTreeID with the PII, based on the paper print out stored in the patient's notes and the nurses' routine admission book. A regular process is in place to wipe data from the tablets, so encrypted identifiable data are stored for only a brief period of time (e.g. if a second copy of the print out is needed for clinical reasons). Aggregate statistics are computed on the data in the secure database, and served back to staff in the hospital via dashboards. A regular process will be put in place to copy anonymized data from the Clinical database into the "Anonymised research database", curated by the NeoTree research team and held on a UCL server. Data will be encrypted in transit and at rest. The entire approach to data security is twofold: (i) minimize the chance of data leakage, by ensuring that data is encrypted in transit and at rest, and by

For peer review only

minimizing the number of places the data is stored and (ii) pseudonomizing the data at the point of collection, so that even in the event of a breach, PII would not be leaked.

In Malawi, the process is identical except that the "clinical database" is stored on a secure virtual machine in an encrypted format using Amazon Webserver (AWS).

For peer review only

Supplementary File 7

Summary of implementation and research plan adaptation in response to COVID-19

1. Data collection.

In 2020 we submitted ethics amendments to allow for the option of remote data collection via secure platforms such as Microsoft Teams, if face-to-face data generation was deemed is not feasible due to COVID-19 restrictions. In August- December 2020, researchers conducted 17 semi-structured interviews with healthcare professionals at Chinhoyi Provincial Hospital, Zimbabwe. Most were conducted via remote methods (Microsoft Teams) due to travel restrictions which prevented on-site visits. The remainder of data collection is likely to be generated in face-to-face settings, with enhanced safety measures in line with hospital protocols, such as physical distancing, increased ventilation and mask wearing.

Routine admission and discharge data collection via the NeoTree platform has continued uninterrupted.

2. Implementation

Roll out of some functionalities was delayed for example the roll-out of the data dashboards in Zimbabwe. Clinical processes have inevitably adapted, for example reduced rotas of staff and suspension of face-to-face morbidity and mortality meetings during periods of heightened case numbers.

Nevertheless the NeoTree has proved robust in the face of these challenges. We have been highly responsive and adaptive to the pandemic, ensuring the safety of staff (for example remote support to clinical teams) while ensuring the NeoTree system has maintained function, data capture, analysis and emergency decision support for HCWs.

After media reports of increased stillbirths at Sally Mugabe Central Hosspital (SMCH) associated with COVID-19 pandemic reductions in hospital access, the NeoTree was adapted in one week at SMH to capture maternal and stillbirth outcomes. A similar process has been approved at Kamuzu Central Hospital, Malawi.

For peer review only
